# Identification of fatty acid metabolism-related genes in the tumor microenvironment of breast cancer by a development and validation of prognostic index signature

**DOI:** 10.1186/s41065-025-00425-4

**Published:** 2025-04-07

**Authors:** Zhaofeng Ma, Man Zheng, Pulin Liu

**Affiliations:** 1 Shandong University of Traditional Chinese Medicine, Jinan, Shandong Province China; 2 Dongying People’s Hospital (Dongying Hospital of Shandong Provincial Hospital Group), Dongying, Shandong Province 257091 China

**Keywords:** Breast cancer (BRCA), Fatty acid metabolism genes (FAMGs), Predicting model, Immunity, SNP, CNV, Bioinformatic

## Abstract

**Background:**

Breast cancer (BRCA) is a malignancy originating in the breast cells, characterized by a poor overall survival rate. Post-resection, chemotherapy is commonly recommended as a primary therapeutic approach; however, its efficacy remains limited. Recent advancements in lipidomics and metabolomics have provided new insights into the intricate landscape of fatty acid metabolism (FAM) and the fatty acid lipidome in both health and disease. A growing body of evidence suggests that dysregulations in FAM and fatty acid levels play a significant role in cancer initiation and progression. Despite these advances, the precise mechanisms through which FAM mediates the anti-cancer effects of lobaplatin in BRCA remain poorly understood and warrant further investigation.

**Methods:**

GEO and TCGA data were classified into two types. We aimed to show how FAMGs influence immune function, immune checkpoints, and m6a in BRCA. A co-expression analysis discovered that gene expression is strongly connected to pyroptosis. The TCGA gathered information about mRNAsi, gene mutations, CNV, and clinical features.

**Results:**

In the low-risk group, overall survival (OS) is longer. GSEA was utilized to identify immune and tumor-related pathways. Most of the FAMG-derived prognostic signatures predominantly modulate immunological and oncogenic signaling pathways, including the Wnt, neurotrophin, chemokine, and calcium signaling cascades. Among the genes involved are CEL, WT1, and ULBP2. Expression levels varied as well. The prognostic model, CNVs, single nucleotide polymorphism (SNP), and drug sensitivity all pointed to the gene.

**Conclusions:**

The primary objective of this study is to identify and validate BRCA-associated FAMGs that can serve as prognostic indicators and provide insights into immune system function, while also offering evidence to support the development of fatty acid metabolism-related molecularly targeted therapeutics. Consequently, FAMGs and their interactions with the immune system, as well as their role in BRCA, may emerge as promising therapeutic targets.

**Supplementary Information:**

The online version contains supplementary material available at 10.1186/s41065-025-00425-4.

## Introduction

Cancer mortality has continued to decline through 2021, averting over 4 million deaths since 1991 due to reductions in smoking, earlier detection of certain cancers, and advancements in both adjuvant and metastatic treatments [1]. However, these gains are increasingly threatened by rising incidence rates for six of the ten most common cancers. Between 2015 and 2019, the incidence of breast, pancreatic, and uterine corpus cancers increased by 0.6%–1% annually, while prostate, female liver, kidney, human papillomavirus (HPV)-associated oral cancers, and melanoma rose by 2%–3% annually [2]. The etiology of breast cancer (BRCA) is uncontrolled growth of mammary epithelial cells, which leads to malignant transformation [3]. According to the most current International Agency for Research on Cancer data from 2018, BRCA is the most common cancer among women worldwide, accounting for 24.0 percent of all cancers, with developing countries contributing 52.9 percent of these cases [4, 5]. Many endocrine hormones, including estrone and estradiol, target the mammary gland and have been linked to the prevalence of BRCA. Because the performance is not evident, it is extremely easy to disregard the symptoms of early BRCA. These symptoms include breast lumps, abnormal breast skin, nipple discharge, nipple or areola abnormal local symptoms [6, 7]. Cachexia may appear in advanced-stage BRCA patients, and advanced stage BRCA can produce metastasis of cancer cell far away, occurrence systemic much organ pathological changes, direct threat patient's life [8]. Breast ultrasonography serves as a primary imaging modality for the initial diagnosis and characterization of BRCA, facilitating the differentiation of tumor types. The TNM staging system is commonly employed to assess the extent of disease progression in BRCA patients. Surgical intervention remains the preferred therapeutic approach, involving either direct tumor excision or ultrasound-guided biopsy to obtain tissue samples for pathological evaluation [9, 10]. A multifaceted approach will likely remain essential for effective cancer treatment in the foreseeable future. Despite therapeutic advances, a substantial proportion of patients, particularly those with metastatic disease involving the brain and liver, have exhausted all available treatment options and face a lack of viable clinical alternatives [11]. Geographic disparities in the cost and availability of treatments further exacerbate patient outcomes. Advances in early cancer detection hold promise for enabling intervention prior to metastatic progression, potentially reducing both the burden and heterogeneity of oncogenic alterations, thereby improving treatment efficacy [12].

Fatty acid metabolism encompasses both anabolic and catabolic processes that are critical for maintaining cellular energy balance and generating metabolic intermediates essential for membrane biosynthesis and function [13]. Lipid metabolism exerts a central influence on tumor progression by regulating key cellular processes that sustain cancer cell survival, proliferation, and metastasis. Dysregulation of lipid metabolic pathways profoundly impacts membrane integrity, energy homeostasis, and signal transduction, thereby fostering a microenvironment conducive to tumor growth. Emerging evidence underscores that aberrant lipid metabolism not only supplies cancer cells with vital structural components and bioenergetic resources but also facilitates immune evasion and resistance to therapy. Accordingly, targeting lipid metabolic pathways has gained traction as a promising therapeutic strategy to curb tumor progression and enhance clinical outcomes. Recent technical advances in dissecting and researching fatty acid profiles and metabolism have yielded data on the pathogenic and protective effects of fatty acid metabolism in health and disease. The tumor microenvironment (TME) is characterized by hypoxia, oxidative stress, acidity, and nutrient deprivation, arising from rapid tumor cell proliferation and inadequate angiogenesis [14]. Consequently, cancer cells undergo metabolic reprogramming to adapt to these hostile conditions, facilitating their survival and growth even under the suppression of oncogenic signals. A hallmark of cancer is the reprogramming of energy metabolism, which is essential for sustaining cell proliferation and division. Unlike normal cells, which primarily metabolize glucose through glycolysis to pyruvate and subsequently generate substantial energy via mitochondrial oxidative phosphorylation, cancer cells preferentially convert glucose to lactate, even in the presence of oxygen—a phenomenon known as the Warburg effect [15]. This metabolic shift enhances glucose uptake and utilization, thereby supporting anabolic processes critical for tumor growth. Alterations in pyruvate metabolism, including reduced expression of mitochondrial pyruvate carriers, have been implicated in cancer initiation and progression [16]. Beyond glucose metabolism, lipid metabolism has emerged as a key factor in oncogenesis and metastasis, although conflicting evidence underscores the complexity of its role in tumor progression.

**Fig. 1:**
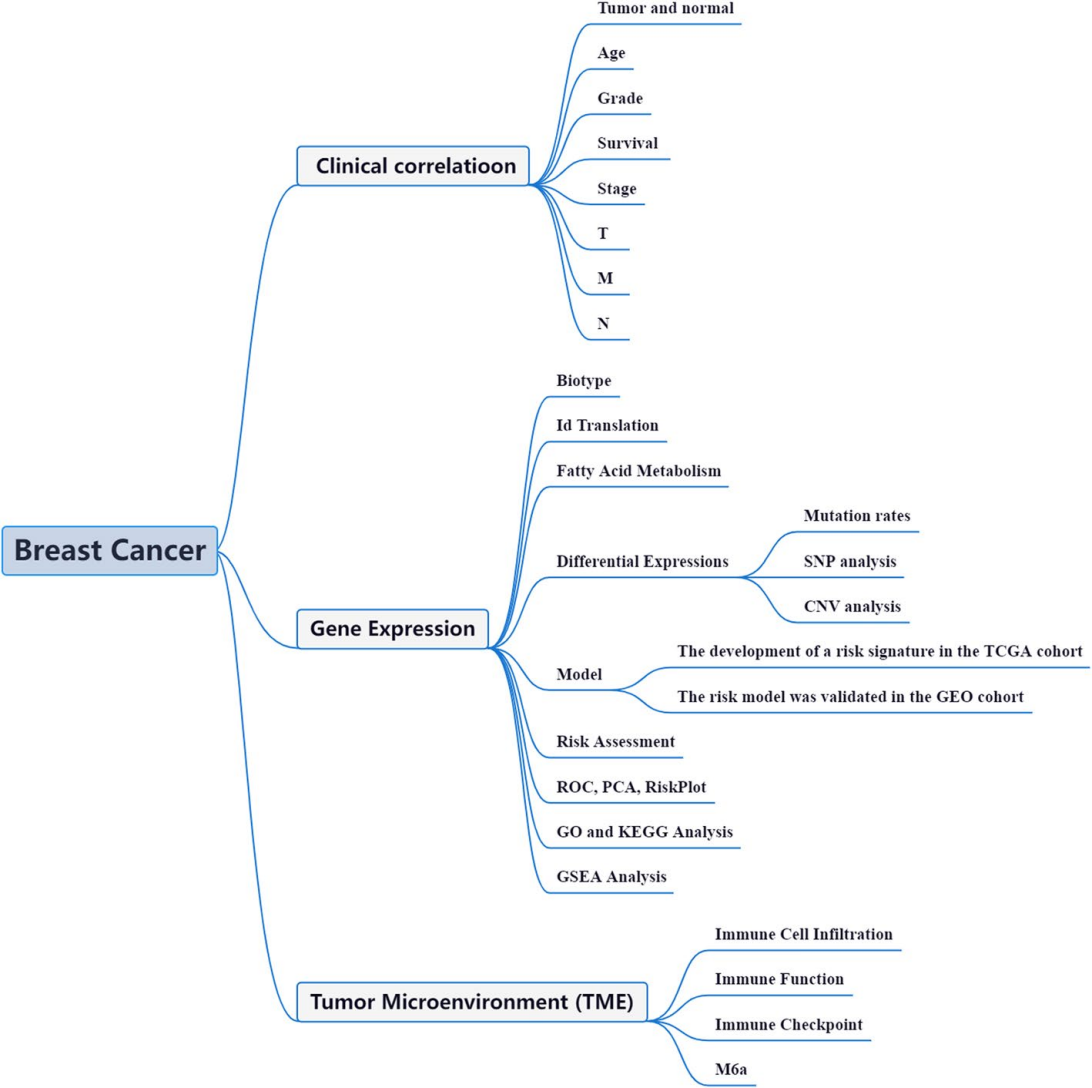
Approaches are based on a fatty acid metabolism adaptable working

RNA dysregulation plays a pivotal role in the initiation and progression of BRCA, influencing fundamental cellular processes such as transcriptional regulation, signal transduction, and immune response [17]. Aberrant RNA expression, processing, and modification disrupt oncogenic and tumor-suppressive pathways, thereby driving malignant transformation and disease progression. Non-coding RNAs (ncRNAs), including microRNAs (miRNAs), long non-coding RNAs (lncRNAs), and circular RNAs (circRNAs), have emerged as key regulators of BRCA pathophysiology. Dysregulated miRNAs modulate gene expression post-transcriptionally, targeting critical oncogenes and tumor suppressors [18]. Chemical modifications of RNA further contribute to BRCA pathogenesis, with over 172 distinct types identified to date. Among these, N6-methyladenosine (m6 A), N1-methyladenosine (m1 A), N7-methylguanosine (m7G), and 5-methylcytosine (m5 C) are the most prevalent. m6 A modification, one of the most common eukaryotic mRNA alterations, has been implicated in regulating RNA stability, splicing, and translation efficiency, influencing oncogenic signaling pathways [19]. Furthermore, immune checkpoint inhibitor (ICI) profiles in BRCA patients may facilitate more precise diagnosis, prognostic assessment, and therapy selection [20]. The BRCA Initiative, with its extensive repository of high-throughput transcriptomic data and detailed clinical annotations, provides a powerful platform for investigating altered transcriptional landscapes and the associated molecular pathways in BRCA [21]. Insights derived from this bioinformatics-driven research offer a multidimensional understanding of BRCA pathophysiology, paving the way for the development of novel diagnostic biomarkers and targeted therapeutic strategies. Figure 1 illustrates the integration of bioinformatics and cancer biology, providing a cohesive overview of this investigative framework.

## Marerials and methods

We used the methods suggested by Zi-Xuan Wu et al. 2021 [22].

## General information and DEGs and mutation rates

BRCA gene expression and clinical data were retrieved from The Cancer Genome Atlas (TCGA) [21], comprising 1,113 BRCA samples and 113 normal samples as of September 26, 2024. mRNA expression data were obtained from the Gene Expression Omnibus (GEO) under Series GSE41119 (Platform: GPL8269), which includes 287 BRCA cases (Table 1 and S1a). A curated list of fatty acid metabolism genes (FAMGs) was compiled [23] (Table S1b). In cases where multiple probes corresponded to a single gene, the arithmetic mean of these probe values was calculated to represent the gene's final expression level. The Sva and Limma [24] of R4.1.0 [25] were then employed exclusively for multi-chips data rectification (batch normalize). Subsequent to the standardization of the datasets, batch effect normalization was executed employing the SVA package. The efficacy of batch effect rectification was gauged through PCA (Principal Component Analysis). Differential expression analyses between BRCA and control groups were conducted utilizing the Linear Models for Microarray Data (limma) package. To ensure the accuracy of mRNA expression data, transcription data were matched and organized using Perl, and probe IDs were subsequently converted into gene names. Differential expression of FAMGs was identified based on a false discovery rate (FDR) < 0.05 and an absolute log2 fold change (|log2 FC|) ≥ 1. The significance of differentially expressed FAMGs (DEGs) was further evaluated, and their mutation frequencies were estimated using cBioPortal.

**Table 1:** Patients'clinical features

TCGA		GEO	
Variables	Number of samples	Variables	Number of samples
Gender		Gender	
Male/Female	12/1085	Male/Female	Unknown
Age at diagnosis		Age at diagnosis	
≤ 65/> 65	776/321	≤ 65/> 65	108/31
Grade		Grade	
G1/G2/G3/G4/NA	Unknown	G1/G2/G3/NA	14/37/97/10
Stage		Stage	
I/II/III/IV/NA	183/621/249/20/24	I/II/III/IV/NA	48/74/25/8/3
T		T	
T1/T2/T3/T4/NA	281/635/138/40/3	T1/T2/T3/T4	Unknown
M		M	
M0/M1/NA	912/22/163	M0/M1/NA	Unknown
N		N	
N0/N1/N2/N3/NA	291/364/120/74/20	N0/N1/N2/N3	Unknown

## Tumour classification and Cluster DEGs

Cluster analysis was conducted using the Limma and ConsensusClusterPlus packages, identifying two distinct prognosis-related FAMG clusters: Cluster 1 and Cluster 2. The Survminer package was employed to evaluate the survival outcomes associated with FAMGs, while the survival package was used to assess the predictive value of FAMGs. Differential expression analysis of specific genes between subtypes and tissue types was performed using Limma. After excluding normal samples, FAMG expression data were matched to survival time using the Limma, survival, and ConsensusClusterPlus packages. The consolidated data were subsequently subjected to univariate Cox analysis. Gene expression was then clustered to determine the optimal number of clusters for stratification. DEGs within FAMG clusters were identified using a false discovery rate (FDR) < 0.05 and an absolute log2 fold change (|log2 FC|) ≥ 1. The expression patterns of these genes were visualized using a heatmap.

## FAMG Prognostic Signature

DEGs were categorized into low-risk and high-risk groups based on Lasso regression analysis, which identified two distinct risk patterns. Kaplan–Meier survival curves were constructed to compare the survival outcomes between these two groups. To evaluate the model's predictive accuracy for BRCA survival, time-dependent receiver operating characteristic (ROC) curves were generated using the timeROC package. The relationship between FAMG expression levels, survival status, and the calculated risk score was assessed through a hazard curve analysis. Independent prognostic analysis was conducted to confirm that the predictive model was influenced by clinical variables. Additionally, an association between two FAMGs was identified, supporting the interaction between risk and clinical parameters. Further validation was performed using t-distributed stochastic neighbor embedding (t-SNE) and principal component analysis (PCA) to confirm that the prognostic model effectively stratified patients into distinct risk groups. A nomogram was subsequently constructed to estimate the 1-, 3-, and 5-year overall survival (OS) probabilities for BRCA patients, providing a visual representation of the model's prognostic capacity.

## Functional Enrichment Analysis and GSEA analyses

To elucidate the biological functions and pathway involvements of the identified DEGs, Gene Ontology (GO) and Kyoto Encyclopedia of Genes and Genomes (KEGG) enrichment analyses were conducted. Using R, the functional impact of differentially expressed FAMGs was examined across three GO domains: biological processes (BP), molecular functions (MF), and cellular components (CC). This analysis aimed to uncover the broader biological themes and molecular pathways regulated by FAMGs, thereby offering mechanistic insights into their roles in BRCA progression. Furthermore, the KEGG analysis provided a detailed mapping of the metabolic and signaling pathways influenced by these genes, highlighting potential therapeutic targets and revealing the intricate molecular interplay underlying BRCA pathogenesis. GSEA was used to detect related functions and route alterations in a variety of samples. The accompanying score and diagrams were also utilized to determine the dynamic nature of the activities and paths within the various risk subcategories. Each sample was labeled'H'or'L'.

## The levels of immune activation in different segments

The assessment of ssGSEA was employed. We assessed the enriching values of immune cells and activities. We also examined the connection between FAMGs, checkpoints, and mRNA chemical modifications (m6 A, m1 A, M7G, and m5 C). We also identified m6 A, m1 A, M7G, and m5 C regulators [23] (Table S2).

## Mendelian Randomization Analysis

To ensure the independence of exposure and outcome variables in our genome-wide association study (GWAS) summary data, we conducted an association analysis using the TwoSampleMR package in R. We designated FAMG-related expression as the exposure and BRCA as the outcome to investigate potential causal relationships. The analysis comprised three key steps: (1)Instrumental Variable (IV) Selection: FAMG-related expressions were filtered using a significance threshold of *P* < 5 × 10⁻⁸ to identify strongly associated SNPs. (2) Independence Configuration: Linkage disequilibrium (LD) between SNPs was calculated using the PLINK clustering method, excluding SNPs with an LD coefficient (r^2^ > 0.001) within a 10,000 kb window to ensure SNP independence and minimize pleiotropic bias. (3) Statistical Strength Assessment: The robustness of instrumental variables was evaluated using the F-statistic (F = β^2^/SE^2^), with F < 10 indicating insufficient strength to mitigate confounding. Following SNP identification, the harmonise_data function within TwoSampleMR was employed to align allelic directions between exposure and outcome, excluding incompatible SNPs. Causal inference was performed using the inverse variance-weighted (IVW) method, which leverages the variance of instrumental variables as weights to estimate causal effects, thereby providing insights into the genetic architecture underlying BRCA susceptibility.

## Results

### Differentially expressed FAMGs

**Fig. 2:**
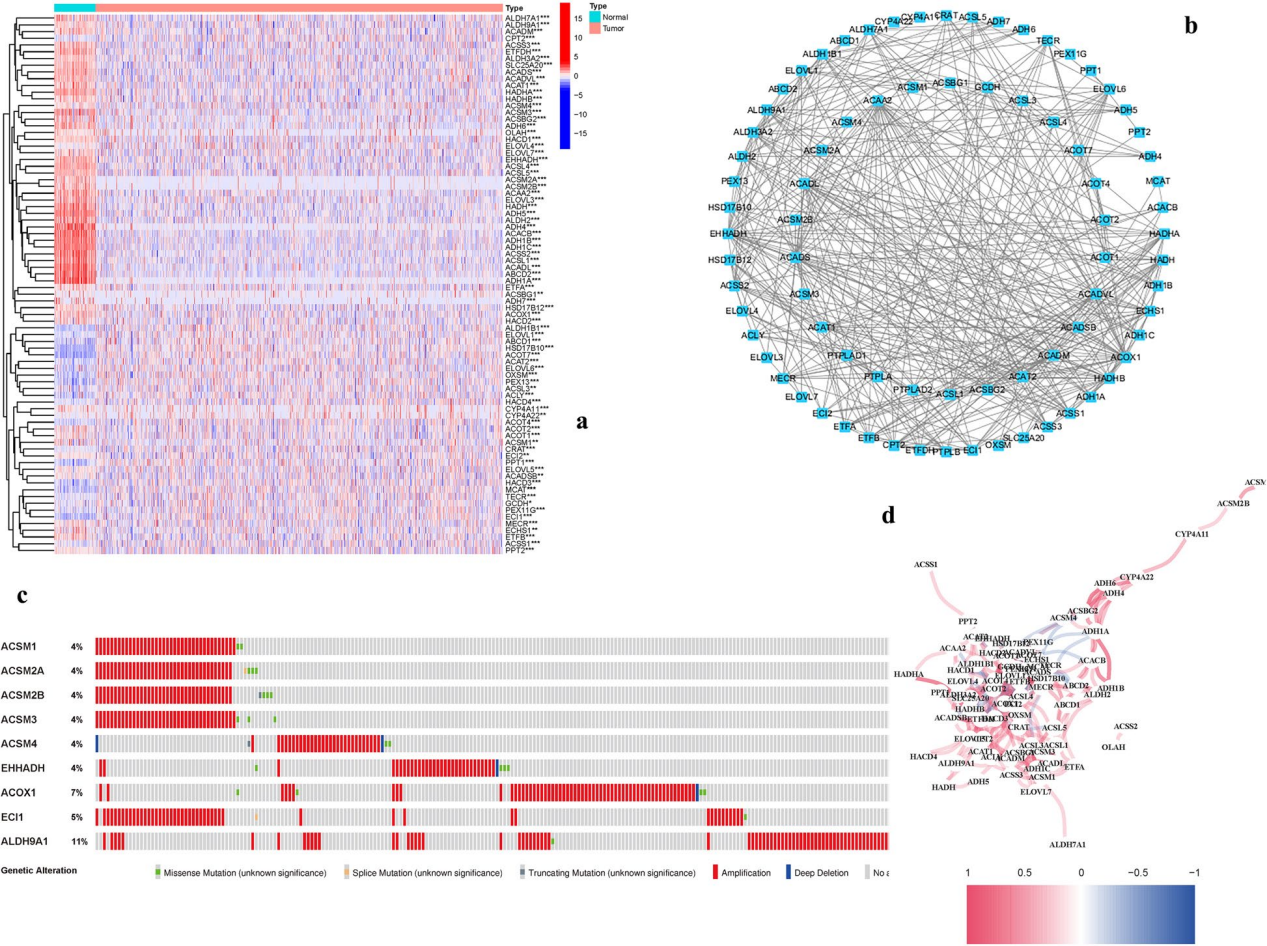
FAMGs and their interactions (**a**): Heatmap showing FAMGs expression levels in normal and malignant tissues. (**b**): PPI network. (**c**): The FAMGs'correlation network. (**d**). Variations in FAMGs

80 DEGs were discovered (27 up-, 53 down-; Table S2) compared to normal samples (Fig. 2a). Figure 2b presents the results of a PPI research that was conducted to further analyze the interactions of FAMGs. It was determined that ACADS, ACOX1, EHHADH, ACAA2, HADHB, ACADL, and ALDH3 A2 were hub genes (Table S4), which might be utilized to create a BRCA prognostic indicators. The correlation network, seen in Fig. 2c, comprises all FAMGs. The 2 most frequent forms of mutations were found to be truncating and missense mutations (Fig. 2d). ALDH9 A1 is the gene with the highest mutation rate (11%). The finding that ALDH9 A1 exhibits the highest mutation rate (11%) underscores its potential significance in disease pathogenesis. This elevated mutation frequency suggests that ALDH9 A1 may play a pivotal role in tumorigenesis, possibly influencing key metabolic or signaling pathways. Its high mutation rate highlights ALDH9 A1 as a candidate for further functional characterization and a potential biomarker for targeted therapeutic interventions.

## Alterations of regulatory FAMGs are associated with clinicopathological and molecular characteristics

**Fig. 3:**
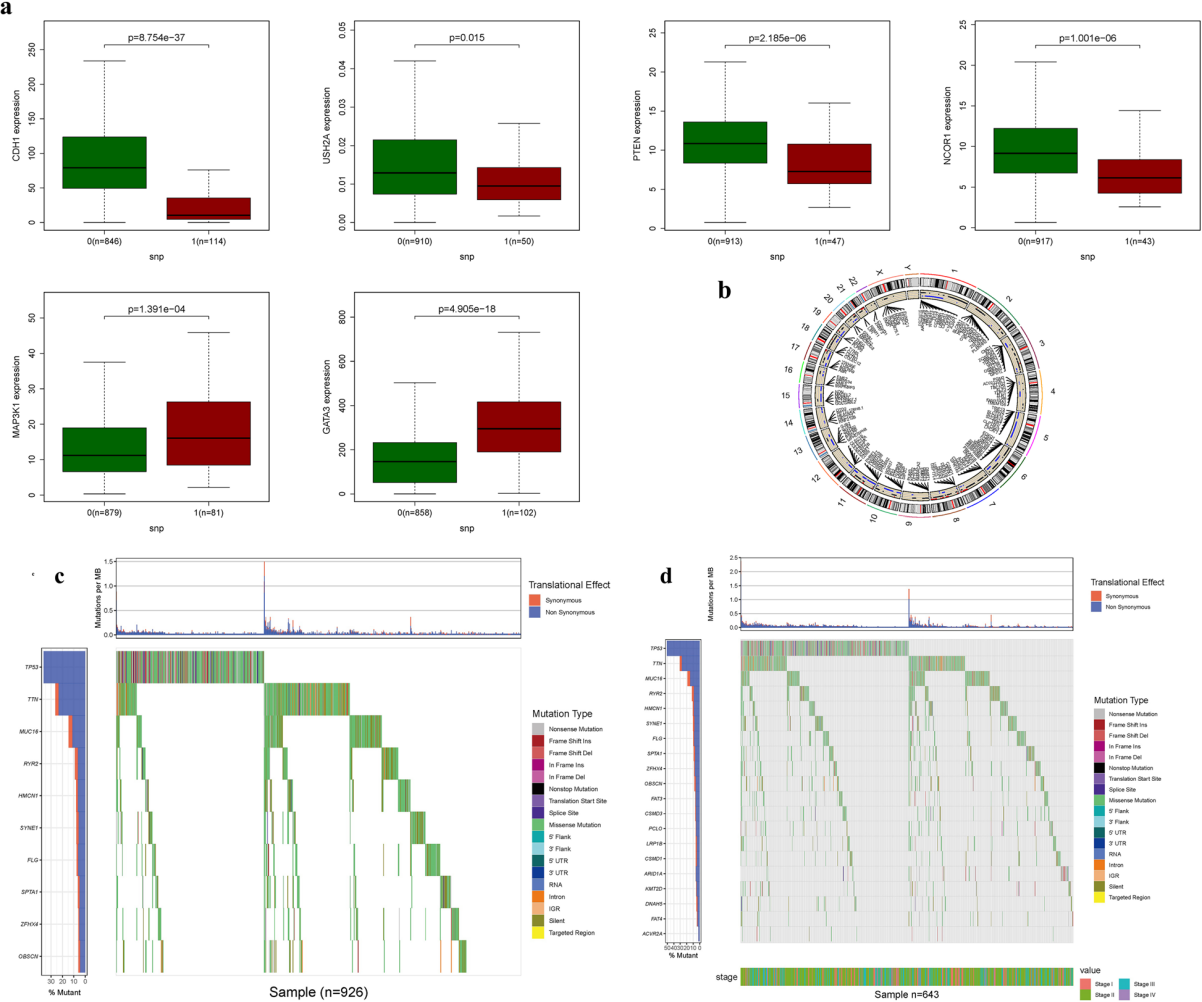
SNP and mutation analysis. (**a**) Prognostic signatures and SNP. (**b**) CNV analysis. (**c**) Waterfal. (**d**) Cilinical-waterfall

The association between alterations in fatty acid metabolism-regulating genes (including CNV, SNPs, and mutations) and clinicopathological characteristics in BRCA patients was investigated. A correlation analysis between DEGs in the prognostic model and SNPs revealed six SNP-driven DEGs: CDH1, GATA3, MAP3 K1, NCOR1, PTEN, and USH2 A (Fig. 3a). Further analysis of DEG expression and CNV identified numerous DEGs influenced by CNV (Fig. 3b). In particular, the expression of six genes was significantly upregulated in the mutation group compared to the non-mutation group (*P* < 0.05), suggesting that dysregulation of these critical genes may be driven by SNPs in BRCA. A waterfall plot was used to visualize the mutation status of these genes, showing that the mutation frequency of DEGs in the prognostic model ranged from 8 to 38% (Fig. 3c-d), indicating a potential association between BRCA mutations and the deregulation of key genes. The predictive capabilities of the model for drug response revealed notable differences in the expression of certain genes (Figure S1). Additionally, an analysis of the relationship between DEG expression and drug sensitivity in the prognostic model showed that several genes were strongly associated with drug sensitivity. Notably, CEL expression was strongly correlated with the sensitivity to Nelarabine, Fluphenazine, Dexamethasone Decadron, Digoxin, Idarubicin, Fludarabine, Hydroxyurea, and Dexrazoxane, suggesting potential therapeutic pathways (Figure S2).

## Tumour classification

**Fig. 4:**
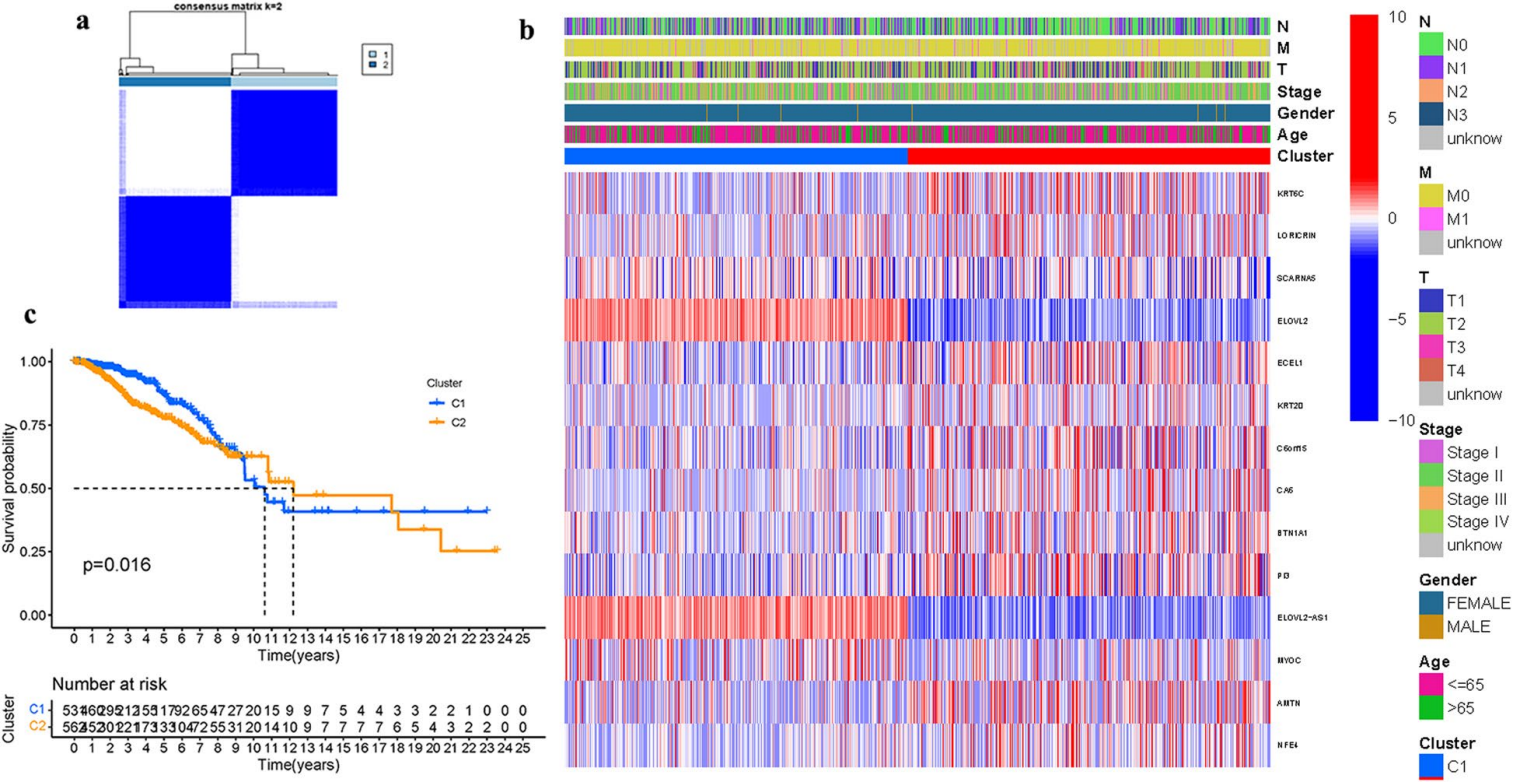
The FAMGs were used to classify tumors. (**a**): The consensus. (**b**). Heatmap. (**c**): Kaplan–Meier OS curves

To assess the associations between FAMGs and BRCA, a dominant clustering analysis was performed on 1113 BRCA individuals in the TCGA cohort. When k was 2, intraorganizational links were strongest and intergenerational connections were weakest (Fig. 4a). The gene expression patterns and clinical characteristics were displayed (Fig. 4b, Table.S5). To test the predictability of FAMGs, a survival investigation was performed, and cluster 1 had a greater chance of survival (Fig. 4c).

## Prognostic gene model of TCGA cohort

**Fig. 5:**
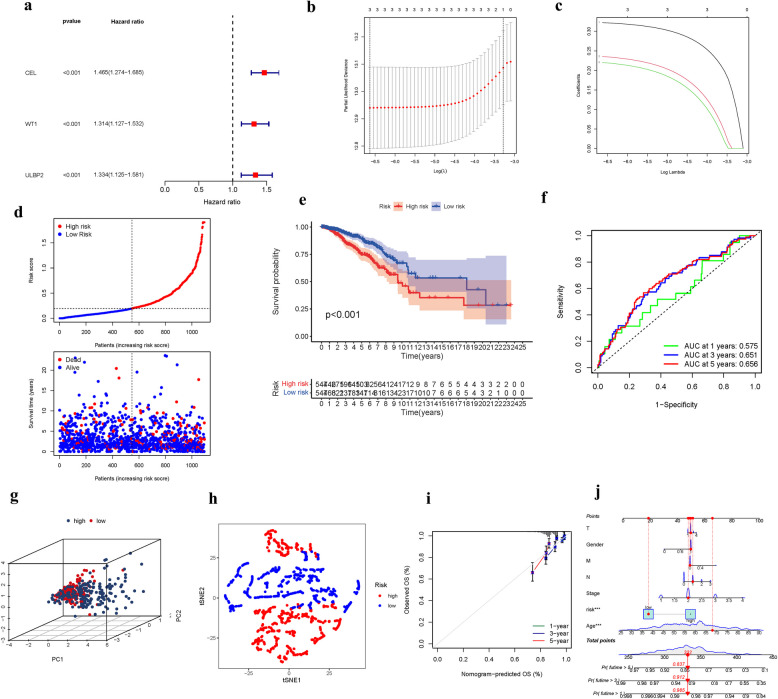
Risk signature of TCGA cohort. (**a**): Univariate cox regression analysis. (**b**): LASSO regression. (**c**): Cross-validation. (**d**): Survival status. (**e**): Kaplan–Meier curves. (**f**): The AUC of survival rate. (**g**): PCA. (**h**): t-SNE. (**i**-**j**) Nomogram

The univariate and multivariate COX analyses identified three FAMGs (CEL, WT1, and ULBP2) as independent prognostic indicators for BRCA (Fig. 5a). Based on these findings, a gene signature was developed (Fig. 5b-c). The risk score associated with this signature was negatively correlated with the survival outcomes of BRCA patients. Notably, the majority of the unexpected FAMGs in this study exhibited a significant adverse association with the evaluation model, suggesting the need for further investigation (Fig. 5d). High-risk FAMGs were strongly linked to a decreased probability of survival (Fig. 5e). The AUC for the FAMGs signature was 0.575, 0.651, and 0.656 for 1, 3, and 5-year overall survival (OS), respectively (Fig. 5f). Upon further analysis, it was observed that the survival time for patients who succumbed to the disease was typically over one year, which may explain the relatively low AUC value observed for the first year. Using PCA and t-SNE, BRCA patients were efficiently classified into two distinct risk categories (Fig. 5g-h). The hybrid nomogram, which integrated clinicopathological features from TCGA and the FAMGs prognostic signature, demonstrated both stability and accuracy, indicating its potential to enhance the therapeutic management of BRCA patients (Fig. 5i-j).

## Risk signature of GEO and risk model's independent prognostic value

**Fig. 6:**
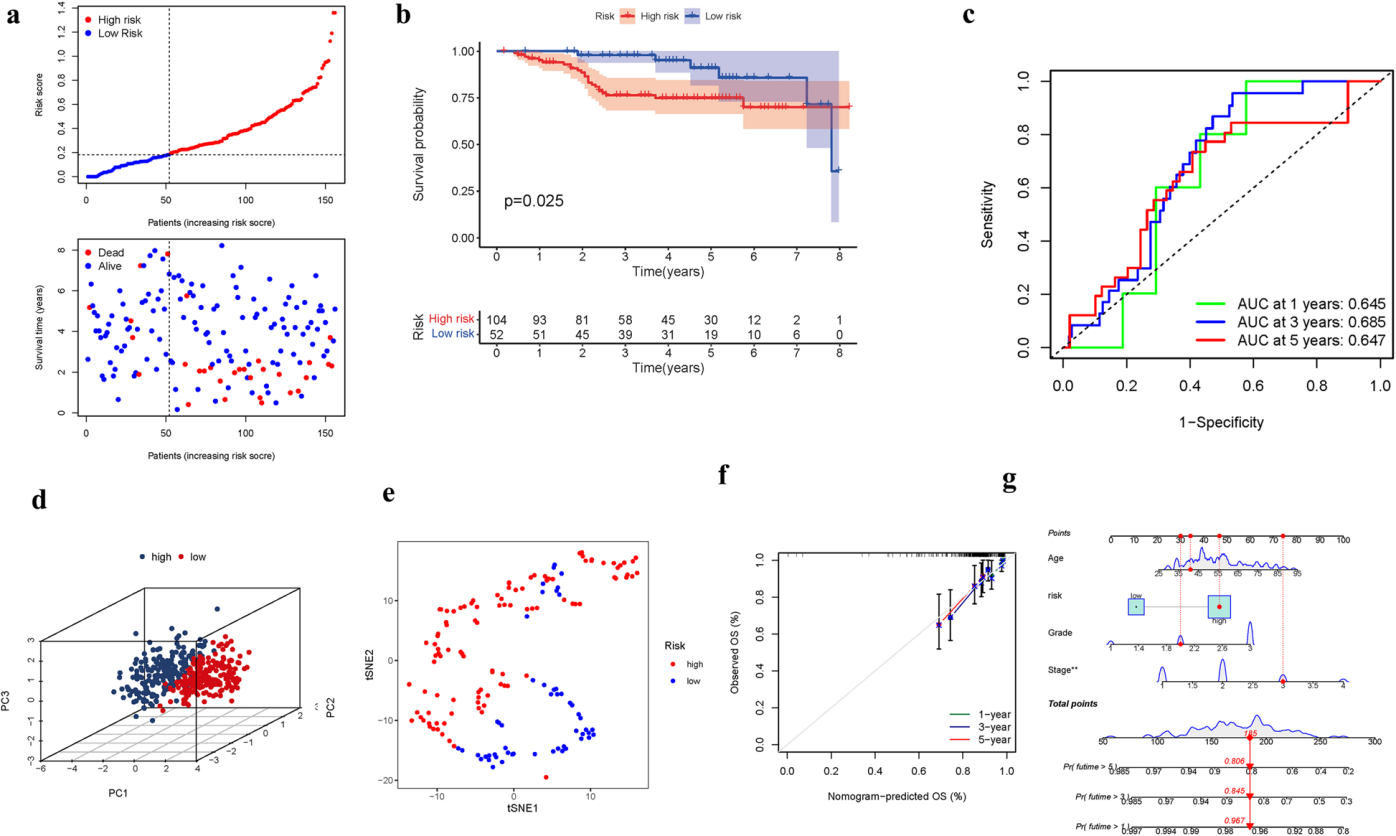
Risk model of GEO. (**a**): Survival status. (**b**): Kaplan–Meier curves. (**c**): The AUC of survival rate. (**d**): PCA. (**e**): t-SNE. (**f**-**g**) Nomogram

**Fig. 7:**
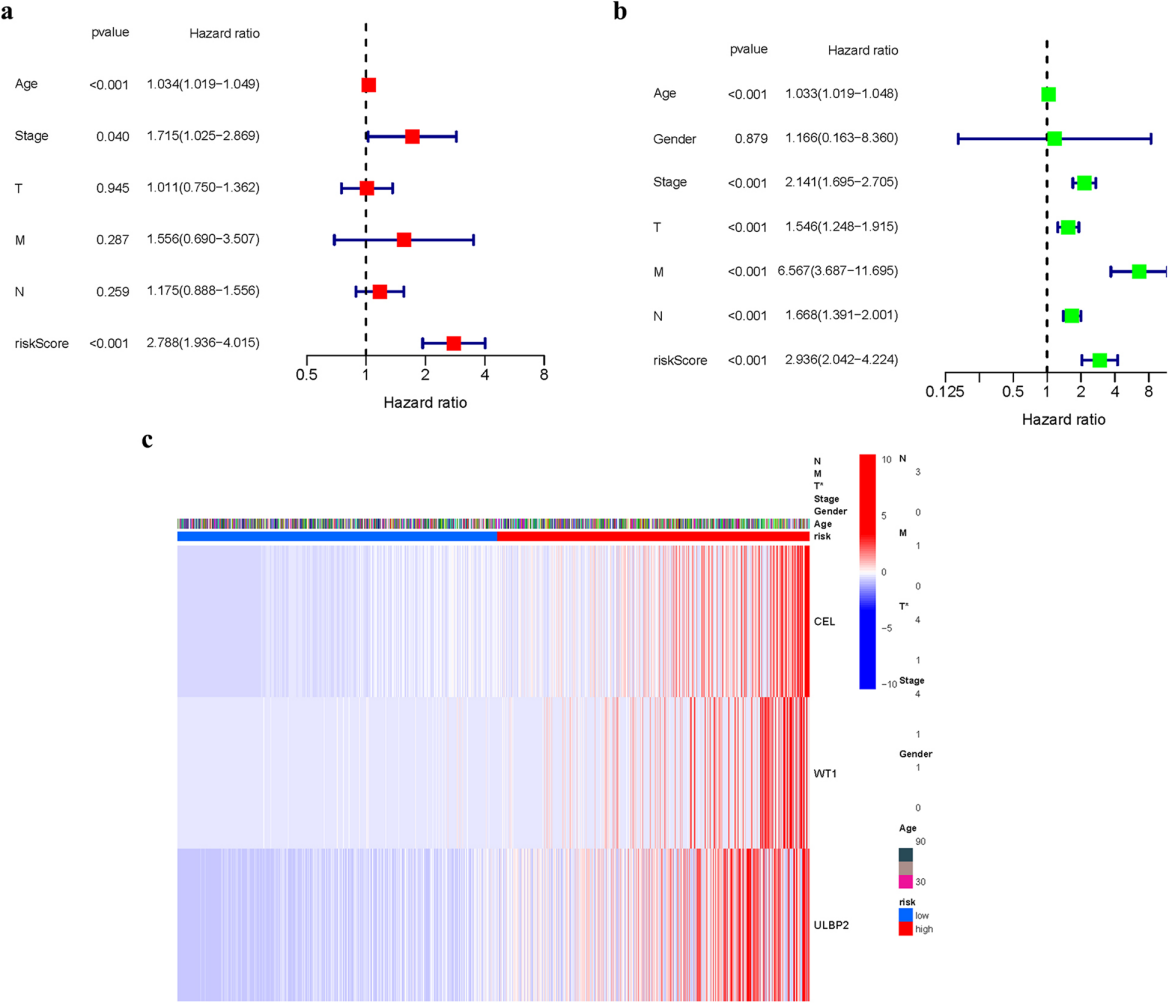
Cox regression analyses. (**a**): Multivariate. (**b**): Univariate. (**c**): Heatmap (*P < 0.05)

A GEO cohort of 287 BRCA individuals served as the verification group. The elevated expression of numerous FAMGs in the low-risk group and their greater prevalence among surviving patients suggests a potential protective or tumor-suppressive role for these genes. This pattern implies that FAMGs may contribute to enhanced cellular homeostasis, immune surveillance, or resistance to oncogenic stress in low-risk individuals. Further investigation into the functional mechanisms of these FAMGs could clarify their role in modulating cancer progression and improving patient outcomes (Fig. 6a). The presence of high-risk PRG signatures was associated with a reduced chance of survival (*P* = 0.025, Fig. 6b). For 1-, 3-, and 5-year survival rates, the AUC of the characteristic FAMGs signature was 0.645, 0.685, and 0.647, respectively (Figures.6c). We studied the data and determined that the majority of patients in the GEO data lived for more than three years, which may account for the difference between the AUC and the TCGA data. The PCA and t-SNE findings were displayed (Fig. 6d-e). The hybrid nomogram, including clinicopathological characteristics of GEO and the FAMGs's prognostic signature, was stable and accurate, which demonstrated great potential in the therapy of BRCA patients (Fig. 6f-g). The FAMGs signature (95% CI: 1.936–4.015), Age (HR: 1.034, 95 CI: 1.019–1.049), Stage (HR: 1.715, 95 CI: 1.025–2.869) were the major risk factors of BRCA individuals'OS (Fig. 7a-b). We also created a heatmap of clinical manifestations. (Fig. 7c) (Table.S6).

## Functional enrichment analysis and GSEA analyses

**Fig. 8:**
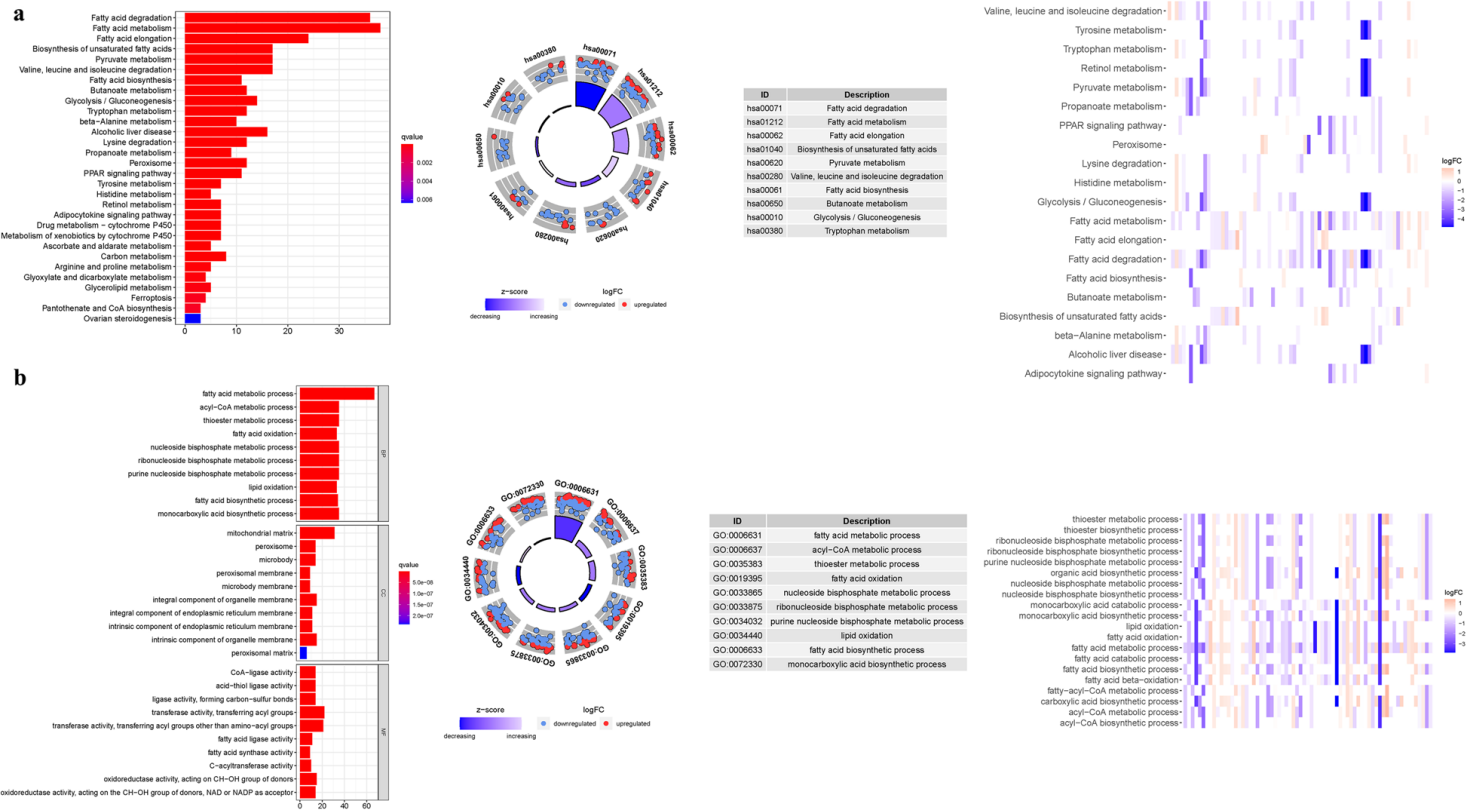
Enrichment analyses. (**a**): GO. (**b**): KEGG

**Fig. 9:**
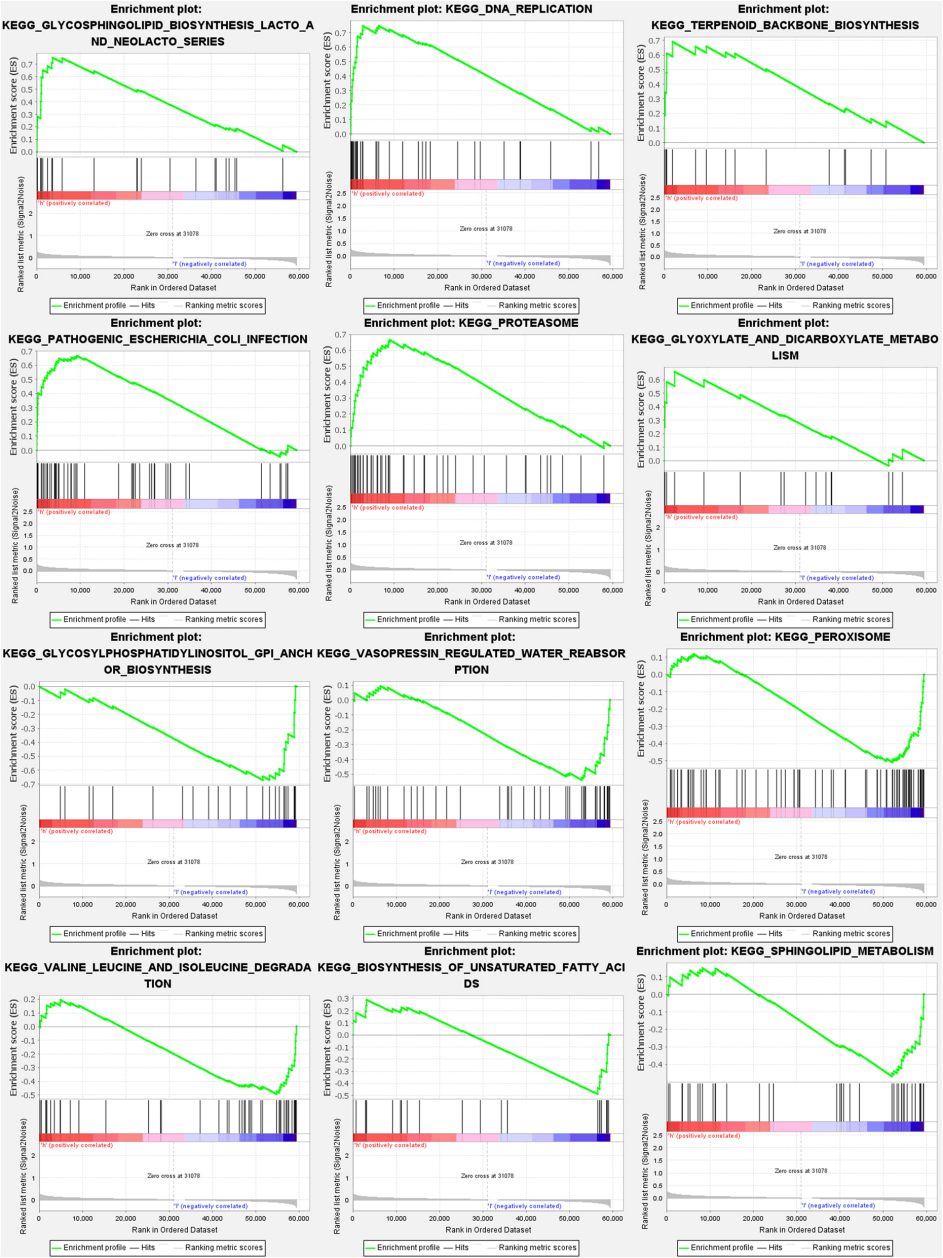
GSEA analyses for FAMGs

GO analysis revealed 635 core targets The MF mainly involvesATPase activity (GO:0016887), amide binding (GO:0033218), sulfur compound binding (GO:1,901,681). The CC mainly involves mitochondrial inner membrane (GO:0005743), mitochondrial matrix (GO:0005759), intrinsic component of organelle membrane (GO:0031300). The BP mainly involves lipid transport (GO:0006869), purine nucleotide metabolic process (GO:0006163). Furthermore, KEGG analysis was used to identify the primary signaling pathways, which indicated that FAMGs were mostly engaged in the Thermogenesis (hsa04714), Alcoholic liver disease (hsa04936), Carbon metabolism (hsa01200). (Fig. 8a-b and Table S7a-b). The results of GSEA reveal that the prognostic signatures of FAMGs predominantly influence immune-related and malignant cell pathways, including the Wnt, neurotrophin, chemokine, and calcium signaling pathways. These pathways are critical in regulating tumor progression, immune response, and cellular communication within the tumor microenvironment. Notably, the p53 signaling pathway emerged as one of the most enriched, suggesting a potential link between FAMGs and the regulation of cellular stress responses, genomic stability, and apoptosis. This implies that FAMGs may play a pivotal role in modulating both the immune landscape and malignant cell behaviors in cancer. Future studies should explore the mechanistic underpinnings of this association and its implications for therapeutic strategies targeting these pathways (Table S8a-b) (Fig. 9).

## Immune cells, function, and mRNA chemical modifications

**Fig. 10:**
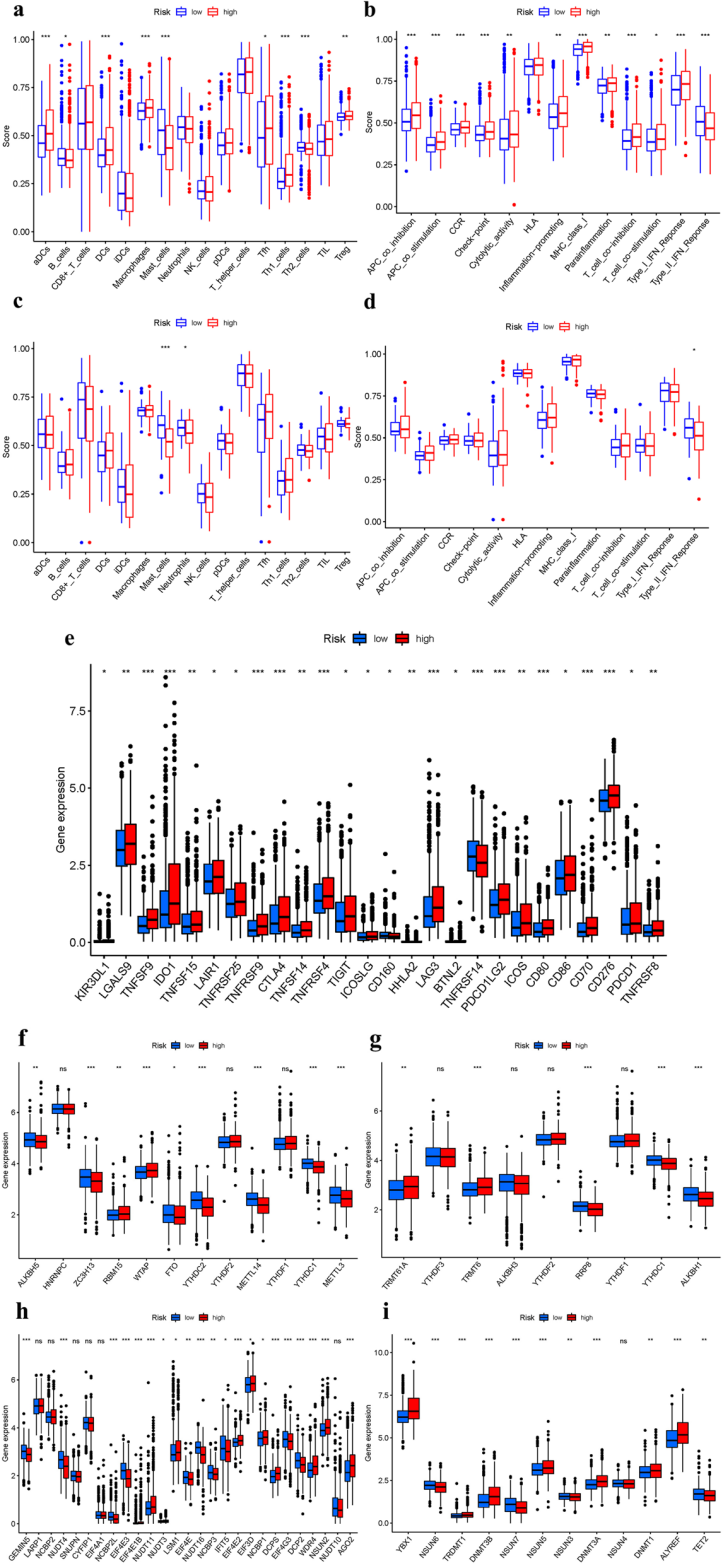
Immune cells and function mRNA chemical modifications. (a + b): TCGA. (c + d): GEO. (e) Immune checkpoint. (f) m6 A. (g) M1 A. (h) M7G. (i) M5 C

We investigated the enriched values of 16 immune cells and the 13 activities in both the 2 cohorts. In the TCGA, CD8 + _T_cells, iDCs, Neutrophils, NK_cells, pDCs, T_helper_cells, and TIL did not significantly differ between both the two categories (*P* > 0.05). In the high-risk grouping, other immune cells penetrate at a higher rate (Fig. 10a). The two groups did not vary significantly in HLA(P > 0.05). In the high-risk category, other immune-related processes are frequently more important (Fig. 10b). When the immunological status of the GEO cohort was evaluated, similar results were obtained (Fig. 10c-d). *TNFSF9, IDO1, TNFRSF9, CTLA4, TNFRSF4, LAG3, TNFRSF14, PDCD1LG2, CD80, CD70, and CD276* and other genes were expressed differently in the two risk cohorts (Fig. 10e). In m6a, When FAMGs expression was examined between the 2 risk groups, *ALKBH5, ZC3H13, FTO, YTHDC2, METTL14, YTHDC1, and METTL3* were substantially more significant in the low-risk group (Fig. 10f). In m1 A, *RRP8, YTHDC1, and ALKBH1* were substantially more significant in the low-risk group (Fig. 10g). In M7G, *GEMIN5, NUDT4, NCBP2L, EIF4E3, EIF4E, NUDT16, NCBP3, IFIT5, EIF4G3, and DCP2* were substantially more significant in the high-risk group (Fig. 10h). In m5 C, *NSUN6, NSUN7, NSUN3, and TET2* were substantially more significant in the high-risk group (Fig. 10i).

## Mendelian randomization analysis

**Fig. 11:**
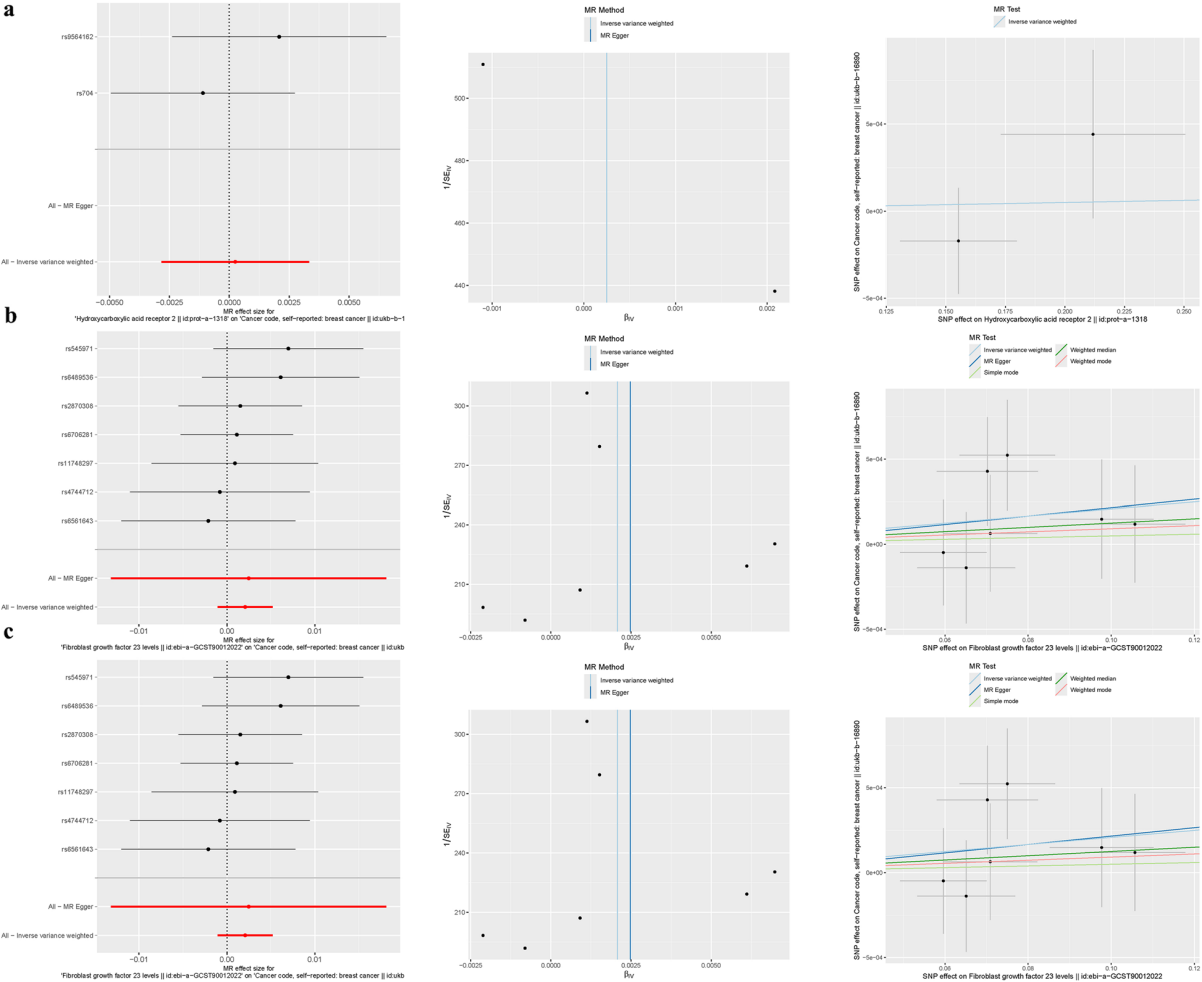
Mendelian Randomization Analysis. (**a**) CEL. (b) ULBP2. (**c**) WT1

In examining the direct linkage between the FAMGs (CEL, WT1, and ULBP2) and BRCA incidence, a forest plot was utilized for visual illustration, revealing a general symmetry in the data. Through sensitivity analysis employing the"leave-one-out"technique, it was determined that the omission of any individual SNP had a minimal effect on the results of the inverse variance-weighted (IVW) analysis, indicating that the remaining SNPs closely mirrored the overall dataset's findings. To further authenticate our outcomes, MR-Egger regression analysis was conducted, bolstering the integrity and reliability of our results and the chosen analytical framework (Fig. 11a-c).

## Discussion

Because of its late stage and poor prognosis, BRCA therapy is a serious clinical issue. Precision therapy for BRCA is currently constrained by a lack of tumor-killing initiators and specific tumor-targeting therapeutic medicines [26]. A recent study found that by changing the mechanism of programmed tumor cell death, the therapeutic impact of BRCA treatment may be enhanced [27]. As a result, it is critical to recognize and diagnose the sickness as soon as feasible. Reprogramming cellular metabolism is essential for tumor development. Cancer is distinguished by changes in cell metabolic activity [28]. One of the physiological indicators of human malignant tumors is increased glycolytic metabolism. Several studies have found that such as cysteine metabolism, nucleotide metabolism, and 2-hydroxyglutarate can be utilized to define and treat gliomas [29]. Increased anaerobic metabolic pathways are required in cancer stem-like cells for tumor formation, progression, and therapy resistance, according to BRCA. Although most research has concentrated on the impact of a particular fatty acid metabolism regulator in BRCA, the combined contributions of numerous fatty acid metabolism-related genes are unclear [30]. The examination of the role of distinct fatty acid metabolism patterns in BRCA progression may benefit in understanding it in BRCA progression, hence pointing to an effective therapeutic plan.

In BRCA, this research identified 80 DEGs associated with fatty acid metabolism. A Cox regression analysis conducted by our team revealed that FAMGs were strongly correlated with BRCA prognosis. Further investigation highlighted three prognostic FAMGs, which were differentially expressed between high-risk individuals. Notably, CEL, WT1, and ULBP2 were found to be highly expressed in the high-risk group, suggesting their potential involvement in BRCA oncogenesis. While the expression patterns of these genes offer promising avenues for future research, there remains insufficient evidence linking them to the regulation of specific transcription factors involved in pyroptosis, such as GPX4, SREBP, and ACSL4. This gap warrants further investigation to elucidate their potential roles in cancer progression. Celastrol (Cel), a naturally derived compound, exhibits notable anti-cancer properties and offers therapeutic potential for various ailments [31]. Bioinformatics analysis of 1,104 tumor cases by Yingnan Cui revealed that Cel was overexpressed in BRCA, with higher CEL expression correlating with lower survival rates, suggesting that CEL expression may serve as a distinct prognostic marker for BRCA. Survival multivariate analysis further corroborated this association [32]. In Julia H. Carter's study, quantitative chemical proteomics was employed to identify the anti-cancer targets of Celastrol in HCT116 human colon cancer cells. WT1 and p53 were found to be associated with ovarian tumor type, grade, FIGO stage, and patient survival. Specifically, WT1 expression was linked to advanced grade, FIGO stage, and poor survival outcomes, while uniform nuclear expression of p53 was associated with increased invasion. In the age-adjusted Cox model, WT1 remained significant, whereas p53 did not, indicating that WT1 functions with high tissue and cell specificity [33]. Ye Zhang's research further elucidates the relationship between WT1 expression and prognosis in BRCA patients, shedding light on the biological impacts and molecular mechanisms of WT1 in the initiation and progression of BRCA, including its role in cell proliferation, apoptosis, invasion, and metastasis [34]. These findings reinforce the validity and biological plausibility of our results, as the identified FAMGs appear to be closely associated with the oncogenic process in patients with BRCA. Insights from the GSE41119 dataset further support the potential utility of fatty acid metabolism-related traits as effective prognostic indicators, underscoring the clinical significance of metabolic reprogramming in cancer. Dysregulated fatty acid metabolism has been implicated in tumor progression, immune evasion, and therapeutic resistance, highlighting its pivotal role in shaping the tumor microenvironment and influencing disease dynamics. Integrating fatty acid metabolism-related traits into prognostic models could enhance risk stratification, inform personalized therapeutic strategies, and improve clinical outcomes by identifying patients who may benefit from targeted metabolic interventions.

Following that, KEGG analysis revealed that the genes were mostly involved in the Fatty acid degradation, Fatty acid metabolism, Fatty acid elongation, and PPAR signaling pathways. Peroxisome proliferator-activated receptor (PPAR) signaling and fatty acid degradation/metabolism pathways were drastically downregulated in ETV5-deficient hepatocytes, according to in vivo and in vitro RNA sequencing. ETV5 may bind to downstream genes'PPAR response element regions, enhancing their transactivity. Zhuo Mao discovered ETV5 as a novel transcription factor for controlling hepatic fatty acid metabolism, which is required for optimal -oxidation. ETV5 might be exploited as a therapeutic target to treat hepatic steatosis [35, 36]. As a result, fatty acid metabolism is crucial in BRCA. The most highly enriched pathway in GSEA was revealed to be the p53 signaling pathway. EZH2 increased STAT3 methylation and phosphorylation, as well as miR- 375 transcription activity, whereas miR- 375 directly targeted FOXO1. In cells and animals, either overexpression of EZH2 or downregulation of FOXO1 reduced anti-miR- 375 actions. FOXO1 has been identified as a p53 signaling pathway activator [37]. A high level of CDKL3 expression in BRCA tissues, which is associated with a poor prognosis in TNBC individuals CDKL3 knockdown reduces BRCA cell proliferation and migration, but CDKL3 overexpression has the reverse effect. In vitro, CDKL3 knockdown reliably causes cell death while reducing tumor formation in vivo [38]. FAMGs may influence BRCA cell migration and proliferation through the modulation of the p53 signaling pathway, as suggested by the aforementioned findings. Emerging evidence indicates that FAMGs could alter BRCA cell dynamics by affecting the functional integrity of p53, a key regulator of genomic stability, cell cycle progression, apoptosis, and senescence. Disruptions in fatty acid metabolism may impact p53 activity through the modulation of lipid-derived signaling molecules, oxidative stress levels, and membrane fluidity, thereby influencing downstream transcriptional targets involved in cell adhesion, motility, and proliferation. This mechanistic link underscores the potential role of FAMGs in driving tumor progression via metabolic reprogramming and p53 pathway dysregulation, offering novel insights into the metastatic potential of BRCA and identifying possible targets for therapeutic intervention.

The findings presented here hold significant potential for application in various therapeutic contexts. FAMGs emerge as promising markers for predicting BRCA patient outcomes. To date, 172 distinct types of RNA alterations have been identified, with the most prevalent chemical modifications being m6 A, m1 A, and m5 C [19]. Among these, m6 A stands out as one of the most common eukaryotic mRNA modifications. This modification involves the methylation of the sixth nitrogen atom of adenosine, with S-adenosylmethionine serving as the methyl donor through the action of methyltransferases [39]. Both m6 A and post-translational histone modifications have been implicated in the epigenetic regulation of cell growth and differentiation [19]. In our study, we also examined the effects of various mRNA chemical modifications on FAMGs and observed that different modifications exerted distinct influences on gene expression. Notably, we identified differential expression of YTHDF1 across various groups, suggesting that this gene could serve as a valuable biomarker in the future for BRCA drug development and protein modeling. Recent studies have highlighted the relationship between several forms of cell death mechanisms and anticancer immunity. Even in tumors resistant to immune checkpoint inhibitors (ICIs), inducing pyroptosis, ferroptosis, and necroptosis with ICIs has shown to synergistically enhance anticancer efficacy [40, 41]. In ICI-resistant cancers, de novo pyroptosis can create an inflammatory microenvironment that enhances tumor sensitivity to ICIs, thereby promoting pyroptosis and inhibiting tumor progression [42]. Based on these observations, it is plausible to hypothesize that modifications to FAMGs are closely linked to the initiation and progression of BRCA, potentially offering new avenues for therapeutic intervention.

Our study has several limitations. First, we were unable to gather sufficiently diverse data from other public resources to validate the model's robustness and generalizability. Additionally, the early expression analysis was limited to the three risk-associated FAMGs identified in the signature, which may restrict the breadth of the findings. This study utilized data from TCGA, which, despite its comprehensive scope and high data quality, introduces potential sources of bias. TCGA cohorts are predominantly composed of samples from Western populations, which may limit the applicability of the findings to other ethnic or genetic backgrounds. Furthermore, as TCGA samples are primarily obtained from surgical resections, advanced or metastatic cases may be underrepresented, thereby skewing the molecular profiles toward early-stage disease. Technical biases arising from sample preparation, sequencing platforms, and data processing pipelines may further affect data consistency and comparability. Independent validation in more diverse cohorts and complementary datasets will be crucial to enhance the robustness and clinical relevance of the findings.

## Conclusions

Finally, these findings enhance our understanding of the immune system's involvement in fatty acid metabolism, potentially paving the way for the development of novel therapeutic agents and predictive biomarkers. The results provide valuable insights into FAMGs implicated in BRCA progression, offering a deeper understanding of their potential roles in the initiation and advancement of BRCA carcinoma.

## Supplementary Information

Supplementary Material 1.

## Data Availability

The datasets generated and/or analysed during the current study are available in the [GEO] repository, [https://www.ncbi.nlm.nih.gov/geo/] (GSE41119); [TCGA] repository, [https://portal.gdc.cancer.gov/].
