# Supplementary material for: Identification of fatty acid metabolism-related genes in the tumor microenvironment of breast cancer by a development and validation of prognostic index signature
Source: Hereditas. 2025 Apr 7;162:55. doi: 10.1186/s41065-025-00425-4 (PMC11974137; doi:10.1186/s41065-025-00425-4)
Supplement: Supplementary file 1 — Supplementary Material 1. [file 41065_2025_425_MOESM1_ESM.doc]

Identification of Fatty Acid Metabolism-Related Genes in the tumor microenvironment of Breast Cancer by A Development and Validation of Prognostic Index Signature

**Supplementary appendix to the manuscript**

Contents of supplementary appendix

[Appendix 1 3](#__RefHeading___Toc1917)

[Datasets and fatty acid metabolism 3](#__RefHeading___Toc16847)

[Table 1b. fatty acid metabolism genes 4](#__RefHeading___Toc24742)

[Appendix 2 5](#__RefHeading___Toc17776)

[Table 2a. m6A genes. 5](#__RefHeading___Toc4885)

[Table 2b. m1A genes. 5](#__RefHeading___Toc13902)

[Table 2c. m7G genes. 5](#__RefHeading___Toc26520)

[Table 2d. m5C genes. 6](#__RefHeading___Toc1164)

[Appendix 3 7](#__RefHeading___Toc22056)

[DEGs linked to FAMGs 7](#__RefHeading___Toc30487)

[Table 3. 80 DEGs linked to FAMGs. 7](#__RefHeading___Toc25971)

[Appendix 4 10](#__RefHeading___Toc12177)

[The drug prediction of the model 10](#__RefHeading___Toc14230)

[Appendix 5 11](#__RefHeading___Toc15005)

[Correlation analysis of gene expression in prognostic signatures and drug sensitivity 12](#__RefHeading___Toc13748)

[Appendix 6 13](#__RefHeading___Toc30617)

[hub genes analysis 13](#__RefHeading___Toc6488)

[Table 4. Hub genes. 13](#__RefHeading___Toc15108)

[Appendix 7 16](#__RefHeading___Toc18874)

[The gene expression profile and clinical characteristics 16](#__RefHeading___Toc26906)

[Table 5. The gene expression profile and clinical characteristics. 16](#__RefHeading___Toc29796)

[Appendix 8 35](#__RefHeading___Toc29306)

[3 risk PRGs 35](#__RefHeading___Toc17354)

[Table 6. 3 risk PRGs. 35](#__RefHeading___Toc10959)

[Appendix 9 56](#__RefHeading___Toc27600)

[GO and KEGG enrichment analysis 56](#__RefHeading___Toc29130)

[Table 7a. GO enrichment analysis. 56](#__RefHeading___Toc17238)

[Table 7b. KEGG enrichment analysis. 79](#__RefHeading___Toc10486)

[Appendix 10 82](#__RefHeading___Toc6971)

[gene set enrichment analyses (GSEA) 82](#__RefHeading___Toc1326)

[Table 8a. GSEA of high rish. 82](#__RefHeading___Toc18047)

[Table 8b. GSEA of low rish. 94](#__RefHeading___Toc188)

# Appendix 1

**Datasets and fatty acid metabolism**

Table 1a. Patients' clinical features.

| TCGA | | GEO | |
| --- | --- | --- | --- |
| Variables | Number of samples | Variables | Number of samples |
| Gender |  | Gender |  |
| Male/Female | 12/1085 | Male/Female | Unknown |
| Age at diagnosis |  | Age at diagnosis |  |
| ≤65/>65 | 776/321 | ≤65/>65 | 108/31 |
| Grade |  | Grade |  |
| G1/G2/G3/G4/NA | Unknown | G1/G2/G3/NA | 14/37/97/10 |
| Stage |  | Stage |  |
| I/II/III/IV/NA | 183/621/249/20/24 | I/II/III/IV/NA | 48/74/25/8/3 |
| T |  | T |  |
| T1/T2/T3/T4/NA | 281/635/138/40/3 | T1/T2/T3/T4 | Unknown |
| M |  | M |  |
| M0/M1/NA | 912/22/163 | M0/M1/NA | Unknown |
| N |  | N |  |
| N0/N1/N2/N3/NA | 291/364/120/74/20 | N0/N1/N2/N3 | Unknown |

**Table 1b. fatty acid metabolism genes**

| ABCD1 | ACSBG1 | CPT1B | MCAT | ACSM4 |
| --- | --- | --- | --- | --- |
| ABCD2 | ACSBG2 | CPT2 | OLAH | ACSS1 |
| ACAA2 | CPT1C | CRAT | OXSM | ACSS2 |
| ACAD9 | ECI1 | CROT | MECR | ACSS3 |
| ACADL | ECI2 | ECHS1 | PPT1 | CPT1A |
| ACADM | CYP4A11 | ETFA | PPT2 | ALDH9A1 |
| ACADS | CYP4A22 | ETFB | ELOVL1 | ACACA |
| ACADVL | ADH1A | ETFDH | ELOVL2 | ACACB |
| ACAT1 | ADH1B | HADH | ELOVL3 | ACLY |
| ACSL1 | ADH1C | HADHA | ELOVL4 | FASN |
| ACSL3 | ADH7 | HADHB | ELOVL5 | EHHADH |
| ACSL4 | ADH4 | HSD17B10 | ELOVL6 | ACOX3 |
| ACSL5 | ADH5 | PEX11G | ELOVL7 | ACOX1 |
| ACSL6 | ADH6 | PEX13 | HSD17B12 | ACADSB |
| ACSM1 | ALDH2 | PEX14 | HACD2 | GCDH |
| ACSM2A | ALDH3A2 | SLC25A20 | HACD1 | TECR |
| ACSM2B | ALDH1B1 | ACAT2 | HACD4 | ACOT4 |
| ACSM3 | ALDH7A1 | ACAA1 | HACD3 | ACOT2 |
| ACOT1 | ACOT7 |  |  |  |

# Appendix 2

**mRNA chemical modifications**

**Table 2a. m6A genes.**

| METTL3 | YTHDC1 | ZC3H13 | RBM15 | HNRNPC |
| --- | --- | --- | --- | --- |
| METTL14 | YTHDC2 | FTO | YTHDF2 | KIAA1429 |
| WTAP | YTHDF1 | ALKBH5 |  |  |

**Table 2b. m1A genes.**

| YTHDF2 | YTHDF1 | TRMT61A | YTHDC1 | YTHDF3 |
| --- | --- | --- | --- | --- |
| RRP8 | ALKBH1 | ALKBH3 | TRMT6 |  |

**Table 2c. m7G genes.**

| METTL1 | EIF4E | EIF4A1 | NUDT4 | NCBP1 |
| --- | --- | --- | --- | --- |
| WDR4 | EIF4E1B | EIF4G3 | NUDT48 | NCBP2 |
| NSUN2 | EIF4E2 | IFIT5 | AGO2 | NCBP3 |
| DCP2 | EIF4E3 | LSM1 | CYFIP1 | EIF3D |
| DCPS | GEMIN5 | NCBP2L | NUDT16 | NUDT11 |
| NUDT10 | LARP1 | SNUPN | NUDT3 |  |

**Table 2d. m5C genes.**

| NSUN1 | DNMT2 | NSUN7 | TET2 | NSUN4 |
| --- | --- | --- | --- | --- |
| NSUN | DNMT3A | ALYREF | TRDMT1 | NSUN5 |
| NSUN3 | DNMT3B | DNMT1 | YBX1 | NSUN6 |

# Appendix 3

## **DEGs linked to FAMGs**

**Table 3. 80 DEGs linked to FAMGs.**

| gene | conMean | treatMean | logFC | pValue |
| --- | --- | --- | --- | --- |
| ABCD1 | 4.412543363 | 6.89388832 | 0.643707471 | 8.23E-11 |
| ABCD2 | 3.756637168 | 0.259905301 | -3.853383817 | 5.84E-49 |
| ACAA2 | 18.09509027 | 8.829421024 | -1.035207561 | 6.09E-39 |
| ACADL | 2.348556637 | 0.254324169 | -3.20703391 | 5.61E-55 |
| ACADM | 17.53000088 | 11.58477682 | -0.597595818 | 8.89E-22 |
| ACADS | 17.97521062 | 7.772136927 | -1.209625452 | 1.39E-33 |
| ACADVL | 69.6505354 | 44.64019066 | -0.641791254 | 8.16E-30 |
| ACAT1 | 12.56532743 | 7.745940431 | -0.697935954 | 4.60E-21 |
| ACSL1 | 119.6340858 | 19.83063971 | -2.592825374 | 4.97E-42 |
| ACSL3 | 28.16738761 | 29.20262956 | 0.052072516 | 0.002231712 |
| ACSL4 | 13.90748673 | 6.360148967 | -1.128729267 | 4.06E-42 |
| ACSL5 | 8.971825664 | 4.908933333 | -0.869992013 | 1.81E-33 |
| ACSM1 | 10.57089558 | 3.724557233 | -1.504956772 | 0.005505216 |
| ACSM2A | 0.027207965 | 0.008207727 | -1.728974407 | 1.27E-48 |
| ACSM2B | 0.027669912 | 0.007416173 | -1.899571322 | 1.01E-50 |
| ACSM3 | 1.838834513 | 0.532844385 | -1.787005484 | 1.76E-26 |
| ACSM4 | 0.172543363 | 0.149496226 | -0.206849911 | 2.42E-11 |
| ACSS1 | 5.15669292 | 7.442733423 | 0.529386428 | 0.000139582 |
| ACSS2 | 26.74942035 | 9.088473495 | -1.557397725 | 1.28E-56 |
| ACSS3 | 2.983447788 | 1.386837736 | -1.105181531 | 2.82E-24 |
| CPT2 | 1.212872566 | 1.399504852 | 0.206488511 | 0.000107854 |
| CRAT | 16.82849115 | 34.04089443 | 1.016363115 | 3.62E-05 |
| ECHS1 | 139.2637814 | 112.5026755 | -0.307860791 | 0.008478148 |
| ETFA | 19.51750177 | 15.57118778 | -0.325889402 | 1.01E-11 |
| ETFB | 2.558504425 | 1.816242318 | -0.494344033 | 4.19E-13 |
| ETFDH | 8.882746903 | 5.272091824 | -0.752630387 | 6.57E-33 |
| HADH | 15.45057788 | 6.872126415 | -1.168832318 | 8.88E-52 |
| HADHA | 81.56960442 | 56.41957062 | -0.531835969 | 3.95E-46 |
| HADHB | 43.68538496 | 35.51479704 | -0.298730463 | 2.48E-16 |
| HSD17B10 | 44.14703982 | 80.74455544 | 0.871048278 | 1.27E-38 |
| PEX11G | 2.727040708 | 4.276894429 | 0.64922736 | 3.26E-06 |
| PEX13 | 6.615292035 | 9.182866577 | 0.473139738 | 1.33E-18 |
| SLC25A20 | 16.3681823 | 10.4754619 | -0.643880259 | 3.60E-30 |
| ACAT2 | 5.052263717 | 7.781073765 | 0.62303931 | 6.15E-12 |
| EHHADH | 4.73739292 | 2.45350018 | -0.949251957 | 4.13E-34 |
| ACOX1 | 14.12772389 | 10.26981258 | -0.4601192 | 5.92E-16 |
| ACADSB | 19.80646903 | 20.08824205 | 0.020379609 | 0.003356846 |
| GCDH | 6.270272566 | 7.250180323 | 0.20948872 | 0.023528819 |
| ACSBG1 | 0.049218584 | 0.061406469 | 0.319187493 | 0.002715046 |
| ACSBG2 | 0.141306195 | 0.085798742 | -0.719796311 | 9.18E-16 |
| ECI1 | 10.05538584 | 19.49998562 | 0.955504619 | 1.47E-24 |
| ECI2 | 6.994947788 | 6.61777053 | -0.079968021 | 0.001582187 |
| CYP4A11 | 0.158223894 | 0.088739892 | -0.834312776 | 1.11E-16 |
| CYP4A22 | 0.048638938 | 0.089303953 | 0.876612311 | 0.002661245 |
| ADH1A | 0.958518584 | 0.041695508 | -4.522842545 | 1.83E-60 |
| ADH1B | 118.0069159 | 4.066921384 | -4.8587905 | 2.01E-58 |
| ADH1C | 9.831904425 | 1.001969632 | -3.294632108 | 2.14E-61 |
| ADH7 | 0.185023894 | 0.113770979 | -0.701578989 | 5.59E-19 |
| ADH4 | 0.761579646 | 0.059746361 | -3.672072167 | 2.67E-55 |
| ADH5 | 38.79688142 | 21.50176379 | -0.851485681 | 3.80E-58 |
| ADH6 | 0.12500885 | 0.055167206 | -1.180147415 | 3.45E-37 |
| ALDH2 | 36.97853009 | 8.75648221 | -2.078264569 | 2.02E-36 |
| ALDH3A2 | 9.752375221 | 6.67067637 | -0.547920584 | 6.46E-24 |
| ALDH1B1 | 9.834236283 | 16.4787478 | 0.744721694 | 1.20E-25 |
| ALDH7A1 | 7.523378761 | 5.074665049 | -0.568068123 | 9.52E-16 |
| ALDH9A1 | 52.87706903 | 43.3295664 | -0.287290411 | 1.15E-11 |
| ACACB | 25.00217965 | 2.328793172 | -3.424399453 | 5.88E-59 |
| ACLY | 33.82025664 | 44.46106703 | 0.394654965 | 1.63E-14 |
| MCAT | 5.805055752 | 8.294222372 | 0.514796804 | 9.91E-17 |
| OLAH | 0.233693805 | 0.315457502 | 0.432826169 | 1.80E-32 |
| OXSM | 4.050989381 | 5.312791644 | 0.391195832 | 2.83E-17 |
| MECR | 5.960052212 | 5.260156065 | -0.180219366 | 8.89E-08 |
| PPT1 | 61.52074513 | 99.31163558 | 0.69088978 | 1.18E-18 |
| PPT2 | 2.999415044 | 2.777868464 | -0.110702882 | 3.02E-07 |
| ELOVL1 | 31.74060531 | 40.19290791 | 0.340611317 | 1.75E-10 |
| ELOVL3 | 2.038204425 | 0.646446631 | -1.656695582 | 5.21E-25 |
| ELOVL4 | 0.824548673 | 1.138298023 | 0.465201763 | 1.44E-05 |
| ELOVL5 | 109.2631363 | 107.7458759 | -0.02017409 | 0.000322283 |
| ELOVL6 | 3.807786726 | 3.391463163 | -0.167044853 | 6.97E-07 |
| ELOVL7 | 4.761129204 | 2.639038994 | -0.851291112 | 1.18E-13 |
| HSD17B12 | 6.209530088 | 4.808743666 | -0.368824071 | 4.05E-17 |
| HACD2 | 26.84103363 | 19.76318805 | -0.441624539 | 1.08E-10 |
| HACD1 | 1.229454867 | 0.908473046 | -0.436503159 | 1.35E-23 |
| HACD4 | 1.689600885 | 2.868169542 | 0.763447811 | 1.13E-05 |
| HACD3 | 22.62532566 | 36.982669 | 0.708910788 | 5.65E-17 |
| TECR | 18.24874956 | 23.15418553 | 0.343475399 | 3.77E-06 |
| ACOT4 | 3.279846903 | 6.359256783 | 0.955229691 | 1.70E-14 |
| ACOT2 | 12.53480619 | 9.57258823 | -0.388958733 | 4.70E-05 |
| ACOT1 | 4.473786726 | 3.63782327 | -0.298421025 | 6.21E-05 |
| ACOT7 | 3.7761 | 9.880812219 | 1.387732667 | 2.54E-40 |

# Appendix 4

**The drug prediction of the model**


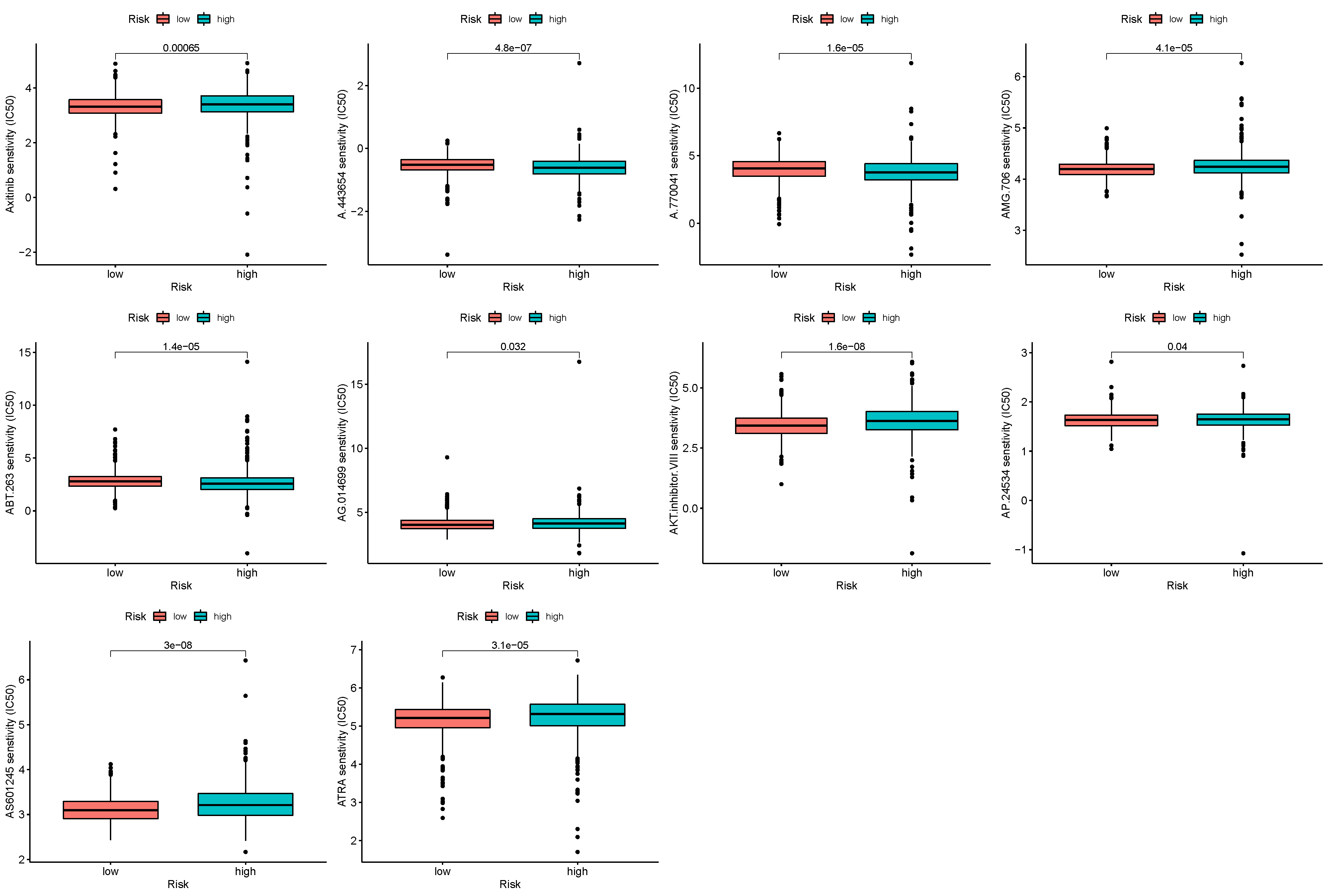


Figure S1. The drug prediction of the model.

# Appendix 5

## **Correlation analysis of gene expression in prognostic signatures and drug sensitivity**


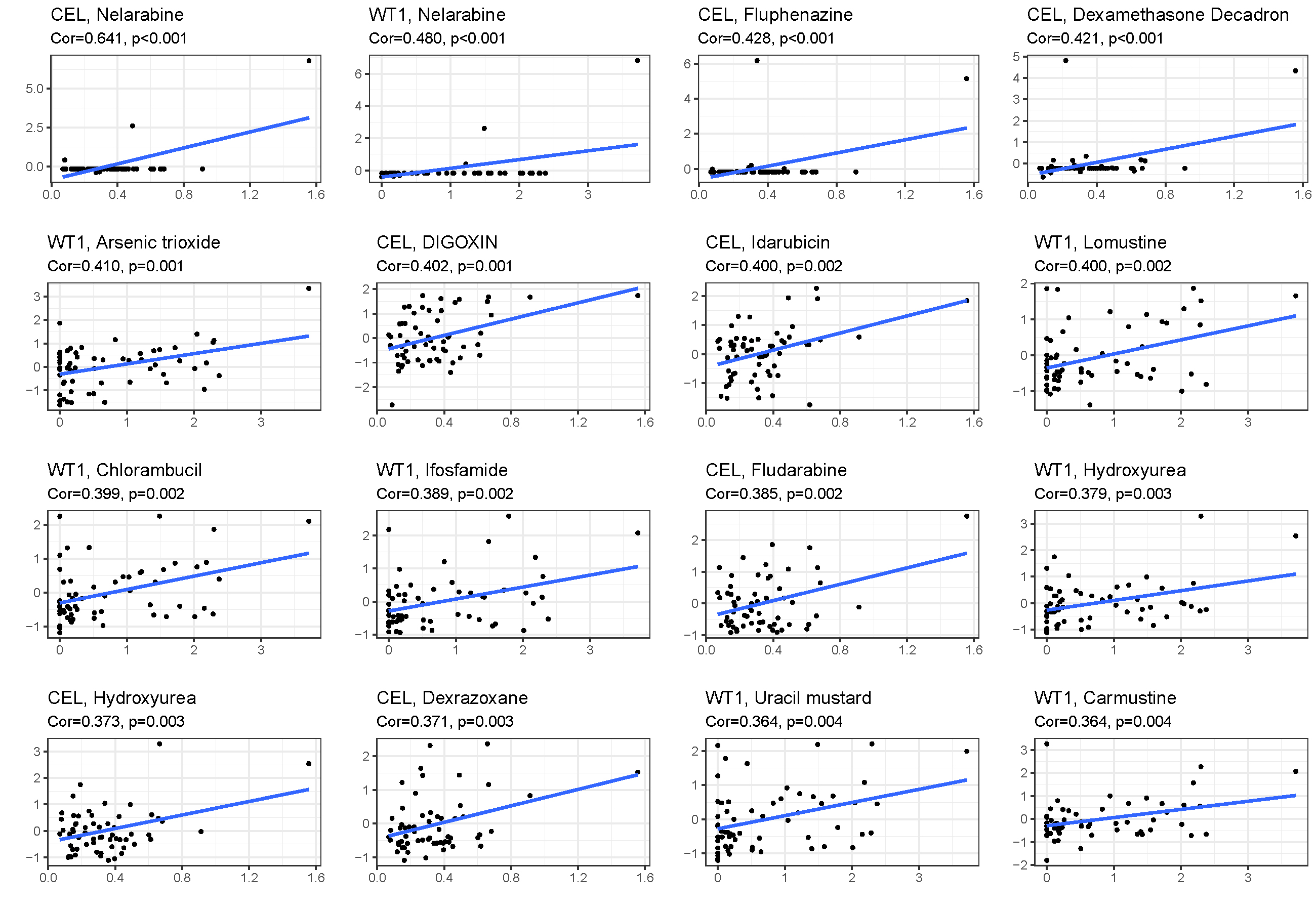


Figure S2. Prognostic signatures and drug sensitivity.

# Appendix 6

## **hub genes analysis**

**Table 4. Hub genes.**

| name | Betweenness | Closeness | Degree | Network |
| --- | --- | --- | --- | --- |
| ABCD1 | 18.68380558 | 0.385 | 4 | 2.666666667 |
| ACSBG1 | 260.1544786 | 0.463855422 | 11 | 4.516666667 |
| ABCD2 | 18.68380558 | 0.385 | 4 | 2.666666667 |
| ALDH3A2 | 687.3079393 | 0.48125 | 20 | 12.63235361 |
| PEX13 | 195.6808525 | 0.425414365 | 8 | 3.619047619 |
| ACAA2 | 226.8482457 | 0.52027027 | 28 | 22.35397612 |
| HSD17B10 | 1.448516218 | 0.401041667 | 8 | 7.142857143 |
| EHHADH | 653.2468569 | 0.55 | 30 | 21.09018398 |
| ACADL | 182.5450937 | 0.5 | 23 | 17.95765762 |
| ACADS | 1174.596122 | 0.57037037 | 32 | 19.16379333 |
| ACSS2 | 124.2263668 | 0.487341772 | 16 | 10.77517483 |
| ACLY | 151.1503064 | 0.43258427 | 8 | 5.657142857 |
| MECR | 408.8941982 | 0.452941176 | 12 | 8.181818182 |
| ACAT1 | 63.32067861 | 0.458333333 | 16 | 11.92857143 |
| ECI2 | 48.7081854 | 0.447674419 | 10 | 5.682539683 |
| ETFA | 7.32157706 | 0.413978495 | 13 | 9.810606061 |
| ETFB | 10.49122617 | 0.416216216 | 14 | 10.86192974 |
| CPT2 | 52.15078199 | 0.458333333 | 15 | 10.69338994 |
| ETFDH | 5.421699134 | 0.413978495 | 12 | 9.409090909 |
| ACSL1 | 181.780417 | 0.463855422 | 13 | 7.455555556 |
| ECI1 | 40.62193978 | 0.4375 | 14 | 9.918969919 |
| ACSBG2 | 350.5595994 | 0.48427673 | 13 | 7.733333333 |
| ACSS3 | 15.52921482 | 0.455621302 | 12 | 8.545454545 |
| ACSS1 | 124.2263668 | 0.487341772 | 16 | 10.77517483 |
| ACAT2 | 53.02257072 | 0.455621302 | 15 | 11.51428571 |
| HADHB | 99.0107872 | 0.487341772 | 24 | 18.74295873 |
| ACOX1 | 1060.4551 | 0.58778626 | 32 | 21.9791857 |
| ECHS1 | 33.33542801 | 0.455621302 | 19 | 15.62023671 |
| ACADM | 47.35612079 | 0.452941176 | 16 | 10.39284049 |
| ACADSB | 26.17657379 | 0.472392638 | 15 | 11.31493506 |
| ACADVL | 81.6378992 | 0.466666667 | 17 | 10.85912837 |
| HADH | 43.34917814 | 0.44 | 16 | 11.36391941 |
| HADHA | 35.89498846 | 0.455621302 | 19 | 15.45176351 |
| ACACB | 90.72797227 | 0.368421053 | 6 | 4 |
| MCAT | 3.351111111 | 0.30078125 | 2 | 0 |
| ACSL4 | 52.77889784 | 0.455621302 | 9 | 7.125 |
| ACSL5 | 50.77637259 | 0.447674419 | 8 | 6.571428571 |
| ACSL3 | 52.77889784 | 0.455621302 | 9 | 7.125 |
| GCDH | 4.760950764 | 0.413978495 | 9 | 6.75 |
| ALDH7A1 | 119.7435017 | 0.43258427 | 12 | 2.818181818 |
| ALDH1B1 | 119.7435017 | 0.447674419 | 14 | 8.391608392 |
| ALDH9A1 | 206.7696221 | 0.447674419 | 13 | 3.537878788 |
| ALDH2 | 207.8717107 | 0.463855422 | 15 | 9.064935065 |
| ACSM1 | 0 | 0.36492891 | 1 | 0 |
| ACSM4 | 0 | 0.36492891 | 1 | 0 |
| ACSM2A | 0 | 0.36492891 | 1 | 0 |
| ACSM2B | 0 | 0.36492891 | 1 | 0 |
| ACSM3 | 0 | 0.36492891 | 1 | 0 |
| SLC25A20 | 4.260253635 | 0.375609756 | 5 | 2.5 |
| ELOVL6 | 633.2871237 | 0.416216216 | 14 | 7.031529582 |
| ACOT1 | 0 | 0.327659574 | 4 | 4 |
| TECR | 229.8990307 | 0.361502347 | 12 | 9.289466089 |
| ACOT2 | 214.2435773 | 0.44 | 8 | 5.80952381 |
| ACOT4 | 214.2435773 | 0.44 | 8 | 5.80952381 |
| ACOT7 | 0 | 0.327659574 | 4 | 4 |
| CRAT | 22.05870339 | 0.416216216 | 7 | 4.416666667 |
| ELOVL1 | 398.1734033 | 0.381188119 | 11 | 5.799206349 |
| ADH1A | 2.33974359 | 0.337719298 | 7 | 4.666666667 |
| ADH1C | 2.33974359 | 0.337719298 | 7 | 4.666666667 |
| ADH1B | 2.33974359 | 0.337719298 | 7 | 4.666666667 |
| ADH4 | 2.33974359 | 0.334782609 | 5 | 1.5 |
| ADH5 | 2.33974359 | 0.334782609 | 5 | 1.5 |
| ADH6 | 2.33974359 | 0.334782609 | 5 | 1.5 |
| ADH7 | 2.33974359 | 0.334782609 | 5 | 1.5 |
| CYP4A11 | 0 | 0.327659574 | 2 | 2 |
| CYP4A22 | 0 | 0.327659574 | 2 | 2 |
| HSD17B12 | 57.02617963 | 0.318181818 | 10 | 8.777777778 |
| ELOVL4 | 2.666666667 | 0.299610895 | 7 | 5.666666667 |
| ELOVL3 | 0 | 0.288389513 | 3 | 3 |
| ELOVL7 | 0 | 0.288389513 | 3 | 3 |
| PTPLAD1 | 2.334920635 | 0.314285714 | 7 | 6.666666667 |
| PTPLA | 2.334920635 | 0.314285714 | 7 | 6.666666667 |
| PTPLAD2 | 2.334920635 | 0.314285714 | 7 | 6.666666667 |
| PTPLB | 2.334920635 | 0.314285714 | 7 | 6.666666667 |
| OXSM | 101.2841388 | 0.366666667 | 3 | 0 |
| PPT2 | 0 | 0.314285714 | 2 | 2 |
| PPT1 | 0 | 0.314285714 | 2 | 2 |
| PEX11G | 0 | 0.299610895 | 1 | 0 |

# Appendix 7

# **The gene expression profile and clinical characteristics**

**Table 5. The gene expression profile and clinical characteristics.**

| gene | Mean1 | Mean2 | logFC | pValue | fdr |
| --- | --- | --- | --- | --- | --- |
| PTGS2 | 0.669645864661654 | 2.47992010676157 | 1.88882339552422 | 0.000804697926357232 | 0.00128964101671845 |
| S100A8 | 21.0805069548872 | 164.018510676157 | 2.95987717543156 | 5.71935466436661e-26 | 1.14818314472344e-24 |
| ACY3 | 0.533893796992481 | 2.9561603202847 | 2.46909982035919 | 3.42287656611988e-17 | 2.4832216847037e-16 |
| CASP14 | 3.48419887218045 | 21.5267225978648 | 2.627229810684 | 1.34450540572927e-09 | 4.22300466530487e-09 |
| TBX10 | 0.293856766917293 | 1.5067128113879 | 2.35821943058016 | 6.12527860819331e-08 | 1.59511587914464e-07 |
| CAPN6 | 1.37924567669173 | 8.36308914590747 | 2.60015648309733 | 0.0211244334335911 | 0.0275270581759889 |
| TGM5 | 0.266568045112782 | 0.818520996441281 | 1.61851557723807 | 4.01706606448576e-11 | 1.49722523724706e-10 |
| SLC15A1 | 0.195021428571429 | 1.33852419928826 | 2.778938663522 | 1.3197684681216e-18 | 1.12718773442215e-17 |
| SPIB | 0.41261992481203 | 2.38994804270463 | 2.53409386081213 | 4.88168476742559e-10 | 1.61239020054882e-09 |
| SPRR1A | 1.03215751879699 | 3.43335498220641 | 1.73395586947143 | 4.63813886402771e-09 | 1.3759388885721e-08 |
| PICSAR | 0.285057142857143 | 1.85698007117438 | 2.7036352750155 | 4.86700136827022e-12 | 2.00071722010393e-11 |
| DNER | 0.385135714285714 | 1.19906014234875 | 1.63846520513226 | 4.97443593066637e-10 | 1.6408827237497e-09 |
| EEF1DP5 | 0.196848120300752 | 0.789283629893238 | 2.00346089108117 | 1.3799451986293e-25 | 2.65442690489187e-24 |
| MARCO | 1.50372462406015 | 5.34431725978648 | 1.82946526286652 | 2.70919152868612e-23 | 3.89738387022457e-22 |
| CCL20 | 0.449540977443609 | 1.53765800711744 | 1.77421013273092 | 7.17934956701048e-19 | 6.33494118479756e-18 |
| LIPG | 0.242452631578947 | 0.74474128113879 | 1.61903641578396 | 9.43715517699961e-13 | 4.20762938076742e-12 |
| MGAM2 | 0.150892669172932 | 0.866776868327402 | 2.52213793571307 | 0.0227705099723102 | 0.0295021367466068 |
| PPP1R1B | 13.2365340225564 | 62.0129135231317 | 2.22804326965053 | 1.90702639186333e-06 | 4.19888888948546e-06 |
| SYNPO2L | 2.51733270676692 | 0.702069928825623 | -1.84220926419754 | 5.44277418885098e-18 | 4.35680294642359e-17 |
| CRYAB | 9.16978834586466 | 28.881231316726 | 1.65517191162279 | 8.11135706228769e-10 | 2.60211511592349e-09 |
| KRT6C | 0.106725 | 1.9393024911032 | 4.18356778390426 | 2.22303254614396e-11 | 8.50490671863546e-11 |
| SNORA63C | 1.59697030075188 | 0.275071352313167 | -2.53745968172753 | 0.00497631925592704 | 0.00710494801008511 |
| IGDCC3 | 1.16939830827068 | 0.295408362989324 | -1.98498383494715 | 5.90744676361697e-49 | 1.63013308238936e-46 |
| RBM24 | 6.32243270676692 | 1.81410800711744 | -1.80121942393894 | 1.91943568482921e-49 | 5.49646705446281e-47 |
| GSDMC | 0.523648684210526 | 2.76413434163701 | 2.40015659724522 | 3.31420890187762e-33 | 1.5572677555355e-31 |
| RNU4-2 | 183.880333458647 | 24.682584519573 | -2.89720181506514 | 0.00320283063075434 | 0.0046974642909701 |
| FGG | 0.586929323308271 | 5.97199982206406 | 3.34695542962982 | 0.0132938804399768 | 0.0178234296322905 |
| C6orf223 | 0.171556203007519 | 1.02637330960854 | 2.58080236347565 | 3.51693240370035e-19 | 3.20772133960097e-18 |
| COL22A1 | 0.626198872180451 | 1.89723362989324 | 1.59920453139528 | 4.48166598605736e-08 | 1.18705488080964e-07 |
| FOXCUT | 0.493799812030075 | 2.74749537366548 | 2.4761188594963 | 4.7391782858469e-13 | 2.17893089501055e-12 |
| LORICRIN | 0.0283437969924812 | 2.32013736654804 | 6.3550333766133 | 0.00836265809632467 | 0.0115563166768025 |
| IGHV3-73 | 9.02458477443609 | 64.5502081850534 | 2.83848928893481 | 0.0219791088070239 | 0.028540121009942 |
| CEL | 1.02050620300752 | 3.69327615658363 | 1.85561618929639 | 0.00474550656686797 | 0.00680036398192833 |
| SNORC | 0.850891541353383 | 2.76532918149466 | 1.70040407135052 | 0.0371992214619474 | 0.0465895844304321 |
| CST9 | 17.4019855263158 | 3.29046370106762 | -2.40288911173687 | 1.3620966996885e-54 | 9.39660982326016e-52 |
| PLPPR3 | 5.24831015037594 | 1.74824964412811 | -1.58594176743313 | 3.88373166776334e-48 | 9.99040602061766e-46 |
| AC008663.1 | 14.5018537593985 | 4.67748790035587 | -1.6324315038399 | 9.89667321678169e-54 | 5.77699266965753e-51 |
| KIRREL3-AS1 | 0.234789473684211 | 1.05454590747331 | 2.16718226657319 | 2.34662023626831e-18 | 1.95900194311574e-17 |
| AZU1 | 1.18401071428571 | 0.364267259786477 | -1.70061289908775 | 4.26085540038425e-42 | 5.17336019293054e-40 |
| FDCSP | 84.5079327067669 | 449.951957295374 | 2.41261229109341 | 5.78804146462849e-06 | 1.20335760696804e-05 |
| SNORA11 | 15.4860287593985 | 2.20770925266904 | -2.8103451345096 | 6.57359950124653e-21 | 7.30897579710026e-20 |
| IDO1 | 2.32309304511278 | 8.34322758007117 | 1.84455866016325 | 3.54284919403676e-18 | 2.88929727124642e-17 |
| RPL6P4 | 2.03479304511278 | 0.617811209964413 | -1.71964411471159 | 2.0974220448979e-17 | 1.55889198704287e-16 |
| AP000851.2 | 0.57724530075188 | 2.2316334519573 | 1.95084365406777 | 6.33329994851245e-06 | 1.3088302467126e-05 |
| PGR | 16.8069233082707 | 5.34768042704626 | -1.65207048872796 | 3.03782950855732e-45 | 5.42413393545582e-43 |
| CPA4 | 0.602323120300752 | 2.95946209964413 | 2.29672543777308 | 5.3664644080862e-18 | 4.30252669421681e-17 |
| ADCY1 | 7.24400695488722 | 2.11319128113879 | -1.77736456883476 | 3.3962317926186e-51 | 1.22725261706125e-48 |
| IGLV4-3 | 0.456177443609023 | 1.64598042704626 | 1.85128016298369 | 0.0271055687820591 | 0.0346864432888121 |
| CA9 | 1.25020432330827 | 6.82721352313167 | 2.44913297670501 | 5.74234106038795e-20 | 5.74120621037602e-19 |
| RARRES1 | 8.72885263157895 | 53.9466386120996 | 2.62766913325697 | 4.89724235904028e-29 | 1.35383328384616e-27 |
| KLHDC7B | 2.65201447368421 | 8.70011476868327 | 1.71394578343248 | 1.83064220240569e-09 | 5.66435406848342e-09 |
| MSLN | 2.39064210526316 | 20.5855991103203 | 3.10616536610131 | 4.09705029305177e-15 | 2.35980767733005e-14 |
| SLC25A24P2 | 2.43908872180451 | 0.81142615658363 | -1.5878105229465 | 2.02196110403722e-15 | 1.19638610822506e-14 |
| B4GALNT2 | 0.231642293233083 | 1.05690800711744 | 2.18987922089667 | 0.00135855765050205 | 0.00210353289753822 |
| ROPN1 | 0.221110526315789 | 1.45580711743772 | 2.7189796092015 | 4.0504335456615e-08 | 1.07885977399973e-07 |
| IGSF1 | 4.02641015037594 | 1.33391832740214 | -1.59382380341988 | 3.58848731913597e-11 | 1.34574924740614e-10 |
| IBSP | 1.86683082706767 | 5.995271886121 | 1.68323398556904 | 0.0149080010028962 | 0.0198402956174111 |
| CLSTN2 | 24.4843182330827 | 7.75226192170819 | -1.65916880441066 | 7.40814555811737e-58 | 8.03095893825338e-55 |
| PRSS33 | 0.125522180451128 | 1.71708914590747 | 3.77395071764086 | 5.50919111615567e-17 | 3.90350110036856e-16 |
| CRABP1 | 7.01617293233083 | 33.2381583629893 | 2.24408423606041 | 7.09331906634409e-20 | 7.0179467711802e-19 |
| S100A7 | 42.648945112782 | 234.118049644128 | 2.45665420204059 | 7.94295427721976e-11 | 2.86614876522502e-10 |
| S100B | 2.67748270676692 | 9.50660355871886 | 1.82805274192105 | 2.21459078086551e-06 | 4.83470142134578e-06 |
| PSAT1 | 3.51731184210526 | 13.1388215302491 | 1.90129072682572 | 4.82584098925919e-31 | 1.72740067674497e-29 |
| IGLV8-61 | 17.247412406015 | 59.4318806049822 | 1.78485709937702 | 0.00480534123200882 | 0.00687895339352932 |
| HRCT1 | 2.03996917293233 | 7.76950516014235 | 1.92927536527466 | 6.05957941460775e-10 | 1.97310098209616e-09 |
| GSTA1 | 1.31071109022556 | 5.28477864768683 | 2.01149332712703 | 1.51627491656048e-08 | 4.24037302536178e-08 |
| TAFA3 | 0.471990601503759 | 1.62707580071174 | 1.78545142615492 | 2.69517984560317e-08 | 7.33017513539483e-08 |
| FUT3 | 0.714663909774436 | 3.14618896797153 | 2.13826848549 | 3.28018711768032e-32 | 1.34914362832071e-30 |
| SCARNA5 | 74.7572140977444 | 3.43143932384342 | -4.44532705693525 | 0.000413257964062285 | 0.000686066081882881 |
| GLYATL2 | 5.17698627819549 | 31.1202386120996 | 2.58766872272579 | 9.92229192491541e-18 | 7.65585279839559e-17 |
| ELOVL2 | 24.574092481203 | 1.03093879003559 | -4.57510755578601 | 2.59959033816307e-178 | 3.94539825623009e-174 |
| LINC01436 | 1.15592236842105 | 5.40399893238434 | 2.22498287974243 | 1.06927432199294e-09 | 3.39742155721997e-09 |
| LINC01819 | 0.480999248120301 | 2.14831423487544 | 2.15909848845745 | 2.54414678884926e-06 | 5.51292344579743e-06 |
| CEROX1 | 0.457034210526316 | 1.44557971530249 | 1.66127410229196 | 6.11489372906208e-05 | 0.000112301236841693 |
| ECEL1 | 0.389513157894737 | 6.25915604982206 | 4.00622417622548 | 1.1346210906767e-16 | 7.74983991593172e-16 |
| LY6D | 0.675516353383459 | 7.75907615658363 | 3.52182228559624 | 2.90079284296161e-13 | 1.36597371944239e-12 |
| CALML5 | 37.9344610902256 | 161.331030604982 | 2.08844300732009 | 1.41089700558935e-15 | 8.4972951800911e-15 |
| NPY5R | 1.6300765037594 | 0.528023665480427 | -1.62626517924088 | 1.42259401405852e-14 | 7.69173827978844e-14 |
| NPY1R | 121.846903007519 | 25.4276304270463 | -2.26060065219385 | 7.80363181063087e-38 | 5.92178599949724e-36 |
| ACAN | 0.462941353383459 | 2.85389911032028 | 2.62403298844047 | 0.000226504392362794 | 0.000389712862814888 |
| SOX11 | 0.640053195488722 | 3.38267170818505 | 2.40189944977041 | 1.10783880224166e-28 | 2.96537380980982e-27 |
| RN7SL674P | 2.39604135338346 | 0.670803558718861 | -1.83669056011862 | 9.69309392459842e-27 | 2.11977069875548e-25 |
| SNORA38B | 1.3804772556391 | 0.418549288256228 | -1.72169768938977 | 0.00395714430297885 | 0.00573013825840187 |
| DSCAM-AS1 | 16.2712902255639 | 4.42844982206406 | -1.87745497631798 | 7.72372156556365e-24 | 1.19615226735265e-22 |
| KRT16 | 8.25086259398496 | 45.1285348754448 | 2.45142308113363 | 1.27951345727746e-14 | 6.95537011165467e-14 |
| AKR1B15 | 0.428253947368421 | 5.53037028469751 | 3.69083762970086 | 2.1173427286673e-11 | 8.12513542174048e-11 |
| NKX2-5 | 0.266745864661654 | 0.809756761565836 | 1.60202270450182 | 2.17517205326077e-07 | 5.34184243565351e-07 |
| DCD | 15.663172556391 | 173.873382562278 | 3.47258873086973 | 7.49462398739335e-08 | 1.9351124235568e-07 |
| HORMAD1 | 0.439882518796992 | 2.87518879003559 | 2.70846651519472 | 2.09016018369716e-16 | 1.38525594357955e-15 |
| ACTL8 | 0.748397180451128 | 5.77527241992883 | 2.94801297172066 | 1.05984367038395e-15 | 6.46773115617901e-15 |
| AL031429.2 | 1.66466541353383 | 0.461516725978648 | -1.85077739539411 | 2.60736429633534e-53 | 1.41328456876719e-50 |
| DUSP9 | 0.345183646616541 | 1.07881850533808 | 1.64401615165074 | 7.33871712933141e-20 | 7.25600715777608e-19 |
| FABP7 | 3.63178515037594 | 33.9334135231317 | 3.22395580176816 | 7.49505048570802e-10 | 2.41307554564257e-09 |
| SLURP1 | 0.447081578947368 | 2.14115587188612 | 2.25977981585986 | 1.54055989513412e-15 | 9.24153261994092e-15 |
| FAM3D | 0.260137406015038 | 1.89042526690391 | 2.86136504803313 | 1.32823352637623e-07 | 3.34638118024768e-07 |
| UPK2 | 0.301920864661654 | 0.985468149466192 | 1.70663878407524 | 1.13676855668928e-12 | 5.01970799676264e-12 |
| CHI3L1 | 14.3780092105263 | 56.8744866548043 | 1.98391768557704 | 8.46432527377776e-16 | 5.22845196093305e-15 |
| AC025423.3 | 2.27147368421053 | 0.36600409252669 | -2.63369690597094 | 4.00466215885665e-20 | 4.07911124731325e-19 |
| ORM1 | 2.41724943609023 | 12.3066309608541 | 2.34799760883786 | 1.14747002534799e-05 | 2.30206907795194e-05 |
| ROPN1B | 0.428863157894737 | 2.30880782918149 | 2.42855880751713 | 1.30499932587152e-09 | 4.10486523704706e-09 |
| FMO3 | 0.817464285714286 | 3.73115355871886 | 2.19039412964706 | 0.0227214605448159 | 0.0294461281435122 |
| AC022509.2 | 0.443621616541353 | 1.43677615658363 | 1.69543374260266 | 0.000360427866874911 | 0.000602645558616341 |
| LINC01956 | 0.640091165413534 | 3.62391957295374 | 2.50120163578333 | 3.93841460072609e-18 | 3.19985644514025e-17 |
| KCNG1 | 0.379068796992481 | 1.52529270462633 | 2.00855451210558 | 1.14287047775884e-23 | 1.7190629574773e-22 |
| LINC02613 | 0.304467481203008 | 0.938716903914591 | 1.62440199020246 | 0.000734287313258277 | 0.00118354700014028 |
| S100A2 | 6.27557105263158 | 28.2802318505338 | 2.17197529807483 | 3.30425207528467e-06 | 7.06517877287075e-06 |
| HNRNPA1P57 | 5.55198984962406 | 1.30221903914591 | -2.09203279356325 | 1.58220066056744e-26 | 3.35846984971077e-25 |
| AC103563.3 | 0.299257518796992 | 1.03444839857651 | 1.78940228077116 | 5.46693334675644e-05 | 0.000101147930517765 |
| AC114501.1 | 1.3177507518797 | 0.334050889679715 | -1.97993770893498 | 8.97564037613841e-28 | 2.19361181946301e-26 |
| SCARNA6 | 18.0954894736842 | 1.47983096085409 | -3.61212583917731 | 2.93036571686654e-13 | 1.3786162580559e-12 |
| EN1 | 1.84004172932331 | 9.81376921708185 | 2.41506886028389 | 9.64591794252844e-24 | 1.46396096613754e-22 |
| GLRA3 | 2.28474398496241 | 0.40274537366548 | -2.50409259247088 | 1.31509010103341e-19 | 1.26004561006213e-18 |
| KRT20 | 0.0282304511278195 | 2.64845676156584 | 6.55175596180578 | 0.011013001946966 | 0.0149396076643817 |
| LINC01488 | 1.40979718045113 | 0.415570106761566 | -1.76232383958741 | 1.48095942091828e-59 | 2.49739123680853e-56 |
| VGLL1 | 1.2961727443609 | 14.7912795373665 | 3.51241695197675 | 1.96900459709295e-15 | 1.16732745195624e-14 |
| KRT75 | 0.299587406015038 | 1.28062348754448 | 2.09579749286159 | 3.95216326451027e-06 | 8.36218902348702e-06 |
| DHRS2 | 37.0099902255639 | 12.1884991103203 | -1.60239427094198 | 6.38536489659363e-33 | 2.90153562282043e-31 |
| TMPRSS11E | 0.503217669172932 | 1.66721868327402 | 1.72818886534094 | 0.00221340886222044 | 0.00333163803450557 |
| CCL18 | 2.76528308270677 | 10.3131176156584 | 1.89898143760442 | 2.21419707831192e-18 | 1.85559740792601e-17 |
| UGT8 | 0.825933646616541 | 2.89330729537367 | 1.80862176964593 | 1.48347772023258e-09 | 4.63552426600162e-09 |
| LINC02487 | 0.118567857142857 | 1.55794822064057 | 3.71586242115643 | 1.51598255943287e-08 | 4.24033676824782e-08 |
| HUS1B | 0.956765789473684 | 0.286747330960854 | -1.73838574711466 | 0.000198379451326766 | 0.000343755953887124 |
| NPTX1 | 1.54910469924812 | 0.251131672597865 | -2.62491875761139 | 0.000110986286615698 | 0.000198215918094428 |
| RDH10 | 5.57624736842105 | 17.8336791814947 | 1.67723790195257 | 2.3680550405053e-14 | 1.24965129866999e-13 |
| SMOC1 | 0.911485714285714 | 4.36102046263345 | 2.25837380960358 | 2.94551199308028e-23 | 4.21736184141315e-22 |
| RN7SL314P | 9.98993458646617 | 2.96429857651246 | -1.75278446213956 | 5.39069748017986e-27 | 1.20492806563608e-25 |
| GJB3 | 0.780955827067669 | 2.87965409252669 | 1.88258267070084 | 4.58094305608588e-07 | 1.08102245027716e-06 |
| ATP13A5 | 0.334610338345865 | 1.2737615658363 | 1.92854132056063 | 3.20545500863352e-13 | 1.50198180506425e-12 |
| FGFBP1 | 0.539699060150376 | 3.23490142348754 | 2.58349466985747 | 2.06770924924646e-09 | 6.36673225315754e-09 |
| DKK1 | 1.23636090225564 | 4.55261298932384 | 1.88059488593722 | 9.32335273731666e-15 | 5.14765115851185e-14 |
| SCARNA21 | 15.914244924812 | 3.60490106761566 | -2.14228713576282 | 0.00300208224722731 | 0.00442772133607133 |
| KRT81 | 12.356384962406 | 82.4103097864769 | 2.7375681104902 | 8.06745327560069e-10 | 2.58967297723756e-09 |
| CLCA2 | 3.7111295112782 | 12.3239928825623 | 1.73153949998772 | 0.00892125224688255 | 0.0122743038120693 |
| SIX3 | 0.594490037593985 | 1.83809466192171 | 1.62848652862792 | 9.78263475171059e-09 | 2.80081206615189e-08 |
| TRPV6 | 0.63036954887218 | 1.99572793594306 | 1.66264531161969 | 1.84820514896472e-27 | 4.36919151804323e-26 |
| AC025154.2 | 0.256754887218045 | 1.02377722419929 | 1.99543817341105 | 2.86798030005014e-22 | 3.70836922840425e-21 |
| SLPI | 41.0503930451128 | 249.572759430605 | 2.60399253379709 | 3.73487559765101e-20 | 3.81969049498311e-19 |
| GAL | 1.38487932330827 | 4.50607455516014 | 1.70211091678199 | 2.97206818546939e-31 | 1.09218108597746e-29 |
| AC027031.2 | 0.46993007518797 | 1.41453879003559 | 1.58981373216905 | 2.62082746984651e-20 | 2.73568692899593e-19 |
| CXCL5 | 0.107870864661654 | 1.30229501779359 | 3.59367915069047 | 5.22296654183696e-14 | 2.65202285732551e-13 |
| CWH43 | 0.260813909774436 | 0.784162099644128 | 1.58813110108093 | 4.11301153530675e-06 | 8.68556784073334e-06 |
| LINC01133 | 0.363797744360902 | 1.24789199288256 | 1.77828456890855 | 4.23379510362311e-25 | 7.61740460532839e-24 |
| A2ML1 | 0.676896992481203 | 3.62884181494662 | 2.4225009576193 | 1.52380062814875e-29 | 4.49062565697351e-28 |
| FGB | 0.810618045112782 | 6.15069412811388 | 2.92365503641093 | 0.0385368868988743 | 0.0481378051410877 |
| AC021134.1 | 3.02165789473684 | 0.544088790035587 | -2.47342632138609 | 3.54323909853138e-17 | 2.56808690536823e-16 |
| XDH | 0.917650187969925 | 4.76721298932384 | 2.3771298812516 | 7.46528500631952e-05 | 0.000135835787724387 |
| PAX7 | 0.242096428571429 | 0.804401423487545 | 1.73233383794722 | 0.0210529730703848 | 0.0274455396228509 |
| UGT2B4 | 6.13152218045113 | 1.53358113879004 | -1.99934077569158 | 2.36973142343305e-11 | 9.04107939000588e-11 |
| CXCL1 | 0.671468233082707 | 3.27269466192171 | 2.285087954121 | 9.94587451025429e-17 | 6.85210902765359e-16 |
| KRT6B | 7.87095206766917 | 41.593359430605 | 2.40174315461388 | 2.36333425713234e-06 | 5.14167488825939e-06 |
| AC020907.1 | 0.295842481203008 | 0.89525409252669 | 1.59746797898314 | 3.04679803169401e-19 | 2.79910736846368e-18 |
| C6orf15 | 0.269350563909774 | 7.56147206405694 | 4.81111013431992 | 0.000673819170386595 | 0.0010914144662708 |
| KRT83 | 0.208916729323308 | 1.00829946619217 | 2.27092425656463 | 1.08000819996049e-22 | 1.45247449142503e-21 |
| CA6 | 0.0719877819548872 | 1.28363309608541 | 4.15633701464466 | 5.43032090099882e-09 | 1.59720892082285e-08 |
| LBP | 2.88686879699248 | 26.6470245551601 | 3.20639700231272 | 6.99422207268362e-06 | 1.43816973847879e-05 |
| AC073508.1 | 0.939064285714286 | 0.294425266903915 | -1.67332243862587 | 3.02553891667472e-20 | 3.13651667611832e-19 |
| LINC01016 | 1.42243533834586 | 0.253302491103203 | -2.48942990097801 | 3.13379073440899e-69 | 1.58538473253751e-65 |
| RPL39P40 | 0.306521240601504 | 1.27470747330961 | 2.05610725264693 | 3.56093866734234e-21 | 4.06654372868734e-20 |
| STAC | 0.385951691729323 | 1.27092526690391 | 1.71938701267759 | 6.69653509733671e-15 | 3.75169114700182e-14 |
| WNT6 | 0.410927255639098 | 1.93785800711744 | 2.23750793521251 | 6.36576619627646e-17 | 4.47905579790857e-16 |
| MMP1 | 6.86382838345865 | 24.0765514234875 | 1.81054337752231 | 6.93767668126201e-16 | 4.32237762690942e-15 |
| BTN1A1 | 0.0352990601503759 | 1.35133078291815 | 5.25860728325484 | 6.21279587217658e-08 | 1.61596577467051e-07 |
| CHI3L2 | 6.0831287593985 | 20.4055991103203 | 1.74607962222795 | 7.97176497900134e-13 | 3.58269105970694e-12 |
| CHODL | 0.272954135338346 | 1.3493165480427 | 2.30549838288582 | 2.06413196180581e-13 | 9.87932222779148e-13 |
| AC093838.1 | 4.40379078947368 | 1.45656423487544 | -1.59617660641431 | 3.88115007279349e-47 | 8.93667109341442e-45 |
| AP000851.1 | 0.560545864661654 | 2.75548220640569 | 2.29740048462417 | 0.00913781615444538 | 0.0125529177929053 |
| AC008663.3 | 4.90184323308271 | 1.4889896797153 | -1.71899059125671 | 1.78437199929259e-46 | 3.76130747684217e-44 |
| AQP5 | 2.62214981203008 | 17.0231480427046 | 2.69867583628361 | 1.51357555192174e-18 | 1.28620023244772e-17 |
| NXNL2 | 2.86316015037594 | 0.835466548042705 | -1.77695440023693 | 1.1793055718846e-67 | 4.47458016612314e-64 |
| BBOX1 | 1.28260789473684 | 4.42224128113879 | 1.78569754951074 | 7.26787208408464e-10 | 2.34441008756966e-09 |
| MPZ | 0.853140413533835 | 3.80512704626335 | 2.15708951231459 | 1.91064212514625e-06 | 4.20623956097253e-06 |
| PI3 | 1.75179172932331 | 39.0916332740214 | 4.47995669452588 | 1.72121346577275e-14 | 9.21441155909454e-14 |
| IGLV1-36 | 4.46641823308271 | 19.1513003558719 | 2.10025209734434 | 0.0064361465438899 | 0.00903119416573752 |
| S100A7A | 0.631432706766917 | 4.12945765124555 | 2.70925141949008 | 3.14429901207242e-11 | 1.18473252498071e-10 |
| GTSF1 | 0.268167105263158 | 0.903667615658363 | 1.75265994259037 | 3.43005631403883e-08 | 9.21053869040469e-08 |
| FCRLB | 5.17545620300752 | 1.6750024911032 | -1.62752279685173 | 3.00768024854782e-07 | 7.25024827385805e-07 |
| SPANXB1 | 3.73722894736842 | 0.836848754448399 | -2.15893013780906 | 4.43713825359043e-05 | 8.3015837370244e-05 |
| SERPINB7 | 0.29707462406015 | 1.62730818505338 | 2.45359021789961 | 1.04028892951564e-06 | 2.35437892682059e-06 |
| PSORS1C2 | 0.149223496240601 | 1.69486512455516 | 3.50562384928924 | 3.60320572261065e-13 | 1.67850992179441e-12 |
| PDZK1 | 14.7124133458647 | 4.30395782918149 | -1.77329807067305 | 8.91620671486554e-46 | 1.7199742020509e-43 |
| GABRP | 9.94133890977444 | 60.1638523131673 | 2.59738487261554 | 6.60598259793508e-05 | 0.000120823087357027 |
| TMEM26 | 4.07348759398496 | 1.21130409252669 | -1.74970342169438 | 1.10023078871334e-51 | 4.51302775143307e-49 |
| IGHM | 142.636242481203 | 456.355245017794 | 1.67781670962319 | 9.35023732755755e-05 | 0.00016833754676197 |
| CHRNA9 | 4.79981672932331 | 0.684215836298932 | -2.81045591963696 | 3.57201470414747e-08 | 9.57141016328498e-08 |
| KCNF1 | 6.82272462406015 | 2.0637706405694 | -1.72506534386137 | 4.15504737483711e-19 | 3.7581140648333e-18 |
| AL109615.3 | 0.741900187969925 | 2.98889768683274 | 2.01031650177869 | 3.52480723752358e-05 | 6.67531812377032e-05 |
| SNORD94 | 9.94382556390977 | 2.28605088967972 | -2.12094346895642 | 0.000254879259422977 | 0.000435227556285162 |
| SPRR1B | 0.451517857142857 | 5.72305053380783 | 3.66392939446176 | 4.49933479245789e-07 | 1.0628234108192e-06 |
| RLN2 | 4.37428439849624 | 1.10435088967972 | -1.98584838367272 | 3.0702423115026e-37 | 2.18765575406925e-35 |
| AC016735.1 | 0.288421804511278 | 1.22652597864769 | 2.0883256508001 | 6.49957468318395e-07 | 1.50486720010195e-06 |
| IGHV6-1 | 10.106870112782 | 58.4520051601423 | 2.53191622258137 | 0.00294138006198363 | 0.0043438090104822 |
| CBLN2 | 5.23512612781955 | 1.73809733096085 | -1.59071542258462 | 5.22075583666072e-09 | 1.53795441251941e-08 |
| NCCRP1 | 3.22482819548872 | 14.4186137010676 | 2.16063825388168 | 8.84291615043765e-16 | 5.44900277771791e-15 |
| RNVU1-15 | 1.49986597744361 | 0.490326690391459 | -1.61301839212684 | 4.37475187823281e-05 | 8.19092144780895e-05 |
| IGHV1-69-2 | 7.51388703007519 | 47.1939941281139 | 2.65097194439413 | 0.0086512408580652 | 0.0119233456686211 |
| RAET1L | 0.171030827067669 | 0.876569039145907 | 2.35761133917735 | 1.63434173492692e-26 | 3.46314795775023e-25 |
| ROCR | 0.299154323308271 | 1.21293149466192 | 2.01953625282455 | 0.0088355984341325 | 0.0121641761098357 |
| PCP4L1 | 0.849221240601504 | 2.79869733096085 | 1.72054311178142 | 1.84263829694815e-10 | 6.36162907934078e-10 |
| IGF2BP2 | 0.710348120300752 | 2.32959466192171 | 1.71348083024861 | 8.09513916111658e-13 | 3.63275952242065e-12 |
| CACNG6 | 1.63830601503759 | 0.521899466192171 | -1.65036102804964 | 0.00780287158024018 | 0.0108318102966528 |
| IL20RB | 0.304921804511278 | 1.13812117437722 | 1.90014294365165 | 7.72775774676926e-17 | 5.3948564545868e-16 |
| ULBP3 | 0.409357142857143 | 1.34277953736655 | 1.71379048287249 | 1.19605429476018e-32 | 5.24639191664025e-31 |
| LCN2 | 5.97722593984962 | 28.8300944839858 | 2.27002758199027 | 6.53307056957825e-11 | 2.38184236858712e-10 |
| LINC02188 | 0.344072744360902 | 2.21274234875445 | 2.685049954841 | 1.04565961875443e-07 | 2.65561847955756e-07 |
| WNK4 | 8.69086917293233 | 2.66781850533808 | -1.70383994632604 | 1.29427656251165e-52 | 6.77352944456528e-50 |
| KLK8 | 0.274556015037594 | 0.959382028469751 | 1.80500489611857 | 0.0042233828155149 | 0.00609241336289988 |
| MSMB | 46.678245112782 | 10.3900870106762 | -2.16754258738153 | 1.16276271388168e-08 | 3.30101939928587e-08 |
| ORM2 | 1.34074097744361 | 8.95333238434164 | 2.73939420128093 | 4.4318295720781e-16 | 2.82257144000962e-15 |
| VMO1 | 2.07561616541353 | 8.38738985765124 | 2.0146822390131 | 7.62936938871802e-08 | 1.96756056435979e-07 |
| VN1R53P | 5.97431165413534 | 1.87304359430605 | -1.6733880207098 | 8.88213079923299e-30 | 2.69608198279918e-28 |
| PROM1 | 4.12685281954887 | 16.014096797153 | 1.95622853973622 | 2.04082353206515e-15 | 1.20660610619995e-14 |
| SOX8 | 0.522116165413534 | 2.71850035587189 | 2.380368284741 | 1.68372191751696e-05 | 3.31094163541784e-05 |
| MCCD1 | 1.56370187969925 | 0.451553914590747 | -1.79199533068942 | 6.64489473164429e-42 | 7.87887244860667e-40 |
| IGLV2-11 | 36.090112406015 | 113.319322775801 | 1.65071834319051 | 0.00406563501276006 | 0.00587882456065734 |
| STC2 | 150.129371992481 | 47.7039338078292 | -1.65402611482628 | 1.60763099272948e-51 | 6.42079357280403e-49 |
| IVL | 0.146404887218045 | 1.14340427046263 | 2.96529996477268 | 7.81282993411037e-14 | 3.8907210300253e-13 |
| PTGER3 | 6.70518327067669 | 2.1981346975089 | -1.60899697079983 | 6.27215527740812e-52 | 2.64423612903397e-49 |
| PPP1R14C | 1.21658383458647 | 8.21840658362989 | 2.75602296616667 | 5.17061922402847e-20 | 5.20387851214059e-19 |
| SCRG1 | 0.203768045112782 | 1.26142491103203 | 2.63005459837425 | 0.000580648011748558 | 0.000947478214633681 |
| CALB2 | 3.95965770676692 | 14.302459252669 | 1.85281560736937 | 2.2932072300686e-05 | 4.44211948063193e-05 |
| TRH | 27.1943441729323 | 7.39707366548043 | -1.87828008536651 | 5.70348387186378e-34 | 2.93429744824666e-32 |
| KLK5 | 5.8621712406015 | 25.3285208185053 | 2.11125580985784 | 0.00191856974470887 | 0.00291268710767696 |
| COL11A2 | 0.555572368421053 | 2.43703024911032 | 2.13307740502 | 5.21159540289861e-08 | 1.36939722004488e-07 |
| ATP1A2 | 1.32060845864662 | 0.430049110320285 | -1.61862946593029 | 2.15176139224439e-24 | 3.58084239584354e-23 |
| MKX | 4.56553496240601 | 1.43389893238434 | -1.67084057657463 | 4.40244723011202e-08 | 1.1668940489864e-07 |
| SLC6A14 | 0.762918421052632 | 5.18154110320285 | 2.76378054778201 | 3.08379759316395e-09 | 9.31584316708783e-09 |
| LINC02747 | 8.46196409774436 | 2.64278185053381 | -1.67893522260366 | 3.05959042639139e-49 | 8.59914887061891e-47 |
| AC087491.1 | 0.42468007518797 | 1.46931387900356 | 1.79069429329595 | 0.000553246057825466 | 0.000904028361285217 |
| TTYH1 | 0.636667669172932 | 2.84394964412811 | 2.15928351113832 | 0.0020608305189404 | 0.00311464098645274 |
| ELF5 | 2.43253176691729 | 13.9276197508897 | 2.51741816978144 | 8.95325932585155e-17 | 6.19341917905419e-16 |
| GABRE | 0.487258458646617 | 1.72222366548043 | 1.82151338242795 | 3.76239074943432e-14 | 1.94422214518777e-13 |
| MMP12 | 2.04622406015038 | 6.63262953736655 | 1.69661681915865 | 8.8191323653579e-11 | 3.16051881721457e-10 |
| PADI2 | 8.10024078947368 | 26.6739944839858 | 1.71939718792721 | 1.07834728491534e-27 | 2.60606317566244e-26 |
| STAC2 | 6.66803327067669 | 23.3969555160142 | 1.81098760619951 | 3.56024792469983e-05 | 6.73659357004934e-05 |
| BARX1 | 0.446965977443609 | 1.79717775800712 | 2.0074961878456 | 3.60721049108073e-08 | 9.66060236864871e-08 |
| LCAL1 | 0.161774248120301 | 1.34268238434164 | 3.05306419404263 | 2.22500848798735e-22 | 2.89117755326918e-21 |
| EPHX3 | 0.416357706766917 | 1.41314252669039 | 1.76301154675058 | 7.05134747422104e-09 | 2.04937381494165e-08 |
| ELOVL2-AS1 | 1.23515958646617 | 0.0641138790035587 | -4.26791694734891 | 8.54234652861656e-167 | 6.48235966324068e-163 |
| KLK6 | 0.988752067669173 | 9.59199715302491 | 3.27815051889799 | 4.18104992637661e-07 | 9.90722790517062e-07 |
| AP001783.1 | 0.220723684210526 | 1.19168540925267 | 2.43269008389356 | 3.38699329876975e-17 | 2.45836428959486e-16 |
| LEMD1 | 0.26342969924812 | 2.05714982206406 | 2.96515695873651 | 4.15751835689307e-11 | 1.5472941663209e-10 |
| COL9A3 | 1.37800883458647 | 5.76536992882562 | 2.06482804246921 | 8.60393405714169e-11 | 3.090696028053e-10 |
| MAPK4 | 0.180375751879699 | 0.971500355871886 | 2.42920911581637 | 1.33023078919551e-10 | 4.67660706222383e-10 |
| ARHGAP36 | 5.44104605263158 | 1.1332859430605 | -2.26337212115453 | 2.00326084339906e-11 | 7.70488844913014e-11 |
| IL1R2 | 0.293401127819549 | 1.05864252669039 | 1.85126919291586 | 2.45067668462875e-19 | 2.27069108929246e-18 |
| MIR3125 | 0.48200545112782 | 1.52719733096085 | 1.6637651190178 | 0.00800714210517191 | 0.0110900160367032 |
| MMP7 | 16.5903137218045 | 74.054003202847 | 2.15823655884035 | 5.8679781532351e-08 | 1.53152726782863e-07 |
| MYOC | 2.12216390977444 | 0.129089323843416 | -4.03909449556495 | 0.0100753278679853 | 0.0137598534196358 |
| FZD9 | 0.299652443609023 | 1.23616637010676 | 2.04451087821267 | 1.34013171908998e-21 | 1.60736276713234e-20 |
| AMTN | 0.113599248120301 | 2.07867259786477 | 4.19363735262991 | 2.17859580981259e-16 | 1.4388402352274e-15 |
| S100A9 | 118.16959924812 | 560.915132206406 | 2.24692357401292 | 1.81782290874783e-25 | 3.43575321121617e-24 |
| SNORA74B | 18.4528454887218 | 1.91716975088968 | -3.2667933141874 | 0.0367110738854491 | 0.0460235275950799 |
| KRT6A | 4.26236842105263 | 26.1854044483986 | 2.61903568413641 | 5.31977039663654e-15 | 3.01998399370149e-14 |
| CXCL17 | 11.2365827067669 | 42.7492450177936 | 1.92769559439431 | 8.78773604918623e-10 | 2.81077913632243e-09 |
| NXPH4 | 1.64244492481203 | 5.4634090747331 | 1.73395645512192 | 1.01118324720427e-21 | 1.22970578067462e-20 |
| AL031668.2 | 0.763786466165414 | 3.18316423487544 | 2.05922033291044 | 3.13794060687141e-05 | 5.98375720989481e-05 |
| C4BPA | 0.231077443609023 | 1.95143007117438 | 3.07808342064879 | 0.00550662255496665 | 0.00779972100016135 |
| GFRA3 | 0.242935526315789 | 1.65714306049822 | 2.77005276840321 | 3.77309161368126e-08 | 1.00906099419983e-07 |
| SBSN | 0.380331578947368 | 5.90161014234875 | 3.95577898406117 | 8.09270446455146e-32 | 3.2152611428926e-30 |
| NFE4 | 0.190629511278195 | 3.27245747330961 | 4.10153105929206 | 4.63345635835964e-05 | 8.64756113512349e-05 |
| AC245884.9 | 0.204237969924812 | 1.1152140569395 | 2.44899764316519 | 1.9373636093533e-05 | 3.78665389557695e-05 |
| PLIN5 | 3.47588082706767 | 1.11742402135231 | -1.63720187975498 | 1.49319675949437e-48 | 4.04682986050822e-46 |
| ERICH5 | 0.245487030075188 | 0.848923309608541 | 1.78998742486431 | 4.37431924128326e-14 | 2.24323306232322e-13 |
| ZIC1 | 0.271409586466165 | 0.943374199288256 | 1.79735846544173 | 2.68453714071633e-13 | 1.26807407982109e-12 |
| DEFB1 | 3.58266936090226 | 25.3940610320285 | 2.82538431933271 | 9.03869547687889e-12 | 3.60336961525062e-11 |
| CASC8 | 0.18693984962406 | 1.02020035587189 | 2.448206465468 | 4.47337970342202e-16 | 2.84545195971651e-15 |
| CLDN6 | 0.269637781954887 | 2.55768612099644 | 3.24574465980225 | 3.91392894943981e-11 | 1.46201574367827e-10 |

**Appendix 8**

**3 risk PRGs**

**Table 6. 3 risk PRGs.**

| id | CEL | WT1 | ULBP2 | riskScore | risk |
| --- | --- | --- | --- | --- | --- |
| TCGA-D8-A1XO | 0.035215104 | 0 | 0.016303685 | 0.014948378 | low |
| TCGA-AN-A0FN | 0.04975195 | 0.323088068 | 0.962958442 | 0.304270843 | high |
| TCGA-AC-A62X | 0.541928459 | 2.355240087 | 0.493884775 | 0.838516258 | high |
| TCGA-E2-A14U | 1.141013114 | 0 | 0.138019589 | 0.398393359 | high |
| TCGA-E9-A1R3 | 2.83291231 | 0 | 0.56592033 | 1.03830371 | high |
| TCGA-BH-A18M | 0.563210322 | 0 | 0.276309148 | 0.242502429 | high |
| TCGA-E2-A15T | 0.428527875 | 0 | 0 | 0.138206628 | low |
| TCGA-AR-A0TU | 0.118100885 | 0 | 0.850664274 | 0.225453074 | high |
| TCGA-EW-A1IX | 0.0594333 | 0 | 0.075296194 | 0.035752552 | low |
| TCGA-B6-A408 | 1.666453004 | 0 | 0 | 0.537455935 | high |
| TCGA-E2-A1IE | 0.112689207 | 0 | 0 | 0.036343949 | low |
| TCGA-E2-A10E | 0.007855179 | 1.411340465 | 0.124008507 | 0.362395185 | high |
| TCGA-EW-A3U0 | 0.169082795 | 0.080958175 | 0.624409535 | 0.211137403 | high |
| TCGA-BH-A0H5 | 0 | 0.19699596 | 0 | 0.046417323 | low |
| TCGA-E2-A14W | 0.238038775 | 0 | 0.057300573 | 0.089391839 | low |
| TCGA-AQ-A04L | 0.047735911 | 0 | 0.205116553 | 0.060573662 | low |
| TCGA-BH-A1FD | 0.016633409 | 1.863225991 | 0.413127019 | 0.535382209 | high |
| TCGA-AR-A0TR | 0.222123125 | 0 | 0 | 0.071638019 | low |
| TCGA-A7-A0CE | 0.52303108 | 0 | 1.27435589 | 0.449369652 | high |
| TCGA-AO-A0J8 | 0.282878207 | 0 | 0.080766295 | 0.109021687 | low |
| TCGA-BH-A1F0 | 0.185327101 | 0 | 0.836790279 | 0.244078702 | high |
| TCGA-A2-A0SU | 0.693567661 | 0 | 0 | 0.22368591 | high |
| TCGA-AC-A2FF | 0.08563846 | 0 | 0 | 0.02761968 | low |
| TCGA-LL-A6FP | 0.076148358 | 0 | 0 | 0.024558981 | low |
| TCGA-E2-A15S | 0.2587291 | 0 | 0 | 0.083443992 | low |
| TCGA-E2-A14N | 0.480990638 | 1.019158949 | 0.116067583 | 0.42083132 | high |
| TCGA-D8-A1JK | 0.051173182 | 0 | 0.737347044 | 0.17890911 | low |
| TCGA-D8-A3Z6 | 0.371850032 | 0 | 0.478483777 | 0.225316027 | high |
| TCGA-A8-A06U | 0.15449774 | 0 | 0.022851163 | 0.054860926 | low |
| TCGA-A7-A13E | 0.137577245 | 0 | 0.441231064 | 0.141554433 | low |
| TCGA-UL-AAZ6 | 2.478765484 | 0 | 1.296270722 | 1.084950022 | high |
| TCGA-B6-A0I9 | 0.205486588 | 0 | 1.221843878 | 0.335390743 | high |
| TCGA-A8-A099 | 0.006508898 | 0 | 0.00567215 | 0.003348541 | low |
| TCGA-OL-A5RY | 0.0594333 | 0.686304931 | 1.933013437 | 0.606636755 | high |
| TCGA-E9-A1NA | 0.931488768 | 0 | 1.643131043 | 0.662328228 | high |
| TCGA-D8-A1XV | 0.6787974 | 3.956738 | 0 | 1.151231692 | high |
| TCGA-BH-A18T | 0.371664397 | 0 | 0 | 0.119867308 | low |
| TCGA-B6-A0RT | 0.216287638 | 0 | 1.401486652 | 0.378441613 | high |
| TCGA-A1-A0SJ | 0 | 0 | 0.021719791 | 0.004783911 | low |
| TCGA-E9-A247 | 0.149233736 | 0 | 0.777075908 | 0.219285615 | high |
| TCGA-C8-A12P | 4.167696885 | 0 | 1.447571419 | 1.662980438 | high |
| TCGA-A8-A09X | 7.019481866 | 0 | 0.531588704 | 2.380973059 | high |
| TCGA-BH-A1FC | 0.83026995 | 0.371119852 | 0.226596571 | 0.405129053 | high |
| TCGA-A8-A082 | 1.667530115 | 3.057606539 | 0.271988482 | 1.318161248 | high |
| TCGA-C8-A12M | 0.422289797 | 0 | 0.079430822 | 0.153689857 | low |
| TCGA-BH-A1EV | 0.624456038 | 0.275695731 | 0.588956247 | 0.396078453 | high |
| TCGA-C8-A135 | 0 | 0 | 2.556260226 | 0.563031254 | high |
| TCGA-WT-AB44 | 0.672458538 | 0.11822103 | 0 | 0.244733823 | high |
| TCGA-A7-A3J0 | 1.859409126 | 5.156172572 | 0 | 1.81461426 | high |
| TCGA-E9-A1R0 | 0.082636794 | 0 | 0.030624179 | 0.033396752 | low |
| TCGA-B6-A0IJ | 0.11291514 | 0 | 0 | 0.036416816 | low |
| TCGA-LL-A441 | 0.859313629 | 0 | 0.04010302 | 0.28597438 | high |
| TCGA-A2-A3XT | 0.713628128 | 0 | 0.406856508 | 0.319768233 | high |
| TCGA-D8-A27I | 0.261860341 | 0 | 0.023981764 | 0.089735987 | low |
| TCGA-A8-A094 | 0.180614292 | 0 | 0.551541708 | 0.179731086 | low |
| TCGA-A2-A1G6 | 0.250817508 | 0 | 0 | 0.080892385 | low |
| TCGA-BH-A0GY | 0.294050654 | 0 | 0.380927335 | 0.178737191 | low |
| TCGA-E2-A1B0 | 0 | 0 | 0.469769531 | 0.103469485 | low |
| TCGA-E9-A1RH | 0 | 1.7805314 | 0 | 0.419539071 | high |
| TCGA-C8-A1HG | 0.205802436 | 0 | 0.389514694 | 0.152167241 | low |
| TCGA-A1-A0SB | 0.287833451 | 0 | 0.334709838 | 0.166552377 | low |
| TCGA-A7-A0DC | 0.089385313 | 0 | 0 | 0.028828096 | low |
| TCGA-A7-A3IZ | 0.063309476 | 0 | 0 | 0.02041825 | low |
| TCGA-LL-A5YL | 0.253866058 | 0 | 0.791455709 | 0.256198333 | high |
| TCGA-BH-A0DI | 3.514266181 | 0 | 0.359108681 | 1.212499016 | high |
| TCGA-E2-A1B1 | 0.06834321 | 0 | 0.28438022 | 0.084678109 | low |
| TCGA-BH-A0H3 | 0.003566777 | 0 | 0 | 0.001150339 | low |
| TCGA-D8-A27K | 0.216705316 | 0 | 0.053233795 | 0.081615749 | low |
| TCGA-A2-A04U | 0.70925244 | 0 | 1.63962239 | 0.589880898 | high |
| TCGA-AO-A0J6 | 0.132201729 | 0.836449627 | 2.176884462 | 0.719197635 | high |
| TCGA-BH-A0E2 | 0.222227095 | 0.952992937 | 0.872579139 | 0.488411894 | high |
| TCGA-E2-A1L8 | 0.123714523 | 0 | 0 | 0.039899778 | low |
| TCGA-OL-A5RZ | 0.77860096 | 0 | 0.117611331 | 0.277014999 | high |
| TCGA-B6-A1KF | 1.313195669 | 1.572669892 | 2.265554954 | 1.293088442 | high |
| TCGA-E2-A1L7 | 0.18136519 | 1.90385049 | 2.22775094 | 0.997764366 | high |
| TCGA-B6-A0I6 | 0.500327529 | 0 | 1.728386068 | 0.542050219 | high |
| TCGA-AN-A0XL | 0.057196381 | 0 | 0.009856946 | 0.020617735 | low |
| TCGA-A8-A08B | 0.424253518 | 3.778044135 | 0 | 1.027032618 | high |
| TCGA-AC-A6IX | 2.326605955 | 0 | 0.657247784 | 0.895127768 | high |
| TCGA-E2-A2P6 | 0.470095359 | 0 | 0 | 0.151612761 | low |
| TCGA-E2-A3DX | 0.25862799 | 0 | 0.033748194 | 0.090844619 | low |
| TCGA-E2-A159 | 0.82297384 | 0 | 3.80345883 | 1.103155367 | high |
| TCGA-E9-A1NH | 0.160721564 | 0 | 0.812911724 | 0.230883658 | high |
| TCGA-A8-A0A1 | 0 | 0 | 0.20624219 | 0.045426048 | low |
| TCGA-D8-A4Z1 | 0.430748625 | 0 | 0 | 0.138922853 | low |
| TCGA-AR-A1AS | 0.067758878 | 0.167186624 | 0.40875011 | 0.151276325 | low |
| TCGA-AR-A24P | 0.204117037 | 0 | 0 | 0.065830787 | low |
| TCGA-A8-A09Z | 0.078701197 | 0 | 0.267222729 | 0.084239677 | low |
| TCGA-BH-A0HL | 1.988696282 | 0 | 0 | 0.641384196 | high |
| TCGA-A8-A08X | 1.357520854 | 0 | 1.563926136 | 0.782284583 | high |
| TCGA-A8-A09V | 0.207380543 | 0 | 0 | 0.066883317 | low |
| TCGA-D8-A1XT | 0.06096164 | 0 | 1.193337923 | 0.282500689 | high |
| TCGA-GM-A4E0 | 0.31988597 | 0 | 0 | 0.103167994 | low |
| TCGA-AN-A0XN | 0 | 1.97351517 | 1.187614924 | 0.726590146 | high |
| TCGA-A2-A04V | 0.0374896 | 0 | 0 | 0.012090955 | low |
| TCGA-A7-A26I | 0.895364898 | 0.569153706 | 1.378150068 | 0.72642141 | high |
| TCGA-A2-A3XS | 0.90326481 | 0 | 2.71539583 | 0.889398167 | high |
| TCGA-BH-A0BP | 0.14824439 | 1.713396631 | 0.935842598 | 0.657656216 | high |
| TCGA-AC-A3QQ | 0.01420087 | 0 | 0 | 0.004579992 | low |
| TCGA-AO-A12C | 0.363937408 | 0 | 0.349433413 | 0.194339991 | low |
| TCGA-E2-A1IH | 1.524447076 | 0.263391529 | 1.051920415 | 0.785410356 | high |
| TCGA-BH-A0DS | 0 | 0 | 0.274474238 | 0.060454555 | low |
| TCGA-AN-A0FS | 0.01115387 | 0 | 0 | 0.003597289 | low |
| TCGA-E2-A15F | 0.683055353 | 0 | 0.364539134 | 0.300587408 | high |
| TCGA-BH-A0H7 | 2.646531761 | 0 | 0.375042132 | 0.936151166 | high |
| TCGA-A8-A079 | 0.206644329 | 0 | 0 | 0.066645877 | low |
| TCGA-D8-A1XY | 0.443204081 | 0 | 0.339493757 | 0.217715407 | high |
| TCGA-AO-A0JD | 0.271409954 | 0 | 0.689846551 | 0.23947649 | high |
| TCGA-B6-A0WZ | 0.278003928 | 0 | 0 | 0.089660411 | low |
| TCGA-D8-A1XB | 0.26135581 | 0.04889735 | 0.35654097 | 0.174342852 | low |
| TCGA-BH-A0BS | 0.071610686 | 0 | 0 | 0.023095514 | low |
| TCGA-BH-A1EX | 0.587231632 | 0 | 2.533642054 | 0.747440424 | high |
| TCGA-D8-A1X8 | 0.124274574 | 0 | 0 | 0.040080403 | low |
| TCGA-HN-A2OB | 0.376574908 | 0.28689206 | 0 | 0.189050182 | low |
| TCGA-B6-A0I1 | 2.471160759 | 0.30896649 | 1.218850099 | 1.138245505 | high |
| TCGA-OL-A97C | 0.058491902 | 0.68867811 | 2.424821337 | 0.715215886 | high |
| TCGA-E2-A1LK | 0.402580662 | 0 | 1.381535696 | 0.434129576 | high |
| TCGA-D8-A1J8 | 0.110767231 | 0 | 1.668745909 | 0.403275122 | high |
| TCGA-A8-A06P | 0.219000184 | 0 | 0.163037815 | 0.106540856 | low |
| TCGA-D8-A3Z5 | 0.669796 | 0 | 0 | 0.216019195 | high |
| TCGA-E2-A152 | 0.094259011 | 0 | 0.914752168 | 0.231879447 | high |
| TCGA-BH-A0DT | 0.195975778 | 0 | 0 | 0.06320511 | low |
| TCGA-A1-A0SQ | 1.489891543 | 0 | 0 | 0.480512232 | high |
| TCGA-BH-A1FE | 0.42240854 | 1.813100498 | 1.018441142 | 0.787763854 | high |
| TCGA-BH-A0BR | 0 | 0.40472504 | 3.379514663 | 0.839721501 | high |
| TCGA-BH-A18V | 0.160075047 | 0 | 1.160268885 | 0.307182591 | high |
| TCGA-GM-A3XN | 0.47164506 | 0 | 0.16983788 | 0.189520348 | low |
| TCGA-A8-A09B | 0.17416046 | 0.13799129 | 0.578467749 | 0.21609455 | high |
| TCGA-PL-A8LY | 0.381559326 | 0 | 0 | 0.12305857 | low |
| TCGA-E2-A10A | 0.102254312 | 0 | 0.246594652 | 0.087292453 | low |
| TCGA-A7-A6VV | 1.01794706 | 0.7240862 | 1.78108352 | 0.891210519 | high |
| TCGA-BH-A0BW | 0.17416046 | 0 | 1.167679944 | 0.313357676 | high |
| TCGA-AN-A04A | 1.598002 | 0 | 1.534759 | 0.853419105 | high |
| TCGA-OL-A66P | 0.661477864 | 0 | 0.450552241 | 0.312573236 | high |
| TCGA-A2-A0SV | 1.160035 | 1.265578 | 1.037591 | 0.900866844 | high |
| TCGA-E2-A1LG | 0.242653403 | 0 | 1.628143914 | 0.436867552 | high |
| TCGA-E2-A1LL | 0.138863768 | 0 | 3.124969668 | 0.733078467 | high |
| TCGA-A2-A0ET | 0.069861259 | 0 | 0 | 0.022531297 | low |
| TCGA-B6-A0RI | 0.279995607 | 0 | 0.584123633 | 0.218959401 | high |
| TCGA-BH-A0GZ | 0.679091524 | 0 | 0.768997357 | 0.388393302 | high |
| TCGA-A2-A25A | 0.184792402 | 0 | 0.11689901 | 0.085345994 | low |
| TCGA-A8-A06N | 0.059903747 | 0 | 0 | 0.019319851 | low |
| TCGA-EW-A1J5 | 0.63460807 | 0 | 0 | 0.204670563 | high |
| TCGA-D8-A27V | 0.04690488 | 0 | 0 | 0.015127523 | low |
| TCGA-A2-A0ST | 0.250105163 | 0 | 0.34798758 | 0.157308942 | low |
| TCGA-A2-A0CT | 0.182544296 | 0 | 0.315645851 | 0.1283961 | low |
| TCGA-AR-A0TT | 0.242141463 | 0.581680662 | 0.984435351 | 0.431980821 | high |
| TCGA-LL-A8F5 | 0.3984941 | 1.015736 | 2.146488 | 0.840630384 | high |
| TCGA-AN-A0FW | 0.238449619 | 0 | 0.192683928 | 0.119343318 | low |
| TCGA-BH-A18U | 0.009322283 | 0.182936946 | 1.162879965 | 0.302242341 | high |
| TCGA-E9-A248 | 2.755168326 | 0 | 1.536729444 | 1.227056497 | high |
| TCGA-A2-A0CU | 0.169407486 | 0 | 0 | 0.05463644 | low |
| TCGA-BH-A0HY | 0.177929034 | 0.90102101 | 0.108446595 | 0.293574526 | high |
| TCGA-D8-A1XG | 0.410806116 | 4.927569896 | 0.835327147 | 1.477539201 | high |
| TCGA-BH-A204 | 1.909114774 | 0 | 0.124244966 | 0.643083653 | high |
| TCGA-AR-A0U4 | 0.031976153 | 0 | 3.827870395 | 0.853423611 | high |
| TCGA-E2-A14S | 0.334574841 | 0 | 0.081615583 | 0.125881683 | low |
| TCGA-D8-A1JS | 0.047735911 | 0 | 0 | 0.015395543 | low |
| TCGA-C8-A1HK | 0.525346169 | 0 | 0.481251263 | 0.275430374 | high |
| TCGA-AR-A250 | 0.114721191 | 0 | 0.45074649 | 0.136278843 | low |
| TCGA-EW-A1PB | 0.0122516 | 0 | 0.08851509 | 0.02344729 | low |
| TCGA-AC-A2B8 | 0.061196612 | 0.527357968 | 0.094303043 | 0.16476674 | low |
| TCGA-A8-A08I | 0.050581188 | 0.848597904 | 1.142515932 | 0.467910527 | high |
| TCGA-LL-A6FQ | 0.23937355 | 0 | 0 | 0.077201538 | low |
| TCGA-A1-A0SP | 0.144389862 | 0 | 3.252044767 | 0.762849748 | high |
| TCGA-LL-A442 | 1.669369591 | 0 | 0 | 0.538396578 | high |
| TCGA-A2-A3XU | 0.916593088 | 0.704480546 | 1.34061582 | 0.75688717 | high |
| TCGA-A2-A25B | 0.492474336 | 0 | 0.291877988 | 0.223118147 | high |
| TCGA-AO-A1KT | 0.269906712 | 0 | 0.335646907 | 0.160977131 | low |
| TCGA-B6-A1KN | 0.01456604 | 0 | 0.2431866 | 0.058261035 | low |
| TCGA-LL-A5YP | 0.181472421 | 0.055510443 | 3.116351356 | 0.75800185 | high |
| TCGA-AR-A1AU | 0.553057312 | 0 | 0.279542285 | 0.239940049 | high |
| TCGA-A7-A26J | 0.326102807 | 0 | 0.076712533 | 0.1220694 | low |
| TCGA-E9-A245 | 2.023485 | 0 | 0 | 0.652604076 | high |
| TCGA-BH-A42T | 0.339244725 | 0 | 0.036990948 | 0.117558953 | low |
| TCGA-A2-A0EM | 0 | 0 | 0.176151942 | 0.038798495 | low |
| TCGA-PE-A5DC | 1.847616177 | 0 | 0 | 0.595883759 | high |
| TCGA-A7-A13D | 0.263885856 | 0 | 0.827275077 | 0.267319293 | high |
| TCGA-BH-A0HK | 0.575991346 | 0 | 0.587972966 | 0.315270277 | high |
| TCGA-B6-A0IP | 0.135759006 | 0 | 0.131323148 | 0.072708994 | low |
| TCGA-D8-A1XA | 0.155701119 | 0 | 0 | 0.050215932 | low |
| TCGA-D8-A1X5 | 0.020879581 | 0 | 0.324052596 | 0.078108454 | low |
| TCGA-D8-A27L | 0.35531929 | 0 | 0.2740422 | 0.174955164 | low |
| TCGA-D8-A1JJ | 0.19449013 | 0.28472675 | 2.17485692 | 0.608839871 | high |
| TCGA-A2-A1G1 | 0 | 0.711970148 | 3.252494069 | 0.884139336 | high |
| TCGA-E2-A1IL | 0.099176153 | 0 | 0 | 0.031985788 | low |
| TCGA-A7-A4SB | 0.124834388 | 0 | 0 | 0.040260951 | low |
| TCGA-A7-A13H | 0.14604318 | 0 | 0.12306233 | 0.0742063 | low |
| TCGA-BH-A0AY | 0.381282908 | 0.779405686 | 0 | 0.306617481 | high |
| TCGA-A8-A075 | 0.3365782 | 0.3445073 | 0.7101569 | 0.34614249 | high |
| TCGA-E2-A158 | 1.922749883 | 0 | 0.126725763 | 0.648027589 | high |
| TCGA-BH-A0B4 | 0.069044065 | 0 | 0 | 0.02226774 | low |
| TCGA-BH-A0DV | 0 | 0 | 0.382041145 | 0.084146795 | low |
| TCGA-A2-A0EP | 0.20137366 | 0.418227332 | 0.382951889 | 0.247838533 | high |
| TCGA-EW-A1PE | 0.131645037 | 0 | 0.338558857 | 0.117027054 | low |
| TCGA-LL-A73Z | 1.28471594 | 0 | 3.60508128 | 1.208380262 | high |
| TCGA-AR-A1AJ | 1.406768541 | 0.380445299 | 0.204553449 | 0.588400632 | high |
| TCGA-E9-A227 | 0.981810981 | 0 | 0 | 0.316648677 | high |
| TCGA-AO-A0JB | 1.205041875 | 0 | 0 | 0.388643968 | high |
| TCGA-A8-A09N | 0.06495048 | 0 | 0.30740809 | 0.088655926 | low |
| TCGA-E2-A15A | 0.406084571 | 0 | 0.150997549 | 0.16422642 | low |
| TCGA-A8-A08A | 0.290402836 | 0 | 0 | 0.093659244 | low |
| TCGA-AC-A5XS | 0.271610257 | 0 | 0.031374478 | 0.094508769 | low |
| TCGA-BH-A209 | 0.213988039 | 3.841657339 | 0 | 0.974207776 | high |
| TCGA-AR-A24S | 0 | 1.341103815 | 0.244836538 | 0.369925289 | high |
| TCGA-A7-A26E | 0.130935726 | 0.08695694 | 0 | 0.062718019 | low |
| TCGA-EW-A1P6 | 0.164745918 | 0 | 0 | 0.053133014 | low |
| TCGA-C8-A1HM | 0.319982509 | 0.003515469 | 0 | 0.104027464 | low |
| TCGA-OL-A6VQ | 0.149013945 | 0 | 0.066139124 | 0.062626747 | low |
| TCGA-E2-A1B5 | 0.092654116 | 0 | 0.204215494 | 0.07486199 | low |
| TCGA-A2-A0CV | 0.205697162 | 0 | 0 | 0.066340401 | low |
| TCGA-5L-AAT1 | 0.164637313 | 1.628924724 | 0.502473706 | 0.54758739 | high |
| TCGA-B6-A0X4 | 1.163704842 | 0 | 0 | 0.375312159 | high |
| TCGA-E2-A105 | 1.66352995 | 0 | 0 | 0.536513207 | high |
| TCGA-AC-A3HN | 0.290007872 | 0 | 0.184381501 | 0.134142965 | low |
| TCGA-A1-A0SO | 0.344087711 | 0 | 0 | 0.110973416 | low |
| TCGA-E2-A10B | 0.11787584 | 0 | 0.07188346 | 0.053849468 | low |
| TCGA-A1-A0SH | 0 | 0 | 0.104144369 | 0.022938406 | low |
| TCGA-E9-A2JS | 0.421128 | 0 | 0.08766932 | 0.155129738 | low |
| TCGA-A2-A04R | 0 | 0 | 0 | 0 | low |
| TCGA-C8-A12Q | 1.408672 | 0 | 0.2525146 | 0.509935535 | high |
| TCGA-AN-A0FT | 0.135981017 | 0 | 0.178556446 | 0.083184006 | low |
| TCGA-4H-AAAK | 0.20601296 | 0 | 0.023856179 | 0.071696713 | low |
| TCGA-E2-A15M | 0.094259011 | 1.057132308 | 0.612293886 | 0.41434885 | high |
| TCGA-A8-A08H | 0.085407805 | 0 | 0.866995208 | 0.218506052 | high |
| TCGA-C8-A273 | 0.468457585 | 0 | 0.58833059 | 0.280667805 | high |
| TCGA-BH-A0DE | 0.069394353 | 0 | 0.390622761 | 0.108417659 | low |
| TCGA-C8-A12U | 0.775256418 | 0 | 2.862924864 | 0.880607684 | high |
| TCGA-A2-A0EO | 0 | 0 | 0.01756482 | 0.003868754 | low |
| TCGA-AC-A6IW | 0.174699488 | 0.501546149 | 0.117017752 | 0.200294227 | high |
| TCGA-LL-A50Y | 0.252952233 | 0 | 0.198007443 | 0.125193162 | low |
| TCGA-A7-A0DA | 0.743507873 | 0 | 1.132626009 | 0.48925987 | high |
| TCGA-GM-A2DL | 0.265084786 | 0 | 0.291664235 | 0.149734547 | low |
| TCGA-E9-A244 | 0.030654295 | 0 | 1.038750086 | 0.238677244 | high |
| TCGA-E2-A15C | 0.352214228 | 0 | 0.112618656 | 0.138399256 | low |
| TCGA-BH-A0BT | 0.057785412 | 0 | 0.047176811 | 0.029027625 | low |
| TCGA-BH-A0E9 | 0.225757229 | 0 | 0.087669322 | 0.092119752 | low |
| TCGA-BH-A18I | 0.018576213 | 0 | 0.80962879 | 0.184316578 | low |
| TCGA-BH-A0HI | 0.059786151 | 0 | 0.04320927 | 0.028799019 | low |
| TCGA-A2-A0CZ | 0.035215104 | 0 | 0.413027672 | 0.102329155 | low |
| TCGA-D8-A1X9 | 0.212941439 | 0 | 0.712149452 | 0.225531867 | high |
| TCGA-BH-A18R | 0.068576869 | 0 | 0.030999371 | 0.028944855 | low |
| TCGA-AO-A12B | 2.484440375 | 4.220115904 | 0 | 1.795637088 | high |
| TCGA-BH-A0B3 | 0.4881478 | 0 | 0.393942532 | 0.244203087 | high |
| TCGA-C8-A8HP | 4.335431144 | 0 | 0.320589969 | 1.468852986 | high |
| TCGA-AR-A1AM | 0.381835687 | 0 | 0.397957619 | 0.210800192 | high |
| TCGA-A2-A4S0 | 1.026390681 | 4.02115795 | 0 | 1.278514707 | high |
| TCGA-AO-A126 | 0.226068258 | 0 | 0 | 0.072910383 | low |
| TCGA-AR-A5QN | 0.3128195 | 0 | 0.3939425 | 0.18765709 | low |
| TCGA-A8-A08F | 0.313789711 | 0 | 0.194498105 | 0.144041204 | low |
| TCGA-PE-A5DE | 0.277804594 | 0 | 0.177411878 | 0.128672126 | low |
| TCGA-A8-A076 | 0.71076046 | 0 | 2.29226471 | 0.734115542 | high |
| TCGA-A2-A3XZ | 0.716489566 | 0 | 1.557405524 | 0.574106234 | high |
| TCGA-AR-A256 | 0.06049157 | 0 | 0.771481095 | 0.189432653 | low |
| TCGA-BH-A0DG | 0.094946226 | 0 | 0.293800526 | 0.095332856 | low |
| TCGA-C8-A26Z | 1.035341735 | 0 | 0.831625062 | 0.517083423 | high |
| TCGA-BH-A0HW | 0.1481344 | 0 | 0 | 0.047775552 | low |
| TCGA-A1-A0SD | 1.002507352 | 0 | 0.535284747 | 0.441223159 | high |
| TCGA-A2-A0CX | 0.052001527 | 0 | 0.35366041 | 0.09466704 | low |
| TCGA-AO-A03R | 0.197459752 | 1.204545897 | 0 | 0.34750576 | high |
| TCGA-PL-A8LZ | 0.304543297 | 0 | 0.969295705 | 0.311712797 | high |
| TCGA-BH-A1F8 | 0.580514813 | 0 | 0.064546751 | 0.20144148 | high |
| TCGA-BH-A0BZ | 0.639238237 | 0 | 0.806731294 | 0.383851143 | high |
| TCGA-AC-A8OP | 0.245312287 | 0 | 0 | 0.079116869 | low |
| TCGA-GI-A2C9 | 0.34730651 | 0 | 2.16260477 | 0.588337866 | high |
| TCGA-AO-A125 | 0.266895076 | 0.083253957 | 0 | 0.105694418 | low |
| TCGA-EW-A1OZ | 0.07009465 | 0 | 0 | 0.02260657 | low |
| TCGA-BH-A0C0 | 0.387259031 | 2.060579219 | 0.645876512 | 0.752680438 | high |
| TCGA-AN-A03Y | 0.02269519 | 2.47939697 | 0 | 0.591529341 | high |
| TCGA-D8-A145 | 0.297197404 | 0 | 0.284594911 | 0.158534285 | low |
| TCGA-OL-A66I | 0.822776091 | 0.128568299 | 1.354199036 | 0.59392181 | high |
| TCGA-OL-A5S0 | 0.23268612 | 0.35462892 | 0.29230541 | 0.222986427 | high |
| TCGA-B6-A0I5 | 1.3236062 | 0 | 0.044574182 | 0.436700454 | high |
| TCGA-Z7-A8R6 | 0.30307739 | 0 | 0 | 0.097746976 | low |
| TCGA-LD-A9QF | 1.60205996 | 0 | 0.894505949 | 0.713708382 | high |
| TCGA-C8-A134 | 0.168649749 | 0 | 0.961745198 | 0.266222059 | high |
| TCGA-AN-A0XT | 0.522286085 | 0 | 0.034372293 | 0.176015746 | low |
| TCGA-BH-A0HA | 0.327682816 | 0 | 0.514012765 | 0.218896908 | high |
| TCGA-AR-A251 | 0.084023028 | 0 | 1.365378131 | 0.327831192 | high |
| TCGA-C8-A26V | 0.150771243 | 1.946452872 | 2.149214235 | 0.980637413 | high |
| TCGA-OL-A5D7 | 1.726585076 | 0 | 0.817123125 | 0.736825569 | high |
| TCGA-E2-A1IU | 0 | 0 | 0.104144369 | 0.022938406 | low |
| TCGA-AN-A0XO | 2.067545945 | 0.111080648 | 1.200340374 | 0.957369833 | high |
| TCGA-AN-A0AR | 0.632096427 | 0.299151155 | 2.358218865 | 0.793759726 | high |
| TCGA-E2-A1IO | 0.259638952 | 0 | 0.251967327 | 0.139234709 | low |
| TCGA-D8-A27H | 0.1745917 | 0 | 2.967721812 | 0.709966499 | high |
| TCGA-A7-A56D | 0.034975454 | 0.216520535 | 0.688584647 | 0.213962714 | high |
| TCGA-GM-A3XL | 2.33130378 | 0 | 0 | 0.751880222 | high |
| TCGA-BH-A0DX | 0.14659383 | 0.410159333 | 0.637913112 | 0.284426892 | high |
| TCGA-A2-A4RX | 0.179111222 | 0.076741663 | 3.573910106 | 0.863022939 | high |
| TCGA-B6-A0X1 | 0.046548564 | 0 | 0.561383587 | 0.138660622 | low |
| TCGA-S3-AA0Z | 1.615134852 | 0 | 0.776836516 | 0.692007849 | high |
| TCGA-BH-A1FH | 0.743507873 | 0 | 0.112261461 | 0.264518615 | high |
| TCGA-AR-A24W | 0.05164659 | 0 | 0.06221669 | 0.030360385 | low |
| TCGA-A8-A06T | 0.15142963 | 0 | 0 | 0.048838313 | low |
| TCGA-B6-A0RM | 0.413056898 | 2.851624515 | 0 | 0.805133211 | high |
| TCGA-A2-A04N | 0.148134417 | 0 | 0.230038794 | 0.098442946 | low |
| TCGA-E9-A226 | 0 | 0 | 0.39384203 | 0.086746009 | low |
| TCGA-BH-A0BJ | 0.035933791 | 0 | 0 | 0.011589183 | low |
| TCGA-E2-A10C | 0.856939027 | 0 | 0.242416087 | 0.32976917 | high |
| TCGA-BH-A5IZ | 0.117875841 | 0 | 2.99866088 | 0.698489299 | high |
| TCGA-E9-A1RD | 0.137866606 | 0 | 0.102827573 | 0.067112409 | low |
| TCGA-AC-A5XU | 0.11978749 | 0 | 0.26189927 | 0.096318096 | low |
| TCGA-E2-A576 | 0.826528345 | 0 | 0 | 0.266567712 | high |
| TCGA-E9-A295 | 0.091506564 | 0 | 0.040600411 | 0.038454709 | low |
| TCGA-LD-A74U | 0.320465098 | 0.092661686 | 0.15528179 | 0.159389973 | low |
| TCGA-E2-A570 | 0.749863313 | 0 | 0 | 0.241842097 | high |
| TCGA-E9-A1NF | 1.325633461 | 0 | 1.229092092 | 0.698251268 | high |
| TCGA-E9-A1R4 | 0.309709819 | 0 | 0 | 0.099886033 | low |
| TCGA-B6-A409 | 0.10566608 | 0 | 1.114361776 | 0.27952358 | high |
| TCGA-EW-A423 | 0.445926804 | 0 | 0.141182186 | 0.174914243 | low |
| TCGA-OL-A6VO | 0.9269281 | 0 | 1.617596 | 0.655233102 | high |
| TCGA-EW-A6SD | 0.072542764 | 0 | 0.687574433 | 0.17483841 | low |
| TCGA-GM-A2DH | 2.246853569 | 0.75301401 | 0.466886651 | 1.004907784 | high |
| TCGA-LL-A5YM | 0.02933111 | 0 | 0.2265966 | 0.059368946 | low |
| TCGA-BH-A0H6 | 0.317276739 | 0 | 0.120339068 | 0.128831861 | low |
| TCGA-E9-A1N8 | 0.70298647 | 0 | 0.88742824 | 0.422184872 | high |
| TCGA-AR-A24Z | 0.116975282 | 0.746753055 | 0.323633268 | 0.28496265 | high |
| TCGA-A7-A4SD | 2.12986906 | 0 | 0.562564334 | 0.810822604 | high |
| TCGA-AR-A0U0 | 1.7060687 | 0 | 2.38806286 | 1.076217389 | high |
| TCGA-BH-A203 | 1.347597149 | 1.291976987 | 3.059790147 | 1.412979895 | high |
| TCGA-E2-A1IK | 0.919965446 | 4.206786765 | 0 | 1.287929913 | high |
| TCGA-D8-A13Y | 0.22658648 | 0 | 3.55172931 | 0.855366655 | high |
| TCGA-AR-A1AX | 0.101343014 | 0.201180509 | 0 | 0.080087944 | low |
| TCGA-E9-A1N9 | 0.138974517 | 0.196038131 | 0 | 0.091012988 | low |
| TCGA-C8-A1HN | 0.245312287 | 0 | 0.242746357 | 0.132583173 | low |
| TCGA-A2-A0YC | 0.032336429 | 0 | 0.26048405 | 0.067802115 | low |
| TCGA-A2-A0CL | 0.073008557 | 0 | 0.49237003 | 0.131993724 | low |
| TCGA-D8-A1JA | 2.162932 | 0 | 3.77294 | 1.528589897 | high |
| TCGA-D8-A1XM | 0.002584608 | 0.849432718 | 0.226596571 | 0.250891026 | high |
| TCGA-A2-A04X | 0.214824721 | 0 | 0.898515659 | 0.2671875 | high |
| TCGA-AO-A12A | 0.381559326 | 0 | 0.262878325 | 0.180959058 | low |
| TCGA-E2-A1BC | 0.132424341 | 0 | 0.070174458 | 0.058165158 | low |
| TCGA-A2-A25F | 0.03437614 | 1.080274 | 0.359006 | 0.344700373 | high |
| TCGA-EW-A2FV | 0.164094154 | 0 | 0.662117261 | 0.198758007 | high |
| TCGA-C8-A132 | 1.567145768 | 0 | 2.377687496 | 1.029127438 | high |
| TCGA-AN-A0AT | 0.014079125 | 0 | 0.241094353 | 0.057643168 | low |
| TCGA-AC-A3QP | 0.232479813 | 0 | 0.135085877 | 0.104731659 | low |
| TCGA-E9-A1R6 | 0.251732813 | 0 | 0.085855516 | 0.100097764 | low |
| TCGA-C8-A27B | 0.49982228 | 0 | 0.30772568 | 0.228978516 | high |
| TCGA-E2-A14P | 3.54466472 | 0 | 0.97397968 | 1.357731929 | high |
| TCGA-LL-A7SZ | 0.09620519 | 0 | 0 | 0.031027608 | low |
| TCGA-BH-A0BO | 0.133981568 | 0 | 0.381129902 | 0.127157141 | low |
| TCGA-BH-A0HX | 0.196611971 | 0 | 0.573875026 | 0.18980962 | low |
| TCGA-A1-A0SF | 0.042980092 | 0.152034401 | 0 | 0.049684942 | low |
| TCGA-BH-A0B9 | 0.161810439 | 0 | 1.065859043 | 0.286947962 | high |
| TCGA-D8-A1JG | 1.33684 | 0 | 0.9773779 | 0.646424023 | high |
| TCGA-D8-A1JN | 0.694076545 | 0 | 0.439246733 | 0.320596692 | high |
| TCGA-A8-A06Z | 0 | 0 | 0 | 0 | low |
| TCGA-A2-A0YD | 0.013713823 | 0.487940373 | 0.428552847 | 0.213785504 | high |
| TCGA-D8-A1JB | 1.359759879 | 0 | 0 | 0.438542831 | high |
| TCGA-JL-A3YX | 1.310830545 | 1.797068946 | 0.181757045 | 0.886231185 | high |
| TCGA-A1-A0SK | 0.854495785 | 0 | 0.976741285 | 0.490720605 | high |
| TCGA-AO-A03P | 0.234953358 | 0 | 0.024484006 | 0.081168706 | low |
| TCGA-E2-A1LA | 0.243267467 | 0 | 0 | 0.078457384 | low |
| TCGA-AN-A0FJ | 0.00809981 | 0 | 1.5399582 | 0.341797103 | high |
| TCGA-LL-A7T0 | 1.297608018 | 0 | 0 | 0.418497929 | high |
| TCGA-D8-A141 | 0.323453214 | 0 | 0.483347663 | 0.210778631 | high |
| TCGA-BH-A0HB | 0.280592524 | 0 | 0.212980032 | 0.137405368 | low |
| TCGA-C8-A12Z | 3.879363678 | 2.735352562 | 3.026771091 | 2.562336173 | high |
| TCGA-A8-A08S | 0.844612444 | 0 | 0.06246211 | 0.286157748 | high |
| TCGA-S3-AA10 | 0.263574309 | 0 | 1.042902542 | 0.314712023 | high |
| TCGA-E9-A22G | 0.708605616 | 0 | 1.483884061 | 0.555370011 | high |
| TCGA-C8-A1HE | 0.07184377 | 0 | 0.2328096 | 0.074448362 | low |
| TCGA-E2-A2P5 | 0.146043183 | 0 | 0 | 0.047101104 | low |
| TCGA-AO-A0J9 | 0.30883391 | 0 | 0.13144086 | 0.128554157 | low |
| TCGA-AR-A1AK | 0.116074108 | 0 | 0.245496049 | 0.09150757 | low |
| TCGA-C8-A27A | 0.12057383 | 0 | 0 | 0.038886858 | low |
| TCGA-E9-A5UP | 0.429594302 | 0 | 0 | 0.138550566 | low |
| TCGA-A2-A0T2 | 0.100431085 | 0.131301057 | 0.264508809 | 0.121588045 | low |
| TCGA-BH-A0C3 | 0.080901949 | 0 | 0 | 0.026092085 | low |
| TCGA-E2-A1IG | 0.144389862 | 0 | 1.136871762 | 0.296970533 | high |
| TCGA-C8-A1HL | 0.417546965 | 0 | 0.606733302 | 0.268301676 | high |
| TCGA-BH-A208 | 0 | 0.133657849 | 1.771904658 | 0.421765589 | high |
| TCGA-A2-A3KD | 0.804387811 | 4.292437068 | 0 | 1.270835821 | high |
| TCGA-A2-A0YK | 1.68967529 | 0 | 0 | 0.544945469 | high |
| TCGA-BH-A1FR | 0.544210621 | 3.367841437 | 0 | 0.969066248 | high |
| TCGA-BH-A18J | 0.17932605 | 2.31384419 | 1.28649473 | 0.886394611 | high |
| TCGA-B6-A0WY | 0.302784013 | 0 | 0.63756601 | 0.238079997 | high |
| TCGA-AR-A24M | 0 | 0 | 0.370155795 | 0.081528977 | low |
| TCGA-E9-A1NI | 0.366455981 | 0 | 0 | 0.118187517 | low |
| TCGA-A8-A093 | 0.05896269 | 0 | 0 | 0.019016346 | low |
| TCGA-AC-A6NO | 0.698213023 | 0 | 0.082706886 | 0.243400783 | high |
| TCGA-A8-A09M | 1.486230226 | 0 | 0 | 0.479331403 | high |
| TCGA-EW-A1OV | 0.581385979 | 0 | 0.175005715 | 0.226051676 | high |
| TCGA-C8-A278 | 0.182544296 | 0 | 0.929885632 | 0.263686001 | high |
| TCGA-AR-A1AP | 0 | 0 | 0.574055372 | 0.126439051 | low |
| TCGA-E2-A1LS | 0.054247271 | 0 | 0.109401133 | 0.041591792 | low |
| TCGA-A8-A09G | 0.331707542 | 0 | 0.350568542 | 0.184195397 | low |
| TCGA-A8-A09R | 1.688923719 | 0 | 0.587436385 | 0.674089373 | high |
| TCGA-GM-A5PX | 0.064950477 | 0 | 0.246594652 | 0.07526141 | low |
| TCGA-E2-A1BD | 0.404664475 | 0.678341196 | 0.27738756 | 0.351441219 | high |
| TCGA-E9-A1R2 | 0.786552753 | 0 | 0 | 0.253674988 | high |
| TCGA-AC-A62Y | 0.3105851 | 2.68146 | 0.2292621 | 0.782485702 | high |
| TCGA-AC-A2FO | 0.07475381 | 0 | 0 | 0.024109218 | low |
| TCGA-AR-A1AY | 0.55386597 | 1.49899773 | 0.813302117 | 0.710967061 | high |
| TCGA-AR-A2LN | 0.492982408 | 0 | 0 | 0.158994175 | low |
| TCGA-D8-A27W | 0.07800546 | 0 | 1.026642 | 0.251281824 | high |
| TCGA-OL-A66O | 0.40837095 | 0 | 0 | 0.131705719 | low |
| TCGA-BH-A0HU | 0.35926064 | 0 | 0.187570903 | 0.157180499 | low |
| TCGA-AR-A1AH | 0.302686206 | 0.139598214 | 0.670731493 | 0.27824628 | high |
| TCGA-A8-A081 | 2.963123566 | 3.645860337 | 0 | 1.814710146 | high |
| TCGA-C8-A8HQ | 0.247149916 | 0 | 0.497004579 | 0.189177695 | low |
| TCGA-A2-A04Y | 1.419657825 | 0 | 0.076269974 | 0.474659719 | high |
| TCGA-GM-A2DB | 0.372128435 | 0 | 1.144947269 | 0.372198295 | high |
| TCGA-E9-A3Q9 | 0.05294757 | 0 | 0 | 0.017076381 | low |
| TCGA-A8-A09I | 0.018576213 | 0 | 1.360443891 | 0.305636823 | high |
| TCGA-A8-A07L | 0.213255506 | 0 | 0.436506246 | 0.164921131 | low |
| TCGA-A2-A3XV | 0.289514 | 0 | 0.665534184 | 0.239960374 | high |
| TCGA-AR-A0TY | 0.059786151 | 0 | 0 | 0.019281925 | low |
| TCGA-LL-A6FR | 0.547220287 | 0 | 1.951761592 | 0.606373597 | high |
| TCGA-A2-A0CW | 0.037369992 | 0 | 0.170757782 | 0.049662779 | low |
| TCGA-E2-A14O | 1.747506331 | 0 | 0 | 0.563596842 | high |
| TCGA-D8-A1J9 | 0.158322867 | 0 | 0.871599537 | 0.243036376 | high |
| TCGA-A2-A0CQ | 0.3618817 | 0 | 0 | 0.116712243 | low |
| TCGA-A8-A08Z | 0.665123496 | 0.274778679 | 0.930904189 | 0.484294266 | high |
| TCGA-AC-A2FE | 0.178896357 | 0 | 0.125308619 | 0.085296698 | low |
| TCGA-AO-A03N | 0 | 0.342741199 | 0.079187894 | 0.098200253 | low |
| TCGA-EW-A2FW | 0.206854718 | 0 | 0.27415022 | 0.127096918 | low |
| TCGA-AR-A2LL | 0.006753779 | 0.35364125 | 0 | 0.085505184 | low |
| TCGA-AR-A24Q | 0.014322603 | 0 | 1.408102244 | 0.314762001 | high |
| TCGA-AO-A0J3 | 0.448468786 | 0 | 0 | 0.144637869 | low |
| TCGA-AR-A2LK | 0.732541032 | 0 | 0 | 0.236255403 | high |
| TCGA-A8-A08J | 4.503719106 | 0 | 0.686142244 | 1.603643387 | high |
| TCGA-A2-A0YF | 0.049277865 | 0 | 0 | 0.015892846 | low |
| TCGA-BH-A0AZ | 0.019667784 | 0 | 0.54678214 | 0.126775115 | low |
| TCGA-E9-A22B | 0.198412854 | 0 | 0.858128067 | 0.252998825 | high |
| TCGA-AC-A3W5 | 0.076380639 | 0.495974061 | 0.82039129 | 0.322194138 | high |
| TCGA-BH-A0C1 | 0.01663341 | 1.82098554 | 0 | 0.434435631 | high |
| TCGA-AN-A0XU | 0.328546292 | 0 | 2.904679191 | 0.745733644 | high |
| TCGA-B6-A0RQ | 0.250206951 | 0 | 0.010490087 | 0.083005974 | low |
| TCGA-D8-A1JL | 1.140236137 | 0.853934067 | 1.962503364 | 1.001204853 | high |
| TCGA-D8-A1Y0 | 0.087827671 | 0 | 0 | 0.028325733 | low |
| TCGA-E9-A229 | 0.323068031 | 0 | 0.450552241 | 0.20343102 | high |
| TCGA-A8-A07I | 0.1895969 | 0 | 0.848296276 | 0.247990039 | high |
| TCGA-OL-A5RU | 0.197777529 | 0 | 0.297958405 | 0.129413281 | low |
| TCGA-AR-A5QP | 0.33715009 | 2.02052248 | 0 | 0.584823082 | high |
| TCGA-D8-A1XZ | 0 | 0.419709627 | 0.255466653 | 0.155162425 | low |
| TCGA-EW-A1PC | 1.937009984 | 0 | 0.68555216 | 0.775711464 | high |
| TCGA-AO-A0J2 | 0.067408155 | 0 | 0.361979812 | 0.101468304 | low |
| TCGA-E2-A1LB | 0.059668545 | 0 | 0.389514694 | 0.105036883 | low |
| TCGA-5T-A9QA | 1.294615734 | 0.124461408 | 0 | 0.446859187 | high |
| TCGA-B6-A40C | 0.535959892 | 0.819134106 | 0.417492422 | 0.4578193 | high |
| TCGA-A7-A0CH | 0.188210732 | 0 | 0 | 0.060700767 | low |
| TCGA-E2-A9RU | 0.222331058 | 3.479823972 | 0.657235964 | 1.036401323 | high |
| TCGA-A2-A0D2 | 0.027765632 | 0 | 0.075539693 | 0.025592889 | low |
| TCGA-AC-A8OR | 0.91573329 | 5.621444541 | 0 | 1.619894799 | high |
| TCGA-S3-A6ZH | 1.0471271 | 0 | 0 | 0.337714099 | high |
| TCGA-E2-A14Z | 0.099518526 | 0.921347683 | 0 | 0.249189459 | high |
| TCGA-E2-A1IF | 0.235879742 | 0 | 0.435722418 | 0.172045142 | low |
| TCGA-D8-A27G | 0.075683674 | 0.73701 | 0.064424198 | 0.212257463 | high |
| TCGA-EW-A1PH | 0.34437204 | 0 | 1.36021926 | 0.410661358 | high |
| TCGA-E2-A14Y | 1.937915589 | 0 | 1.220011593 | 0.893721349 | high |
| TCGA-WT-AB41 | 0.213778785 | 0 | 0 | 0.068946845 | low |
| TCGA-AO-A1KQ | 0.153183708 | 0 | 0 | 0.049404029 | low |
| TCGA-D8-A1JU | 0.06916084 | 0 | 0.21533183 | 0.069733496 | low |
| TCGA-BH-A0DL | 1.947948702 | 0 | 3.055405123 | 1.301213329 | high |
| TCGA-E2-A56Z | 0.38624959 | 0 | 0.135790441 | 0.154479889 | low |
| TCGA-A7-A0D9 | 0.1425136 | 0 | 0.6201177 | 0.182547308 | low |
| TCGA-BH-A0DO | 0.115172317 | 4.060865926 | 0.032998966 | 1.001257638 | high |
| TCGA-A2-A259 | 0.138199077 | 0 | 0.348504094 | 0.121331326 | low |
| TCGA-AN-A0XP | 0.329121629 | 0 | 0.183126844 | 0.146481389 | low |
| TCGA-BH-A202 | 0.1248344 | 3.25462 | 0.6709016 | 0.954903276 | high |
| TCGA-A2-A0YJ | 0.560317433 | 0 | 3.328672006 | 0.913870187 | high |
| TCGA-B6-A0RH | 1.451422041 | 2.869422608 | 0.602750025 | 1.276974353 | high |
| TCGA-D8-A1XD | 0.134092728 | 0 | 0.303486099 | 0.110091491 | low |
| TCGA-A2-A0T7 | 0.94020132 | 0 | 0.56564848 | 0.427816325 | high |
| TCGA-AC-A2FG | 0.196293913 | 0 | 0.099830967 | 0.085296067 | low |
| TCGA-BH-AB28 | 0.129638994 | 0 | 0 | 0.041810508 | low |
| TCGA-B6-A0RE | 0.207801053 | 0 | 2.34088564 | 0.58261268 | high |
| TCGA-D8-A1JP | 1.695944463 | 1.583438681 | 1.392312532 | 1.226731291 | high |
| TCGA-S3-A6ZF | 0.728865049 | 0 | 0.229151161 | 0.285541726 | high |
| TCGA-AC-A3YJ | 0.187783925 | 0 | 0 | 0.060563115 | low |
| TCGA-A2-A0YI | 0.111332809 | 0 | 0 | 0.035906491 | low |
| TCGA-AN-A04C | 0.729714263 | 0 | 4.153705186 | 1.15022157 | high |
| TCGA-B6-A0RO | 0.241936632 | 0 | 0.014410182 | 0.081202097 | low |
| TCGA-A2-A0SX | 0.080207372 | 0.07878726 | 0.792483735 | 0.218981555 | high |
| TCGA-E2-A14R | 0.586837471 | 0 | 0 | 0.189263832 | low |
| TCGA-A7-A0DB | 0.322956913 | 0 | 0 | 0.104158419 | low |
| TCGA-EW-A1OX | 0.15099074 | 0 | 0 | 0.048696765 | low |
| TCGA-A7-A26F | 0.46837133 | 0.476797032 | 1.272225396 | 0.543617479 | high |
| TCGA-AC-A2QH | 0.363844035 | 0.852934734 | 1.228423415 | 0.588885959 | high |
| TCGA-AO-A1KR | 0.330175761 | 0 | 0.002749225 | 0.107092138 | low |
| TCGA-BH-A1ES | 0.850546483 | 0 | 0.305057554 | 0.341504629 | high |
| TCGA-B6-A0IE | 0.009077879 | 0 | 0 | 0.002927751 | low |
| TCGA-EW-A1P5 | 0.082752369 | 0 | 0.219690644 | 0.075077021 | low |
| TCGA-E9-A5UO | 0.53423789 | 0 | 0 | 0.172299683 | low |
| TCGA-HN-A2NL | 0.620234871 | 0 | 0.830080087 | 0.382864982 | high |
| TCGA-GM-A2DN | 0.749026985 | 0 | 0.350981124 | 0.318878012 | high |
| TCGA-C8-A12V | 0.495519834 | 0 | 0.02473507 | 0.165260577 | low |
| TCGA-EW-A1J2 | 0.096891392 | 0 | 0.391730088 | 0.117529759 | low |
| TCGA-AR-A1AR | 0.623536208 | 0 | 0.59047472 | 0.33115523 | high |
| TCGA-PL-A8LV | 1.550551 | 0 | 2.97177 | 1.154625521 | high |
| TCGA-D8-A1JF | 0 | 0 | 0.892869584 | 0.196659744 | high |
| TCGA-AC-A23E | 0 | 0.271335589 | 0.719104599 | 0.222320646 | high |
| TCGA-BH-A0DH | 0.246435587 | 0 | 0 | 0.07947915 | low |
| TCGA-C8-A12Y | 0.137534051 | 0 | 0.132499917 | 0.073540663 | low |
| TCGA-BH-A0BG | 0 | 0 | 0 | 0 | low |
| TCGA-BH-A18H | 0.28892111 | 0 | 0.123653754 | 0.120416827 | low |
| TCGA-D8-A1JD | 0.078469325 | 0.328884046 | 0 | 0.102801081 | low |
| TCGA-E9-A228 | 0.567057658 | 0 | 1.076819736 | 0.420060388 | high |
| TCGA-E9-A3HO | 0.481588555 | 2.20890758 | 0 | 0.675795023 | high |
| TCGA-A2-A0EV | 1.385604639 | 0 | 0.013525808 | 0.449857289 | high |
| TCGA-BH-A0EI | 0.050225864 | 0 | 0 | 0.01619859 | low |
| TCGA-AN-A0XW | 0.133981568 | 0 | 0.499929974 | 0.153323551 | low |
| TCGA-A8-A09A | 0.24121947 | 0 | 0.31712011 | 0.147644432 | low |
| TCGA-A1-A0SG | 0.516893 | 0 | 0 | 0.166705698 | low |
| TCGA-BH-A1F6 | 0.495181793 | 0.904019822 | 1.332583558 | 0.666223187 | high |
| TCGA-E9-A249 | 0.208956763 | 0 | 0 | 0.067391671 | low |
| TCGA-BH-A1F2 | 0 | 0 | 1.017761584 | 0.224167937 | high |
| TCGA-A8-A096 | 0 | 0 | 0.037738377 | 0.008312098 | low |
| TCGA-AR-A1AI | 0.145271891 | 0 | 0.231701827 | 0.097886032 | low |
| TCGA-AC-A3BB | 0.10623385 | 0 | 0.032499293 | 0.04142016 | low |
| TCGA-BH-A0WA | 0.10407502 | 0 | 0.37534705 | 0.116238127 | low |
| TCGA-AQ-A1H3 | 0.045122334 | 0 | 0.1297921 | 0.043140094 | low |
| TCGA-BH-A0E7 | 0.109635347 | 0 | 0.16546137 | 0.071802867 | low |
| TCGA-BH-A0RX | 0.193321662 | 2.130659638 | 0.074930879 | 0.580891386 | high |
| TCGA-AO-A03U | 0.1032788 | 0 | 1.39088 | 0.339658401 | high |
| TCGA-AR-A24L | 0.279597512 | 0 | 0.487342545 | 0.197514409 | high |
| TCGA-E2-A1IJ | 0.093571438 | 0.0764858 | 0.433761229 | 0.143738654 | low |
| TCGA-E2-A150 | 0.175884574 | 0 | 0.836636326 | 0.240999437 | high |
| TCGA-E2-A15H | 0.178573995 | 0 | 0.124717785 | 0.085062596 | low |
| TCGA-BH-A0E0 | 2.204971627 | 0 | 0.158643323 | 0.746078338 | high |
| TCGA-A2-A0D1 | 1.13899202 | 0 | 0.38810336 | 0.452823938 | high |
| TCGA-A8-A09D | 0.274611152 | 0 | 0.979003792 | 0.304197497 | high |
| TCGA-AQ-A1H2 | 0 | 0 | 0 | 0 | low |
| TCGA-D8-A1JH | 0.122593706 | 0 | 0.454142177 | 0.139565766 | low |
| TCGA-GM-A3NW | 0.129415912 | 0 | 0 | 0.041738561 | low |
| TCGA-A7-A0CD | 0 | 0 | 0 | 0 | low |
| TCGA-A7-A2KD | 0.04785459 | 0 | 0 | 0.015433819 | low |
| TCGA-AO-A03L | 0.12528207 | 0 | 0.15702136 | 0.074990207 | low |
| TCGA-AR-A2LR | 0.443204081 | 0.339646406 | 1.9965782 | 0.662727383 | high |
| TCGA-E2-A14T | 1.039766614 | 0 | 0.466309473 | 0.438047619 | high |
| TCGA-AR-A24U | 0.004793459 | 0 | 2.766476055 | 0.610878481 | high |
| TCGA-BH-A28Q | 0.024629087 | 0 | 0 | 0.007943248 | low |
| TCGA-C8-A3M7 | 0.185540919 | 0 | 0.044202044 | 0.069575471 | low |
| TCGA-BH-A0EE | 0.212941439 | 0 | 1.246564291 | 0.343239859 | high |
| TCGA-3C-AALK | 0.561202045 | 0 | 0.096227887 | 0.20219078 | high |
| TCGA-EW-A1OW | 0 | 0.6464112 | 1.29866204 | 0.438349036 | high |
| TCGA-E2-A108 | 0.012495417 | 1.02568226 | 0.467463627 | 0.34866872 | high |
| TCGA-A2-A0D4 | 0.21210356 | 0 | 0 | 0.06840656 | low |
| TCGA-E9-A1RA | 0.420680937 | 0 | 0.246265148 | 0.189917211 | low |
| TCGA-V7-A7HQ | 0.732117403 | 0 | 0 | 0.236118776 | high |
| TCGA-AR-A24X | 0.122481572 | 0 | 0.01302024 | 0.042369917 | low |
| TCGA-D8-A73X | 0.23268612 | 0.867123382 | 0.017816935 | 0.283285635 | high |
| TCGA-BH-A0W4 | 0.094946226 | 0 | 0.355307056 | 0.108880029 | low |
| TCGA-BH-A1ET | 0.014809425 | 0 | 0.044822227 | 0.014648618 | low |
| TCGA-BH-A0C7 | 0.241424414 | 0 | 1.194951724 | 0.341058073 | high |
| TCGA-LD-A7W5 | 0.920516465 | 0.200702765 | 0 | 0.344171024 | high |
| TCGA-D8-A1XK | 0.778532789 | 0.650049056 | 0.998460848 | 0.624173572 | high |
| TCGA-A2-A0T4 | 0.56841757 | 0 | 0.44753847 | 0.281896103 | high |
| TCGA-AN-A0FX | 0.2146156 | 0 | 3.812602374 | 0.908964685 | high |
| TCGA-A2-A0D3 | 0.091850934 | 0 | 0 | 0.029623295 | low |
| TCGA-D8-A1XS | 0 | 0 | 0.143637826 | 0.031637071 | low |
| TCGA-C8-A138 | 0.273511616 | 0 | 0.376972342 | 0.171241935 | low |
| TCGA-AR-A24R | 0.263775806 | 0 | 0 | 0.08507163 | low |
| TCGA-BH-A2L8 | 0.325569711 | 0.171076365 | 0 | 0.145311084 | low |
| TCGA-E2-A14V | 0.0695111 | 0 | 0.95229443 | 0.232166779 | high |
| TCGA-AO-A0JE | 0.159958815 | 0.02022195 | 0.434545982 | 0.152065207 | low |
| TCGA-A8-A06O | 0.062957567 | 0.054086132 | 0 | 0.03304884 | low |
| TCGA-AO-A0J4 | 0.229691517 | 0 | 0.415311156 | 0.165553647 | low |
| TCGA-EW-A1P3 | 0.149453489 | 0 | 0.066995924 | 0.062957222 | low |
| TCGA-B6-A0WT | 1.051402 | 0 | 0.1923435 | 0.381457598 | high |
| TCGA-C8-A26W | 0.519633786 | 0 | 0 | 0.167589642 | low |
| TCGA-AN-A041 | 0.01821216 | 0 | 0.49691013 | 0.115321053 | low |
| TCGA-D8-A1Y3 | 0 | 0 | 0 | 0 | low |
| TCGA-B6-A40B | 0.19999983 | 0 | 0.29518764 | 0.129519729 | low |
| TCGA-B6-A1KI | 0.120236884 | 0 | 0.206017124 | 0.084154663 | low |
| TCGA-A8-A090 | 0.02716303 | 0 | 0.112737704 | 0.03359162 | low |
| TCGA-BH-A0B6 | 0 | 0 | 0 | 0 | low |
| TCGA-A8-A09W | 0 | 0 | 0 | 0 | low |
| TCGA-AO-A03M | 0.070794575 | 0 | 0 | 0.022832306 | low |
| TCGA-E2-A1L6 | 0.075683674 | 0 | 0 | 0.024409113 | low |
| TCGA-AR-A0TQ | 0.069160838 | 0 | 0.021594036 | 0.027061614 | low |
| TCGA-B6-A0RP | 0 | 0 | 0.019958347 | 0.004395943 | low |
| TCGA-AN-A0FK | 0.3116542 | 5.018332882 | 0.061848482 | 1.296584132 | high |
| TCGA-E9-A22D | 0.141298104 | 0 | 0.01504159 | 0.048883743 | low |
| TCGA-BH-A18L | 3.966362942 | 0 | 0 | 1.279211175 | high |
| TCGA-E9-A1RG | 0.302881812 | 0 | 0.17294051 | 0.135775057 | low |
| TCGA-D8-A146 | 0.380729898 | 0 | 0.624671939 | 0.260378713 | high |
| TCGA-AO-A0JL | 0.314565404 | 1.920088498 | 0.921056257 | 0.756742374 | high |
| TCGA-EW-A6SB | 0.2802941 | 0 | 1.22111121 | 0.359355906 | high |
| TCGA-3C-AAAU | 0.60173335 | 0 | 0.11107026 | 0.218531849 | high |
| TCGA-GM-A2DF | 0.35907322 | 0.2108278 | 0.83594336 | 0.349604338 | high |
| TCGA-E2-A15J | 0.053302161 | 0 | 0 | 0.017190741 | low |
| TCGA-EW-A424 | 0.355601207 | 0.990899352 | 0 | 0.348168107 | high |
| TCGA-A8-A07Z | 0.42139623 | 0 | 0 | 0.135906566 | low |
| TCGA-BH-A0BF | 0.134537275 | 1.158208454 | 0.407554402 | 0.406060267 | high |
| TCGA-E2-A154 | 0.199788341 | 1.315417322 | 0.412033873 | 0.465133798 | high |
| TCGA-A8-A08T | 0.11415706 | 0 | 0 | 0.036817354 | low |
| TCGA-A8-A085 | 0.55224816 | 0 | 0.20612966 | 0.223509526 | high |
| TCGA-EW-A1P4 | 0 | 0 | 4.370048287 | 0.962528674 | high |
| TCGA-EW-A1J1 | 0.41170689 | 0 | 0 | 0.132781609 | low |
| TCGA-A8-A084 | 0.306007576 | 2.172678633 | 0.412928319 | 0.701580942 | high |
| TCGA-E9-A1R7 | 0.205591879 | 0 | 0.970929499 | 0.280159342 | high |
| TCGA-AO-A0JA | 1.055220658 | 0 | 0 | 0.340324392 | high |
| TCGA-A2-A1G4 | 0.620234871 | 4.707053509 | 0 | 1.309138069 | high |
| TCGA-C8-A12K | 0.05719638 | 0 | 1.01527482 | 0.242066898 | high |
| TCGA-A2-A0CK | 0.21388342 | 0.88218548 | 0 | 0.276846219 | high |

# Appendix 9

## **GO and KEGG enrichment analysis**

**Table 7a. GO enrichment analysis.**

| ONTOLOGY | Description | BgRatio | pvalue | qvalue |
| --- | --- | --- | --- | --- |
| BP | fatty acid metabolic process | 392/18862 | 1.37E-101 | 5.46E-99 |
| BP | acyl-CoA metabolic process | 105/18862 | 1.04E-59 | 1.37E-57 |
| BP | thioester metabolic process | 105/18862 | 1.04E-59 | 1.37E-57 |
| BP | fatty acid oxidation | 105/18862 | 3.91E-55 | 3.89E-53 |
| BP | nucleoside bisphosphate metabolic process | 140/18862 | 1.32E-54 | 7.49E-53 |
| BP | ribonucleoside bisphosphate metabolic process | 140/18862 | 1.32E-54 | 7.49E-53 |
| BP | purine nucleoside bisphosphate metabolic process | 140/18862 | 1.32E-54 | 7.49E-53 |
| BP | lipid oxidation | 110/18862 | 2.39E-54 | 1.19E-52 |
| BP | fatty acid biosynthetic process | 168/18862 | 1.78E-49 | 7.88E-48 |
| BP | monocarboxylic acid biosynthetic process | 224/18862 | 9.55E-47 | 3.60E-45 |
| BP | fatty acid beta-oxidation | 75/18862 | 9.95E-47 | 3.60E-45 |
| BP | fatty acid catabolic process | 106/18862 | 3.50E-46 | 1.16E-44 |
| BP | monocarboxylic acid catabolic process | 131/18862 | 3.40E-45 | 9.86E-44 |
| BP | fatty-acyl-CoA metabolic process | 42/18862 | 3.47E-45 | 9.86E-44 |
| BP | thioester biosynthetic process | 53/18862 | 1.78E-44 | 4.43E-43 |
| BP | acyl-CoA biosynthetic process | 53/18862 | 1.78E-44 | 4.43E-43 |
| BP | carboxylic acid biosynthetic process | 327/18862 | 3.89E-44 | 9.10E-43 |
| BP | organic acid biosynthetic process | 335/18862 | 9.84E-44 | 2.08E-42 |
| BP | nucleoside bisphosphate biosynthetic process | 67/18862 | 1.10E-43 | 2.08E-42 |
| BP | ribonucleoside bisphosphate biosynthetic process | 67/18862 | 1.10E-43 | 2.08E-42 |
| BP | purine nucleoside bisphosphate biosynthetic process | 67/18862 | 1.10E-43 | 2.08E-42 |
| BP | fatty acid derivative metabolic process | 82/18862 | 3.06E-43 | 5.54E-42 |
| BP | lipid modification | 270/18862 | 5.84E-42 | 1.01E-40 |
| BP | fatty-acyl-CoA biosynthetic process | 31/18862 | 2.19E-41 | 3.63E-40 |
| BP | small molecule catabolic process | 431/18862 | 3.45E-41 | 5.49E-40 |
| BP | organic acid catabolic process | 258/18862 | 6.88E-41 | 1.05E-39 |
| BP | carboxylic acid catabolic process | 243/18862 | 5.21E-40 | 7.68E-39 |
| BP | sulfur compound metabolic process | 378/18862 | 1.94E-38 | 2.76E-37 |
| BP | fatty acid derivative biosynthetic process | 62/18862 | 8.13E-38 | 1.12E-36 |
| BP | very long-chain fatty acid metabolic process | 34/18862 | 1.46E-37 | 1.94E-36 |
| BP | cellular lipid catabolic process | 230/18862 | 2.88E-37 | 3.68E-36 |
| BP | purine ribonucleotide metabolic process | 408/18862 | 2.96E-37 | 3.68E-36 |
| BP | ribonucleotide metabolic process | 425/18862 | 1.26E-36 | 1.52E-35 |
| BP | long-chain fatty-acyl-CoA biosynthetic process | 19/18862 | 2.12E-36 | 2.48E-35 |
| BP | long-chain fatty acid metabolic process | 120/18862 | 2.36E-36 | 2.68E-35 |
| BP | ribose phosphate metabolic process | 435/18862 | 2.88E-36 | 3.18E-35 |
| BP | purine nucleotide metabolic process | 441/18862 | 4.68E-36 | 5.03E-35 |
| BP | long-chain fatty-acyl-CoA metabolic process | 25/18862 | 7.92E-36 | 8.29E-35 |
| BP | purine-containing compound metabolic process | 460/18862 | 2.07E-35 | 2.12E-34 |
| BP | lipid catabolic process | 340/18862 | 1.40E-33 | 1.39E-32 |
| BP | purine ribonucleotide biosynthetic process | 175/18862 | 6.14E-32 | 5.95E-31 |
| BP | ribonucleotide biosynthetic process | 188/18862 | 4.04E-31 | 3.83E-30 |
| BP | ribose phosphate biosynthetic process | 195/18862 | 1.05E-30 | 9.74E-30 |
| BP | purine nucleotide biosynthetic process | 197/18862 | 1.37E-30 | 1.24E-29 |
| BP | purine-containing compound biosynthetic process | 208/18862 | 5.65E-30 | 5.00E-29 |
| BP | sulfur compound biosynthetic process | 193/18862 | 3.96E-29 | 3.42E-28 |
| BP | fatty acid elongation | 13/18862 | 1.85E-28 | 1.57E-27 |
| BP | nucleotide biosynthetic process | 264/18862 | 2.60E-27 | 2.15E-26 |
| BP | nucleoside phosphate biosynthetic process | 267/18862 | 3.46E-27 | 2.81E-26 |
| BP | ethanol oxidation | 12/18862 | 4.67E-26 | 3.71E-25 |
| BP | very long-chain fatty acid biosynthetic process | 13/18862 | 3.02E-25 | 2.36E-24 |
| BP | ethanol metabolic process | 22/18862 | 2.65E-21 | 2.03E-20 |
| BP | fatty acid beta-oxidation using acyl-CoA dehydrogenase | 11/18862 | 1.19E-17 | 8.96E-17 |
| BP | alcohol metabolic process | 373/18862 | 1.03E-15 | 7.62E-15 |
| BP | primary alcohol metabolic process | 94/18862 | 5.19E-15 | 3.75E-14 |
| BP | unsaturated fatty acid metabolic process | 115/18862 | 6.24E-14 | 4.41E-13 |
| BP | medium-chain fatty acid metabolic process | 14/18862 | 6.32E-14 | 4.41E-13 |
| BP | sphingolipid metabolic process | 167/18862 | 2.42E-13 | 1.66E-12 |
| BP | short-chain fatty acid metabolic process | 17/18862 | 3.55E-13 | 2.39E-12 |
| BP | sphingolipid biosynthetic process | 109/18862 | 1.08E-12 | 7.14E-12 |
| BP | linoleic acid metabolic process | 21/18862 | 2.09E-12 | 1.36E-11 |
| BP | peroxisome organization | 84/18862 | 2.26E-12 | 1.45E-11 |
| BP | membrane lipid metabolic process | 215/18862 | 6.13E-12 | 3.87E-11 |
| BP | alpha-linolenic acid metabolic process | 13/18862 | 8.06E-12 | 5.01E-11 |
| BP | peroxisomal transport | 72/18862 | 1.92E-11 | 1.17E-10 |
| BP | membrane lipid biosynthetic process | 148/18862 | 3.14E-11 | 1.89E-10 |
| BP | unsaturated fatty acid biosynthetic process | 51/18862 | 4.02E-11 | 2.39E-10 |
| BP | amino-acid betaine metabolic process | 17/18862 | 5.73E-11 | 3.36E-10 |
| BP | long-chain fatty acid transport | 72/18862 | 7.03E-10 | 4.06E-09 |
| BP | fatty acid transport | 150/18862 | 7.65E-10 | 4.35E-09 |
| BP | carnitine metabolic process | 13/18862 | 1.52E-09 | 8.49E-09 |
| BP | fatty acid transmembrane transport | 50/18862 | 1.63E-09 | 9.00E-09 |
| BP | fatty acid beta-oxidation using acyl-CoA oxidase | 15/18862 | 3.51E-09 | 1.91E-08 |
| BP | regulation of lipid metabolic process | 402/18862 | 1.33E-08 | 7.18E-08 |
| BP | protein targeting to peroxisome | 68/18862 | 1.49E-08 | 7.69E-08 |
| BP | protein localization to peroxisome | 68/18862 | 1.49E-08 | 7.69E-08 |
| BP | establishment of protein localization to peroxisome | 68/18862 | 1.49E-08 | 7.69E-08 |
| BP | diterpenoid metabolic process | 114/18862 | 2.80E-08 | 1.43E-07 |
| BP | olefinic compound metabolic process | 119/18862 | 3.93E-08 | 1.98E-07 |
| BP | retinol metabolic process | 47/18862 | 4.50E-08 | 2.24E-07 |
| BP | terpenoid metabolic process | 124/18862 | 5.42E-08 | 2.66E-07 |
| BP | cellular hormone metabolic process | 136/18862 | 1.11E-07 | 5.40E-07 |
| BP | isoprenoid metabolic process | 143/18862 | 1.64E-07 | 7.86E-07 |
| BP | retinoic acid metabolic process | 33/18862 | 2.61E-07 | 1.22E-06 |
| BP | regulation of fatty acid oxidation | 33/18862 | 2.61E-07 | 1.22E-06 |
| BP | hormone metabolic process | 218/18862 | 3.55E-07 | 1.64E-06 |
| BP | retinoid metabolic process | 108/18862 | 3.74E-07 | 1.71E-06 |
| BP | fatty acid derivative catabolic process | 16/18862 | 5.25E-07 | 2.37E-06 |
| BP | acetyl-CoA metabolic process | 38/18862 | 5.44E-07 | 2.43E-06 |
| BP | organic acid transport | 324/18862 | 1.11E-06 | 4.89E-06 |
| BP | steroid metabolic process | 329/18862 | 1.27E-06 | 5.55E-06 |
| BP | intracellular lipid transport | 47/18862 | 1.61E-06 | 6.98E-06 |
| BP | acetyl-CoA biosynthetic process | 22/18862 | 2.07E-06 | 8.86E-06 |
| BP | regulation of lipid biosynthetic process | 203/18862 | 2.34E-06 | 9.91E-06 |
| BP | regulation of fatty acid biosynthetic process | 54/18862 | 3.25E-06 | 1.35E-05 |
| BP | cholesterol metabolic process | 149/18862 | 3.27E-06 | 1.35E-05 |
| BP | positive regulation of cold-induced thermogenesis | 97/18862 | 3.50E-06 | 1.43E-05 |
| BP | regulation of fatty acid metabolic process | 98/18862 | 3.71E-06 | 1.51E-05 |
| BP | carboxylic acid transmembrane transport | 154/18862 | 4.07E-06 | 1.64E-05 |
| BP | organic acid transmembrane transport | 155/18862 | 4.25E-06 | 1.69E-05 |
| BP | secondary alcohol metabolic process | 158/18862 | 4.82E-06 | 1.90E-05 |
| BP | sterol metabolic process | 165/18862 | 6.41E-06 | 2.50E-05 |
| BP | cellular aldehyde metabolic process | 63/18862 | 7.01E-06 | 2.71E-05 |
| BP | temperature homeostasis | 171/18862 | 8.11E-06 | 3.10E-05 |
| BP | long-chain fatty acid biosynthetic process | 33/18862 | 1.12E-05 | 4.24E-05 |
| BP | carnitine shuttle | 11/18862 | 1.18E-05 | 4.44E-05 |
| BP | cellular ketone metabolic process | 254/18862 | 1.22E-05 | 4.52E-05 |
| BP | cholesterol biosynthetic process | 72/18862 | 1.35E-05 | 4.95E-05 |
| BP | secondary alcohol biosynthetic process | 72/18862 | 1.35E-05 | 4.95E-05 |
| BP | negative regulation of fatty acid oxidation | 12/18862 | 1.57E-05 | 5.69E-05 |
| BP | steroid biosynthetic process | 190/18862 | 1.61E-05 | 5.77E-05 |
| BP | organic hydroxy compound catabolic process | 77/18862 | 1.88E-05 | 6.68E-05 |
| BP | sterol biosynthetic process | 78/18862 | 2.00E-05 | 7.06E-05 |
| BP | ethanol catabolic process | 13/18862 | 2.04E-05 | 7.11E-05 |
| BP | lipid transport | 461/18862 | 2.46E-05 | 8.52E-05 |
| BP | coenzyme A metabolic process | 15/18862 | 3.22E-05 | 0.000110527 |
| BP | cold-induced thermogenesis | 144/18862 | 3.37E-05 | 0.000113586 |
| BP | regulation of cold-induced thermogenesis | 144/18862 | 3.37E-05 | 0.000113586 |
| BP | alpha-amino acid catabolic process | 88/18862 | 3.60E-05 | 0.00012029 |
| BP | primary alcohol catabolic process | 16/18862 | 3.95E-05 | 0.000131097 |
| BP | adaptive thermogenesis | 153/18862 | 4.73E-05 | 0.000155598 |
| BP | long-chain fatty acid import into cell | 17/18862 | 4.79E-05 | 0.000156102 |
| BP | branched-chain amino acid catabolic process | 20/18862 | 7.95E-05 | 0.000253086 |
| BP | peroxisomal membrane transport | 20/18862 | 7.95E-05 | 0.000253086 |
| BP | regulation of fatty acid beta-oxidation | 20/18862 | 7.95E-05 | 0.000253086 |
| BP | alcohol catabolic process | 55/18862 | 8.68E-05 | 0.000274127 |
| BP | branched-chain amino acid metabolic process | 23/18862 | 0.000122388 | 0.00038045 |
| BP | positive regulation of fatty acid biosynthetic process | 23/18862 | 0.000122388 | 0.00038045 |
| BP | regulation of cholesterol metabolic process | 61/18862 | 0.000130289 | 0.00040187 |
| BP | regulation of cellular ketone metabolic process | 185/18862 | 0.000135153 | 0.000413667 |
| BP | cellular modified amino acid metabolic process | 198/18862 | 0.000195535 | 0.000593911 |
| BP | positive regulation of fatty acid transport | 31/18862 | 0.000303135 | 0.000913755 |
| BP | negative regulation of fatty acid metabolic process | 34/18862 | 0.000399872 | 0.001187365 |
| BP | lipid import into cell | 34/18862 | 0.000399872 | 0.001187365 |
| BP | purine ribonucleotide catabolic process | 35/18862 | 0.000436027 | 0.001285134 |
| BP | positive regulation of lipid biosynthetic process | 85/18862 | 0.000468054 | 0.001369383 |
| BP | positive regulation of organic acid transport | 36/18862 | 0.000474217 | 0.001377289 |
| BP | alcohol biosynthetic process | 156/18862 | 0.000529544 | 0.001526832 |
| BP | positive regulation of fatty acid metabolic process | 38/18862 | 0.000556887 | 0.001594117 |
| BP | lipid homeostasis | 162/18862 | 0.000628534 | 0.00178636 |
| BP | ribonucleotide catabolic process | 40/18862 | 0.000648245 | 0.001829313 |
| BP | organic hydroxy compound biosynthetic process | 251/18862 | 0.000690716 | 0.001935438 |
| BP | long-chain fatty acid import across plasma membrane | 10/18862 | 0.000781979 | 0.002131131 |
| BP | coenzyme A biosynthetic process | 10/18862 | 0.000781979 | 0.002131131 |
| BP | positive regulation of fatty acid beta-oxidation | 10/18862 | 0.000781979 | 0.002131131 |
| BP | neuron projection maintenance | 10/18862 | 0.000781979 | 0.002131131 |
| BP | cellular ketone body metabolic process | 11/18862 | 0.000953123 | 0.002579882 |
| BP | electron transport chain | 178/18862 | 0.000960401 | 0.002582018 |
| BP | purine nucleotide catabolic process | 46/18862 | 0.000977945 | 0.002611539 |
| BP | cellular modified amino acid biosynthetic process | 48/18862 | 0.001107501 | 0.002918335 |
| BP | regulation of fatty acid transport | 48/18862 | 0.001107501 | 0.002918335 |
| BP | lysine catabolic process | 12/18862 | 0.001140603 | 0.002927999 |
| BP | aldehyde catabolic process | 12/18862 | 0.001140603 | 0.002927999 |
| BP | cellular response to aldehyde | 12/18862 | 0.001140603 | 0.002927999 |
| BP | ketone body metabolic process | 12/18862 | 0.001140603 | 0.002927999 |
| BP | positive regulation of lipid localization | 111/18862 | 0.001273902 | 0.003249223 |
| BP | alpha-amino acid metabolic process | 191/18862 | 0.001314185 | 0.003327586 |
| BP | purine-containing compound catabolic process | 51/18862 | 0.001321351 | 0.003327586 |
| BP | lysine metabolic process | 13/18862 | 0.00134428 | 0.003364037 |
| BP | macromolecule depalmitoylation | 14/18862 | 0.001564017 | 0.003889463 |
| BP | sulfur compound catabolic process | 56/18862 | 0.001732297 | 0.004254764 |
| BP | protein homotetramerization | 56/18862 | 0.001732297 | 0.004254764 |
| BP | positive regulation of fatty acid oxidation | 15/18862 | 0.001799678 | 0.004349799 |
| BP | regulation of unsaturated fatty acid biosynthetic process | 15/18862 | 0.001799678 | 0.004349799 |
| BP | regulation of steroid metabolic process | 122/18862 | 0.001803786 | 0.004349799 |
| BP | regulation of organic acid transport | 60/18862 | 0.002112662 | 0.005033634 |
| BP | regulation of lipid catabolic process | 60/18862 | 0.002112662 | 0.005033634 |
| BP | myelin maintenance | 17/18862 | 0.002318228 | 0.00549054 |
| BP | regulation of small molecule metabolic process | 437/18862 | 0.00253414 | 0.005966395 |
| BP | cardiolipin metabolic process | 18/18862 | 0.002600849 | 0.006087436 |
| BP | protein targeting | 441/18862 | 0.002666058 | 0.00620357 |
| BP | negative regulation of fatty acid biosynthetic process | 20/18862 | 0.003212117 | 0.007430724 |
| BP | nucleotide catabolic process | 73/18862 | 0.003691312 | 0.008489906 |
| BP | positive regulation of lipid metabolic process | 150/18862 | 0.00380548 | 0.008702187 |
| BP | aspartate family amino acid catabolic process | 23/18862 | 0.004242112 | 0.009645224 |
| BP | protein tetramerization | 83/18862 | 0.005290451 | 0.011892896 |
| BP | nucleoside phosphate catabolic process | 83/18862 | 0.005290451 | 0.011892896 |
| BP | positive regulation of lipid transport | 85/18862 | 0.005652812 | 0.012636091 |
| BP | positive regulation of lipid catabolic process | 27/18862 | 0.00582111 | 0.012939604 |
| BP | energy derivation by oxidation of organic compounds | 278/18862 | 0.006564495 | 0.014510989 |
| BP | sterol homeostasis | 95/18862 | 0.007685987 | 0.016896208 |
| BP | regulation of lipid localization | 189/18862 | 0.008545599 | 0.018682687 |
| BP | negative regulation of lipid metabolic process | 100/18862 | 0.008844513 | 0.019230519 |
| BP | negative regulation of small molecule metabolic process | 101/18862 | 0.009087813 | 0.019652137 |
| BP | negative regulation of cytokine production involved in inflammatory response | 35/18862 | 0.009654011 | 0.020652043 |
| BP | negative regulation of reactive oxygen species biosynthetic process | 35/18862 | 0.009654011 | 0.020652043 |
| BP | phosphatidylglycerol metabolic process | 37/18862 | 0.010747113 | 0.022867486 |
| BP | mitochondrial transmembrane transport | 109/18862 | 0.011175505 | 0.023652524 |
| BP | estrogen metabolic process | 38/18862 | 0.011313237 | 0.023817341 |
| BP | nucleobase-containing small molecule biosynthetic process | 115/18862 | 0.012908391 | 0.027032531 |
| BP | cellular amino acid metabolic process | 331/18862 | 0.013294255 | 0.027694839 |
| BP | respiratory electron transport chain | 117/18862 | 0.013518253 | 0.028014801 |
| BP | triglyceride biosynthetic process | 42/18862 | 0.013705483 | 0.028255645 |
| BP | xenobiotic metabolic process | 120/18862 | 0.01446348 | 0.029664652 |
| BP | icosanoid metabolic process | 123/18862 | 0.015445387 | 0.031355296 |
| BP | reactive oxygen species biosynthetic process | 123/18862 | 0.015445387 | 0.031355296 |
| BP | myelination | 124/18862 | 0.015780868 | 0.031873727 |
| BP | cellular response to xenobiotic stimulus | 125/18862 | 0.016120448 | 0.032395158 |
| BP | ensheathment of neurons | 126/18862 | 0.016464131 | 0.032754956 |
| BP | axon ensheathment | 126/18862 | 0.016464131 | 0.032754956 |
| BP | regulation of cholesterol biosynthetic process | 48/18862 | 0.01766449 | 0.034795088 |
| BP | regulation of sterol biosynthetic process | 48/18862 | 0.01766449 | 0.034795088 |
| BP | response to xenobiotic stimulus | 131/18862 | 0.018244251 | 0.035760056 |
| BP | aspartate family amino acid metabolic process | 50/18862 | 0.019079402 | 0.036852396 |
| BP | neutral lipid biosynthetic process | 50/18862 | 0.019079402 | 0.036852396 |
| BP | acylglycerol biosynthetic process | 50/18862 | 0.019079402 | 0.036852396 |
| BP | positive regulation of small molecule metabolic process | 141/18862 | 0.02211426 | 0.04250796 |
| BP | icosanoid biosynthetic process | 56/18862 | 0.023597636 | 0.045141227 |
| BP | arachidonic acid metabolic process | 59/18862 | 0.026005338 | 0.049273273 |
| BP | negative regulation of lipid biosynthetic process | 59/18862 | 0.026005338 | 0.049273273 |
| CC | mitochondrial matrix | 476/19520 | 1.81E-29 | 7.80E-28 |
| CC | peroxisome | 136/19520 | 3.27E-16 | 4.71E-15 |
| CC | microbody | 136/19520 | 3.27E-16 | 4.71E-15 |
| CC | peroxisomal membrane | 61/19520 | 3.00E-12 | 2.59E-11 |
| CC | microbody membrane | 61/19520 | 3.00E-12 | 2.59E-11 |
| CC | integral component of organelle membrane | 371/19520 | 2.49E-11 | 1.79E-10 |
| CC | integral component of endoplasmic reticulum membrane | 157/19520 | 4.16E-11 | 2.57E-10 |
| CC | intrinsic component of endoplasmic reticulum membrane | 165/19520 | 7.13E-11 | 3.69E-10 |
| CC | intrinsic component of organelle membrane | 402/19520 | 7.69E-11 | 3.69E-10 |
| CC | peroxisomal matrix | 51/19520 | 6.09E-08 | 2.39E-07 |
| CC | microbody lumen | 51/19520 | 6.09E-08 | 2.39E-07 |
| CC | integral component of peroxisomal membrane | 15/19520 | 2.91E-05 | 9.02E-05 |
| CC | intrinsic component of peroxisomal membrane | 15/19520 | 2.91E-05 | 9.02E-05 |
| CC | nucleoid | 44/19520 | 3.13E-05 | 9.02E-05 |
| CC | mitochondrial nucleoid | 44/19520 | 3.13E-05 | 9.02E-05 |
| CC | mitochondrial outer membrane | 195/19520 | 0.000149698 | 0.000403791 |
| CC | organelle outer membrane | 220/19520 | 0.000287254 | 0.00072293 |
| CC | outer membrane | 222/19520 | 0.000301515 | 0.00072293 |
| CC | mitochondrial inner membrane | 493/19520 | 0.004079773 | 0.009267074 |
| MF | CoA-ligase activity | 27/18337 | 5.22E-27 | 3.57E-25 |
| MF | acid-thiol ligase activity | 31/18337 | 6.81E-26 | 2.33E-24 |
| MF | ligase activity, forming carbon-sulfur bonds | 41/18337 | 8.75E-24 | 2.00E-22 |
| MF | transferase activity, transferring acyl groups | 254/18337 | 6.88E-23 | 1.18E-21 |
| MF | transferase activity, transferring acyl groups other than amino-acyl groups | 225/18337 | 1.52E-22 | 2.09E-21 |
| MF | fatty acid ligase activity | 22/18337 | 3.61E-21 | 4.12E-20 |
| MF | fatty acid synthase activity | 12/18337 | 7.83E-20 | 7.65E-19 |
| MF | C-acyltransferase activity | 20/18337 | 2.49E-19 | 2.13E-18 |
| MF | oxidoreductase activity, acting on CH-OH group of donors | 130/18337 | 1.13E-17 | 8.57E-17 |
| MF | oxidoreductase activity, acting on the CH-OH group of donors, NAD or NADP as acceptor | 120/18337 | 1.27E-16 | 8.71E-16 |
| MF | ligase activity | 163/18337 | 3.58E-16 | 2.23E-15 |
| MF | alcohol dehydrogenase [NAD(P)+] activity | 10/18337 | 2.73E-15 | 1.56E-14 |
| MF | oxidoreductase activity, acting on the CH-CH group of donors | 59/18337 | 7.38E-14 | 3.76E-13 |
| MF | long-chain fatty acid-CoA ligase activity | 14/18337 | 7.70E-14 | 3.76E-13 |
| MF | thiolester hydrolase activity | 41/18337 | 1.13E-13 | 5.14E-13 |
| MF | oxidoreductase activity, acting on the aldehyde or oxo group of donors | 44/18337 | 2.26E-13 | 9.65E-13 |
| MF | aldehyde dehydrogenase [NAD(P)+] activity | 16/18337 | 2.55E-13 | 1.03E-12 |
| MF | acyl-CoA hydrolase activity | 19/18337 | 1.11E-12 | 4.22E-12 |
| MF | butyrate-CoA ligase activity | 10/18337 | 1.18E-12 | 4.25E-12 |
| MF | oxidoreductase activity, acting on the aldehyde or oxo group of donors, NAD or NADP as acceptor | 36/18337 | 2.51E-12 | 8.04E-12 |
| MF | CoA hydrolase activity | 21/18337 | 2.55E-12 | 8.04E-12 |
| MF | acyl-CoA dehydrogenase activity | 11/18337 | 2.59E-12 | 8.04E-12 |
| MF | hydro-lyase activity | 64/18337 | 8.17E-12 | 2.43E-11 |
| MF | palmitoyl-CoA hydrolase activity | 14/18337 | 1.66E-11 | 4.74E-11 |
| MF | aldehyde dehydrogenase (NAD+) activity | 15/18337 | 2.76E-11 | 7.56E-11 |
| MF | carbon-oxygen lyase activity | 79/18337 | 5.80E-11 | 1.53E-10 |
| MF | flavin adenine dinucleotide binding | 81/18337 | 7.30E-11 | 1.85E-10 |
| MF | retinol dehydrogenase activity | 20/18337 | 2.10E-10 | 5.14E-10 |
| MF | enoyl-CoA hydratase activity | 10/18337 | 3.45E-10 | 8.14E-10 |
| MF | arachidonate-CoA ligase activity | 11/18337 | 6.30E-10 | 1.44E-09 |
| MF | myristoyl-CoA hydrolase activity | 13/18337 | 1.74E-09 | 3.85E-09 |
| MF | fatty-acyl-CoA binding | 21/18337 | 2.68E-08 | 5.74E-08 |
| MF | fatty acid derivative binding | 22/18337 | 3.46E-08 | 7.17E-08 |
| MF | acyl-CoA binding | 24/18337 | 5.55E-08 | 1.12E-07 |
| MF | ATPase activity | 478/18337 | 1.40E-07 | 2.74E-07 |
| MF | lyase activity | 194/18337 | 1.67E-07 | 3.18E-07 |
| MF | oxidoreductase activity, acting on the CH-CH group of donors, oxygen as acceptor | 10/18337 | 9.39E-06 | 1.74E-05 |
| MF | intramolecular oxidoreductase activity, transposing C=C bonds | 14/18337 | 2.81E-05 | 5.06E-05 |
| MF | acetyltransferase activity | 95/18337 | 5.94E-05 | 0.000104148 |
| MF | NAD binding | 53/18337 | 8.36E-05 | 0.000143001 |
| MF | sulfur compound binding | 260/18337 | 0.000139825 | 0.000233341 |
| MF | electron transfer activity | 138/18337 | 0.00034321 | 0.000559113 |
| MF | intramolecular oxidoreductase activity | 50/18337 | 0.001352102 | 0.002151448 |
| MF | palmitoyl-(protein) hydrolase activity | 14/18337 | 0.001653293 | 0.002513779 |
| MF | palmitoyl hydrolase activity | 14/18337 | 0.001653293 | 0.002513779 |
| MF | AMP binding | 15/18337 | 0.001902256 | 0.002829443 |
| MF | retinol binding | 16/18337 | 0.002167867 | 0.003155909 |
| MF | long-chain fatty acid transporter activity | 17/18337 | 0.002449978 | 0.003492294 |
| MF | estradiol 17-beta-dehydrogenase activity | 18/18337 | 0.002748446 | 0.003837787 |
| MF | arachidonic acid monooxygenase activity | 20/18337 | 0.003393871 | 0.004644245 |
| MF | carboxylic ester hydrolase activity | 143/18337 | 0.003545936 | 0.00475719 |
| MF | monocarboxylic acid binding | 71/18337 | 0.00369248 | 0.004858527 |
| MF | oxidoreductase activity, acting on the CH-CH group of donors, NAD or NADP as acceptor | 26/18337 | 0.00570785 | 0.007368625 |
| MF | amide binding | 391/18337 | 0.007222283 | 0.009151041 |
| MF | steroid dehydrogenase activity, acting on the CH-OH group of donors, NAD or NADP as acceptor | 30/18337 | 0.007554134 | 0.009397488 |
| MF | steroid dehydrogenase activity | 34/18337 | 0.009633207 | 0.011769896 |
| MF | retinoid binding | 37/18337 | 0.011340205 | 0.013377738 |
| MF | fatty acid binding | 37/18337 | 0.011340205 | 0.013377738 |
| MF | isoprenoid binding | 38/18337 | 0.011936647 | 0.013842677 |
| MF | oxidoreductase activity, acting on paired donors, with incorporation or reduction of molecular oxygen, NAD(P)H as one donor, and incorporation of one atom of oxygen | 48/18337 | 0.018623514 | 0.021237341 |
| MF | O-acyltransferase activity | 53/18337 | 0.022435183 | 0.025164571 |
| MF | vitamin binding | 145/18337 | 0.025566534 | 0.028214341 |
| MF | ATPase-coupled transmembrane transporter activity | 58/18337 | 0.026539408 | 0.028823083 |
| MF | primary active transmembrane transporter activity | 61/18337 | 0.029136483 | 0.0311492 |
| MF | isomerase activity | 157/18337 | 0.03132995 | 0.032978895 |
| MF | quaternary ammonium group transmembrane transporter activity | 10/18337 | 0.042791307 | 0.0436989 |
| MF | testosterone dehydrogenase [NAD(P)] activity | 10/18337 | 0.042791307 | 0.0436989 |
| MF | carboxylic acid binding | 184/18337 | 0.046567547 | 0.04657556 |
| MF | potassium ion binding | 11/18337 | 0.046969661 | 0.04657556 |

**Table 7b. KEGG enrichment analysis.**

| ID | Description | BgRatio | pvalue | qvalue |
| --- | --- | --- | --- | --- |
| hsa00071 | Fatty acid degradation | 43/8164 | 6.08E-71 | 1.02E-69 |
| hsa01212 | Fatty acid metabolism | 57/8164 | 2.56E-68 | 2.16E-67 |
| hsa00062 | Fatty acid elongation | 27/8164 | 6.18E-48 | 3.47E-47 |
| hsa01040 | Biosynthesis of unsaturated fatty acids | 27/8164 | 2.66E-29 | 1.12E-28 |
| hsa00620 | Pyruvate metabolism | 47/8164 | 7.56E-24 | 2.54E-23 |
| hsa00280 | Valine, leucine and isoleucine degradation | 48/8164 | 1.16E-23 | 3.26E-23 |
| hsa00061 | Fatty acid biosynthesis | 18/8164 | 5.55E-19 | 1.33E-18 |
| hsa00650 | Butanoate metabolism | 27/8164 | 2.25E-18 | 4.73E-18 |
| hsa00010 | Glycolysis / Gluconeogenesis | 67/8164 | 5.70E-16 | 1.07E-15 |
| hsa00380 | Tryptophan metabolism | 42/8164 | 1.28E-15 | 2.16E-15 |
| hsa00410 | beta-Alanine metabolism | 31/8164 | 8.75E-14 | 1.34E-13 |
| hsa04936 | Alcoholic liver disease | 142/8164 | 1.06E-13 | 1.49E-13 |
| hsa00310 | Lysine degradation | 63/8164 | 2.66E-13 | 3.45E-13 |
| hsa00640 | Propanoate metabolism | 32/8164 | 6.74E-12 | 8.10E-12 |
| hsa04146 | Peroxisome | 82/8164 | 7.21E-12 | 8.10E-12 |
| hsa03320 | PPAR signaling pathway | 75/8164 | 5.65E-11 | 5.95E-11 |
| hsa00350 | Tyrosine metabolism | 36/8164 | 2.80E-08 | 2.78E-08 |
| hsa00340 | Histidine metabolism | 22/8164 | 1.33E-06 | 1.25E-06 |
| hsa00830 | Retinol metabolism | 68/8164 | 2.57E-06 | 2.28E-06 |
| hsa04920 | Adipocytokine signaling pathway | 69/8164 | 2.84E-06 | 2.39E-06 |
| hsa00982 | Drug metabolism - cytochrome P450 | 72/8164 | 3.80E-06 | 3.05E-06 |
| hsa00980 | Metabolism of xenobiotics by cytochrome P450 | 78/8164 | 6.52E-06 | 4.99E-06 |
| hsa00053 | Ascorbate and aldarate metabolism | 30/8164 | 6.81E-06 | 4.99E-06 |
| hsa01200 | Carbon metabolism | 115/8164 | 9.33E-06 | 6.55E-06 |
| hsa00330 | Arginine and proline metabolism | 50/8164 | 8.78E-05 | 5.91E-05 |
| hsa00630 | Glyoxylate and dicarboxylate metabolism | 30/8164 | 0.000150247 | 9.73E-05 |
| hsa00561 | Glycerolipid metabolism | 62/8164 | 0.000246101 | 0.000153513 |
| hsa04216 | Ferroptosis | 41/8164 | 0.00051439 | 0.000309407 |
| hsa00770 | Pantothenate and CoA biosynthesis | 21/8164 | 0.000879378 | 0.00051071 |
| hsa04913 | Ovarian steroidogenesis | 51/8164 | 0.011309163 | 0.006349004 |
| hsa00900 | Terpenoid backbone biosynthesis | 23/8164 | 0.018598684 | 0.010104548 |
| hsa04714 | Thermogenesis | 232/8164 | 0.019465257 | 0.010244872 |

**Appendix 10**

**gene set enrichment analyses (GSEA)**

**Table 8a. GSEA of high rish.**

| NAME | SIZE | ES | NES | NOM p-val | FDR q-val |
| --- | --- | --- | --- | --- | --- |
| KEGG_GLYCOSPHINGOLIPID_BIOSYNTHESIS_LACTO_AND_NEOLACTO_SERIES | 26 | 0.75272006 | 2.369457 | 0 | 0 |
| KEGG_PATHOGENIC_ESCHERICHIA_COLI_INFECTION | 56 | 0.6675864 | 2.2340863 | 0 | 0.001389191 |
| KEGG_CELL_CYCLE | 125 | 0.6158247 | 2.0141478 | 0.007889546 | 0.03607177 |
| KEGG_P53_SIGNALING_PATHWAY | 68 | 0.53257555 | 1.9548221 | 0.003968254 | 0.049757753 |
| KEGG_GALACTOSE_METABOLISM | 25 | 0.64042395 | 1.9131734 | 0.001976285 | 0.06407539 |
| KEGG_DNA_REPLICATION | 36 | 0.7491455 | 1.8942229 | 0.005940594 | 0.06596925 |
| KEGG_VIBRIO_CHOLERAE_INFECTION | 54 | 0.5265463 | 1.8905877 | 0.01 | 0.05841668 |
| KEGG_CYSTEINE_AND_METHIONINE_METABOLISM | 34 | 0.5705846 | 1.8881056 | 0.002008032 | 0.05248372 |
| KEGG_GLYCOLYSIS_GLUCONEOGENESIS | 61 | 0.5279733 | 1.8690523 | 0.005882353 | 0.05555214 |
| KEGG_FC_GAMMA_R_MEDIATED_PHAGOCYTOSIS | 96 | 0.49094397 | 1.7960458 | 0.01632653 | 0.095634595 |
| KEGG_GLYOXYLATE_AND_DICARBOXYLATE_METABOLISM | 16 | 0.65949476 | 1.779005 | 0.01183432 | 0.09865493 |
| KEGG_PENTOSE_PHOSPHATE_PATHWAY | 27 | 0.58753455 | 1.7687465 | 0.00996016 | 0.09852541 |
| KEGG_PROTEASOME | 46 | 0.6663546 | 1.7652639 | 0.022267206 | 0.09320721 |
| KEGG_TERPENOID_BACKBONE_BIOSYNTHESIS | 15 | 0.6907596 | 1.7596813 | 0.009652509 | 0.090465955 |
| KEGG_BLADDER_CANCER | 42 | 0.49863622 | 1.7508724 | 0.007827789 | 0.09001206 |
| KEGG_GLIOMA | 65 | 0.45914012 | 1.7303138 | 0.005964215 | 0.09852175 |
| KEGG_STARCH_AND_SUCROSE_METABOLISM | 51 | 0.511722 | 1.6812216 | 0.018255578 | 0.13335921 |
| KEGG_ONE_CARBON_POOL_BY_FOLATE | 17 | 0.6150082 | 1.6684897 | 0.018072288 | 0.13737196 |
| KEGG_GLYCOSAMINOGLYCAN_BIOSYNTHESIS_KERATAN_SULFATE | 15 | 0.58741915 | 1.6655489 | 0.026262626 | 0.13242029 |
| KEGG_OOCYTE_MEIOSIS | 113 | 0.444321 | 1.6644825 | 0.020366598 | 0.12708147 |
| KEGG_HOMOLOGOUS_RECOMBINATION | 28 | 0.5741793 | 1.6586597 | 0.05210421 | 0.12550929 |
| KEGG_AMINOACYL_TRNA_BIOSYNTHESIS | 41 | 0.56317455 | 1.6421467 | 0.052734375 | 0.13326098 |
| KEGG_PYRIMIDINE_METABOLISM | 98 | 0.4407893 | 1.6412748 | 0.047984645 | 0.12840833 |
| KEGG_PURINE_METABOLISM | 159 | 0.3823636 | 1.6326503 | 0.02918288 | 0.12898305 |
| KEGG_NATURAL_KILLER_CELL_MEDIATED_CYTOTOXICITY | 132 | 0.4795061 | 1.6281515 | 0.06490872 | 0.12804815 |
| KEGG_RNA_POLYMERASE | 29 | 0.539188 | 1.6256269 | 0.045544554 | 0.124709345 |
| KEGG_BASAL_TRANSCRIPTION_FACTORS | 35 | 0.5054122 | 1.6047032 | 0.03550296 | 0.13770896 |
| KEGG_PROGESTERONE_MEDIATED_OOCYTE_MATURATION | 85 | 0.42060915 | 1.5976045 | 0.04255319 | 0.13882849 |
| KEGG_PRION_DISEASES | 35 | 0.492632 | 1.5878712 | 0.06300813 | 0.14272684 |
| KEGG_GAP_JUNCTION | 90 | 0.39582488 | 1.5761541 | 0.03244275 | 0.14811893 |
| KEGG_REGULATION_OF_ACTIN_CYTOSKELETON | 213 | 0.38706425 | 1.5746028 | 0.023904383 | 0.14465697 |
| KEGG_AMINO_SUGAR_AND_NUCLEOTIDE_SUGAR_METABOLISM | 44 | 0.46359187 | 1.5580252 | 0.058252428 | 0.153775 |
| KEGG_RNA_DEGRADATION | 59 | 0.46590498 | 1.557866 | 0.078431375 | 0.14924884 |
| KEGG_AXON_GUIDANCE | 129 | 0.37957326 | 1.5438343 | 0.023762377 | 0.15512346 |
| KEGG_MELANOGENESIS | 101 | 0.37834537 | 1.5414344 | 0.027944112 | 0.15290934 |
| KEGG_AMYOTROPHIC_LATERAL_SCLEROSIS_ALS | 53 | 0.4178602 | 1.5373861 | 0.035785288 | 0.152674 |
| KEGG_PYRUVATE_METABOLISM | 40 | 0.46057823 | 1.5360153 | 0.0407767 | 0.15004326 |
| KEGG_SPLICEOSOME | 127 | 0.4826556 | 1.5316294 | 0.10240964 | 0.14984475 |
| KEGG_DRUG_METABOLISM_OTHER_ENZYMES | 51 | 0.4438657 | 1.5250245 | 0.030487806 | 0.15162313 |
| KEGG_MISMATCH_REPAIR | 23 | 0.5914221 | 1.5145932 | 0.08582834 | 0.15702944 |
| KEGG_FRUCTOSE_AND_MANNOSE_METABOLISM | 34 | 0.46207574 | 1.4842811 | 0.056862745 | 0.1781885 |
| KEGG_DORSO_VENTRAL_AXIS_FORMATION | 24 | 0.4816913 | 1.4677448 | 0.08016032 | 0.18830703 |
| KEGG_CALCIUM_SIGNALING_PATHWAY | 178 | 0.3402273 | 1.4666762 | 0.030534351 | 0.1852207 |
| KEGG_PATHWAYS_IN_CANCER | 325 | 0.33825648 | 1.4610356 | 0.044715445 | 0.1859028 |
| KEGG_GLYCOSAMINOGLYCAN_BIOSYNTHESIS_HEPARAN_SULFATE | 26 | 0.45089847 | 1.4485362 | 0.05232558 | 0.1936192 |
| KEGG_GLYCOSPHINGOLIPID_BIOSYNTHESIS_GANGLIO_SERIES | 15 | 0.50628716 | 1.4424068 | 0.09416196 | 0.19538385 |
| KEGG_LONG_TERM_POTENTIATION | 70 | 0.3771458 | 1.4412838 | 0.046558704 | 0.19232285 |
| KEGG_GLYCOSAMINOGLYCAN_BIOSYNTHESIS_CHONDROITIN_SULFATE | 22 | 0.5155912 | 1.4367696 | 0.119760476 | 0.1926405 |
| KEGG_PENTOSE_AND_GLUCURONATE_INTERCONVERSIONS | 28 | 0.50713736 | 1.4340979 | 0.08216433 | 0.19051127 |
| KEGG_VIRAL_MYOCARDITIS | 68 | 0.46952525 | 1.4305751 | 0.15690376 | 0.18978718 |
| KEGG_RIBOFLAVIN_METABOLISM | 16 | 0.47467634 | 1.4158981 | 0.10638298 | 0.19966455 |
| KEGG_EPITHELIAL_CELL_SIGNALING_IN_HELICOBACTER_PYLORI_INFECTION | 68 | 0.37461367 | 1.412438 | 0.1 | 0.19928193 |
| KEGG_LYSINE_DEGRADATION | 44 | 0.4141325 | 1.4081415 | 0.1295938 | 0.19934851 |
| KEGG_GLUTATHIONE_METABOLISM | 49 | 0.4243904 | 1.3908099 | 0.08851224 | 0.21231514 |
| KEGG_TRYPTOPHAN_METABOLISM | 40 | 0.41948313 | 1.389412 | 0.09848485 | 0.21006447 |
| KEGG_CITRATE_CYCLE_TCA_CYCLE | 31 | 0.48230338 | 1.3840942 | 0.15369649 | 0.2119537 |
| KEGG_LONG_TERM_DEPRESSION | 70 | 0.34333497 | 1.3819742 | 0.08092485 | 0.21038696 |
| KEGG_MATURITY_ONSET_DIABETES_OF_THE_YOUNG | 25 | 0.45680743 | 1.3785669 | 0.09580839 | 0.21023224 |
| KEGG_ADHERENS_JUNCTION | 73 | 0.3669199 | 1.3551836 | 0.116 | 0.22968543 |
| KEGG_CELL_ADHESION_MOLECULES_CAMS | 131 | 0.41081163 | 1.3534747 | 0.19502075 | 0.22786468 |
| KEGG_NOD_LIKE_RECEPTOR_SIGNALING_PATHWAY | 62 | 0.40710387 | 1.3507105 | 0.168357 | 0.22691248 |
| KEGG_GNRH_SIGNALING_PATHWAY | 101 | 0.3234163 | 1.3415966 | 0.08829569 | 0.23250458 |
| KEGG_TOLL_LIKE_RECEPTOR_SIGNALING_PATHWAY | 102 | 0.37973025 | 1.3405775 | 0.17892644 | 0.23011889 |
| KEGG_ALZHEIMERS_DISEASE | 166 | 0.35992113 | 1.3393244 | 0.1409002 | 0.22756502 |
| KEGG_NON_SMALL_CELL_LUNG_CANCER | 54 | 0.37466618 | 1.3383751 | 0.13121273 | 0.22489186 |
| KEGG_LEUKOCYTE_TRANSENDOTHELIAL_MIGRATION | 116 | 0.3723901 | 1.3347166 | 0.15957446 | 0.22529934 |
| KEGG_ARGININE_AND_PROLINE_METABOLISM | 54 | 0.36925733 | 1.327814 | 0.10337972 | 0.22886094 |
| KEGG_WNT_SIGNALING_PATHWAY | 151 | 0.30129537 | 1.3249918 | 0.108949415 | 0.22856611 |
| KEGG_MELANOMA | 71 | 0.3348503 | 1.3198162 | 0.10192308 | 0.23003784 |
| KEGG_NICOTINATE_AND_NICOTINAMIDE_METABOLISM | 24 | 0.40635687 | 1.3188425 | 0.13806707 | 0.22761439 |
| KEGG_BASAL_CELL_CARCINOMA | 55 | 0.35828245 | 1.3123205 | 0.14075631 | 0.2307881 |
| KEGG_CYTOKINE_CYTOKINE_RECEPTOR_INTERACTION | 264 | 0.36221263 | 1.310625 | 0.17409766 | 0.22914627 |
| KEGG_PANCREATIC_CANCER | 70 | 0.34477857 | 1.2923615 | 0.1456693 | 0.2443955 |
| KEGG_NOTCH_SIGNALING_PATHWAY | 47 | 0.35473406 | 1.2781757 | 0.16633266 | 0.2559465 |
| KEGG_STEROID_BIOSYNTHESIS | 17 | 0.48673603 | 1.269294 | 0.20077972 | 0.26201716 |
| KEGG_ANTIGEN_PROCESSING_AND_PRESENTATION | 81 | 0.42383513 | 1.2584472 | 0.26574802 | 0.27009514 |
| KEGG_N_GLYCAN_BIOSYNTHESIS | 46 | 0.37919593 | 1.2527742 | 0.20939335 | 0.27306107 |
| KEGG_NEUROTROPHIN_SIGNALING_PATHWAY | 126 | 0.31510547 | 1.2494214 | 0.19335938 | 0.2734818 |
| KEGG_LEISHMANIA_INFECTION | 70 | 0.42788768 | 1.2429904 | 0.2851153 | 0.27738968 |
| KEGG_ERBB_SIGNALING_PATHWAY | 87 | 0.31759167 | 1.2415012 | 0.19087137 | 0.2751041 |
| KEGG_VEGF_SIGNALING_PATHWAY | 76 | 0.31178987 | 1.2339343 | 0.1756487 | 0.2796893 |
| KEGG_ECM_RECEPTOR_INTERACTION | 84 | 0.37309867 | 1.2257832 | 0.2651072 | 0.28545418 |
| KEGG_HUNTINGTONS_DISEASE | 182 | 0.3350443 | 1.2250776 | 0.24117647 | 0.2828374 |
| KEGG_GLYCINE_SERINE_AND_THREONINE_METABOLISM | 31 | 0.35685036 | 1.2143484 | 0.20038168 | 0.29112098 |
| KEGG_CHEMOKINE_SIGNALING_PATHWAY | 188 | 0.3308161 | 1.192163 | 0.2774327 | 0.31329328 |
| KEGG_NEUROACTIVE_LIGAND_RECEPTOR_INTERACTION | 272 | 0.2686105 | 1.1920956 | 0.16498993 | 0.30976212 |
| KEGG_PARKINSONS_DISEASE | 130 | 0.37458706 | 1.1839201 | 0.29083666 | 0.31551883 |
| KEGG_STEROID_HORMONE_BIOSYNTHESIS | 55 | 0.34363997 | 1.1791601 | 0.22554891 | 0.31773528 |
| KEGG_TIGHT_JUNCTION | 131 | 0.29490823 | 1.1747372 | 0.22782259 | 0.3193948 |
| KEGG_SMALL_CELL_LUNG_CANCER | 84 | 0.31430018 | 1.1608566 | 0.26086956 | 0.33214268 |
| KEGG_GLYCOSAMINOGLYCAN_DEGRADATION | 21 | 0.39611155 | 1.1520268 | 0.28761905 | 0.33945027 |
| KEGG_HEDGEHOG_SIGNALING_PATHWAY | 56 | 0.29778284 | 1.1441702 | 0.2397541 | 0.34505996 |
| KEGG_TYPE_I_DIABETES_MELLITUS | 41 | 0.45948952 | 1.1356833 | 0.36419752 | 0.35139033 |
| KEGG_GRAFT_VERSUS_HOST_DISEASE | 37 | 0.5009312 | 1.1229315 | 0.3862213 | 0.36309636 |
| KEGG_GLYCEROLIPID_METABOLISM | 49 | 0.30371037 | 1.121016 | 0.298 | 0.36149827 |
| KEGG_RIG_I_LIKE_RECEPTOR_SIGNALING_PATHWAY | 71 | 0.3114441 | 1.1187899 | 0.33072406 | 0.36103114 |
| KEGG_SYSTEMIC_LUPUS_ERYTHEMATOSUS | 137 | 0.33044392 | 1.1175325 | 0.33604887 | 0.35897064 |
| KEGG_FOCAL_ADHESION | 199 | 0.29708767 | 1.1042329 | 0.31790745 | 0.37193298 |
| KEGG_AUTOIMMUNE_THYROID_DISEASE | 50 | 0.4019073 | 1.0932332 | 0.39387757 | 0.38148367 |
| KEGG_APOPTOSIS | 87 | 0.30251276 | 1.0921748 | 0.35655737 | 0.3788484 |
| KEGG_LYSOSOME | 121 | 0.29406264 | 1.074464 | 0.3605313 | 0.39741984 |
| KEGG_ALLOGRAFT_REJECTION | 35 | 0.48643386 | 1.0677419 | 0.4528302 | 0.40120703 |
| KEGG_TASTE_TRANSDUCTION | 51 | 0.30540743 | 1.0621867 | 0.38446215 | 0.40428966 |
| KEGG_PORPHYRIN_AND_CHLOROPHYLL_METABOLISM | 41 | 0.33896747 | 1.0571762 | 0.3697318 | 0.40661997 |
| KEGG_OXIDATIVE_PHOSPHORYLATION | 132 | 0.34489185 | 1.0562468 | 0.41910332 | 0.403777 |
| KEGG_CARDIAC_MUSCLE_CONTRACTION | 79 | 0.31092426 | 1.0560237 | 0.37058824 | 0.4001871 |
| KEGG_RETINOL_METABOLISM | 64 | 0.2930557 | 1.050045 | 0.34747475 | 0.40385205 |
| KEGG_O_GLYCAN_BIOSYNTHESIS | 30 | 0.321764 | 1.0455252 | 0.38589212 | 0.40571302 |
| KEGG_HEMATOPOIETIC_CELL_LINEAGE | 85 | 0.3308486 | 1.0323777 | 0.40430108 | 0.41830844 |
| KEGG_PROTEIN_EXPORT | 24 | 0.37372953 | 1.0267563 | 0.4417178 | 0.42115325 |
| KEGG_SELENOAMINO_ACID_METABOLISM | 26 | 0.32237956 | 1.0246251 | 0.44 | 0.42001727 |
| KEGG_ARRHYTHMOGENIC_RIGHT_VENTRICULAR_CARDIOMYOPATHY_ARVC | 74 | 0.28210905 | 1.0227174 | 0.39717743 | 0.4187273 |
| KEGG_ASCORBATE_AND_ALDARATE_METABOLISM | 25 | 0.37834835 | 1.0113354 | 0.46777546 | 0.43034375 |
| KEGG_ALANINE_ASPARTATE_AND_GLUTAMATE_METABOLISM | 32 | 0.29345515 | 1.0006523 | 0.4520548 | 0.4402363 |
| KEGG_B_CELL_RECEPTOR_SIGNALING_PATHWAY | 75 | 0.29860672 | 0.9709088 | 0.47454175 | 0.4748505 |
| KEGG_METABOLISM_OF_XENOBIOTICS_BY_CYTOCHROME_P450 | 69 | 0.2734097 | 0.9613008 | 0.50499004 | 0.48463145 |
| KEGG_BASE_EXCISION_REPAIR | 35 | 0.3283922 | 0.9572567 | 0.47609562 | 0.4856479 |
| KEGG_INTESTINAL_IMMUNE_NETWORK_FOR_IGA_PRODUCTION | 46 | 0.36231053 | 0.9300981 | 0.5072464 | 0.51920724 |
| KEGG_PRIMARY_IMMUNODEFICIENCY | 35 | 0.36997333 | 0.9117844 | 0.5329341 | 0.54012245 |
| KEGG_T_CELL_RECEPTOR_SIGNALING_PATHWAY | 108 | 0.2682474 | 0.90423644 | 0.53099173 | 0.5465342 |
| KEGG_PHENYLALANINE_METABOLISM | 18 | 0.31207135 | 0.8885114 | 0.59922177 | 0.5645578 |
| KEGG_CYTOSOLIC_DNA_SENSING_PATHWAY | 55 | 0.26704833 | 0.87704736 | 0.6020202 | 0.57714003 |
| KEGG_OLFACTORY_TRANSDUCTION | 387 | 0.20775886 | 0.6839039 | 0.86 | 0.845112 |

**Table 8b. GSEA of low rish.**

| NAME | SIZE | ES | NES | NOM p-val | FDR q-val |
| --- | --- | --- | --- | --- | --- |
| KEGG_GLYCOSYLPHOSPHATIDYLINOSITOL_GPI_ANCHOR_BIOSYNTHESIS | 25 | -0.671854 | -1.9157305 | 0 | 0.13507728 |
| KEGG_VASOPRESSIN_REGULATED_WATER_REABSORPTION | 44 | -0.5395747 | -1.8546885 | 0.008438818 | 0.124209374 |
| KEGG_PEROXISOME | 78 | -0.5087469 | -1.8055733 | 0.016359918 | 0.12894452 |
| KEGG_SPHINGOLIPID_METABOLISM | 39 | -0.46887222 | -1.5987812 | 0.008368201 | 0.42086542 |
| KEGG_ENDOCYTOSIS | 181 | -0.37706453 | -1.5828878 | 0.028397566 | 0.3688099 |
| KEGG_ABC_TRANSPORTERS | 44 | -0.4513914 | -1.5808427 | 0.03877551 | 0.31065613 |
| KEGG_SNARE_INTERACTIONS_IN_VESICULAR_TRANSPORT | 38 | -0.4637952 | -1.5801835 | 0.024621213 | 0.26736087 |
| KEGG_VALINE_LEUCINE_AND_ISOLEUCINE_DEGRADATION | 44 | -0.4932295 | -1.568109 | 0.052419353 | 0.25054738 |
| KEGG_ENDOMETRIAL_CANCER | 52 | -0.41492838 | -1.513753 | 0.04263566 | 0.30256736 |
| KEGG_UBIQUITIN_MEDIATED_PROTEOLYSIS | 135 | -0.38501406 | -1.4874775 | 0.08966862 | 0.31328815 |
| KEGG_BIOSYNTHESIS_OF_UNSATURATED_FATTY_ACIDS | 22 | -0.48824129 | -1.4765891 | 0.08523908 | 0.302117 |
| KEGG_VASCULAR_SMOOTH_MUSCLE_CONTRACTION | 115 | -0.35772267 | -1.4752905 | 0.055776894 | 0.2784888 |
| KEGG_FATTY_ACID_METABOLISM | 42 | -0.43433994 | -1.4702572 | 0.07926829 | 0.26349723 |
| KEGG_PROPANOATE_METABOLISM | 33 | -0.46832725 | -1.4695005 | 0.092402466 | 0.24543935 |
| KEGG_PHOSPHATIDYLINOSITOL_SIGNALING_SYSTEM | 76 | -0.38948557 | -1.4548303 | 0.08853119 | 0.24694592 |
| KEGG_MTOR_SIGNALING_PATHWAY | 52 | -0.38982075 | -1.4449296 | 0.06746032 | 0.24294524 |
| KEGG_INSULIN_SIGNALING_PATHWAY | 136 | -0.35236272 | -1.4364161 | 0.053465348 | 0.23877561 |
| KEGG_ALDOSTERONE_REGULATED_SODIUM_REABSORPTION | 42 | -0.4127753 | -1.4304708 | 0.07961165 | 0.2318599 |
| KEGG_GLYCEROPHOSPHOLIPID_METABOLISM | 76 | -0.35338053 | -1.4254689 | 0.052083332 | 0.22504303 |
| KEGG_COLORECTAL_CANCER | 62 | -0.37359616 | -1.4066832 | 0.10097087 | 0.23436686 |
| KEGG_TYROSINE_METABOLISM | 42 | -0.40033787 | -1.3914742 | 0.08686869 | 0.2396853 |
| KEGG_INOSITOL_PHOSPHATE_METABOLISM | 54 | -0.3904118 | -1.3882173 | 0.12650603 | 0.23211564 |
| KEGG_TYPE_II_DIABETES_MELLITUS | 47 | -0.3781486 | -1.3642087 | 0.11067194 | 0.24752918 |
| KEGG_PRIMARY_BILE_ACID_BIOSYNTHESIS | 16 | -0.45481867 | -1.3439076 | 0.13232104 | 0.2619501 |
| KEGG_MAPK_SIGNALING_PATHWAY | 267 | -0.3018386 | -1.3339846 | 0.08617234 | 0.2633998 |
| KEGG_BUTANOATE_METABOLISM | 34 | -0.4008251 | -1.3184311 | 0.13821138 | 0.2713734 |
| KEGG_PANTOTHENATE_AND_COA_BIOSYNTHESIS | 16 | -0.45466304 | -1.2916912 | 0.17706238 | 0.29363406 |
| KEGG_HISTIDINE_METABOLISM | 29 | -0.38104978 | -1.2667776 | 0.15042374 | 0.31269217 |
| KEGG_ALPHA_LINOLENIC_ACID_METABOLISM | 19 | -0.4228166 | -1.2618976 | 0.18664047 | 0.30803806 |
| KEGG_PROSTATE_CANCER | 89 | -0.3197987 | -1.227749 | 0.18145162 | 0.3404696 |
| KEGG_NITROGEN_METABOLISM | 23 | -0.3814877 | -1.2167687 | 0.17221135 | 0.34413263 |
| KEGG_JAK_STAT_SIGNALING_PATHWAY | 155 | -0.30641317 | -1.1848389 | 0.25612053 | 0.37505072 |
| KEGG_RENAL_CELL_CARCINOMA | 70 | -0.3095475 | -1.1729335 | 0.2520661 | 0.38028258 |
| KEGG_OTHER_GLYCAN_DEGRADATION | 16 | -0.42946595 | -1.1590232 | 0.31656185 | 0.38730475 |
| KEGG_CHRONIC_MYELOID_LEUKEMIA | 73 | -0.30856985 | -1.1518645 | 0.26452905 | 0.38580897 |
| KEGG_ARACHIDONIC_ACID_METABOLISM | 58 | -0.31481743 | -1.1364514 | 0.29482073 | 0.39575478 |
| KEGG_BETA_ALANINE_METABOLISM | 22 | -0.35885158 | -1.1258451 | 0.30607966 | 0.39962333 |
| KEGG_TGF_BETA_SIGNALING_PATHWAY | 86 | -0.29608232 | -1.1183326 | 0.28880158 | 0.3993846 |
| KEGG_FC_EPSILON_RI_SIGNALING_PATHWAY | 79 | -0.29387546 | -1.1144317 | 0.28294572 | 0.39441076 |
| KEGG_REGULATION_OF_AUTOPHAGY | 35 | -0.34436575 | -1.1136172 | 0.33131313 | 0.38587663 |
| KEGG_PROXIMAL_TUBULE_BICARBONATE_RECLAMATION | 23 | -0.3548479 | -1.0978941 | 0.326 | 0.3971954 |
| KEGG_THYROID_CANCER | 29 | -0.33080542 | -1.0892572 | 0.3205945 | 0.39838445 |
| KEGG_ACUTE_MYELOID_LEUKEMIA | 57 | -0.28938845 | -1.0474569 | 0.3954918 | 0.44589517 |
| KEGG_PPAR_SIGNALING_PATHWAY | 69 | -0.28556734 | -1.0458223 | 0.3920792 | 0.43785426 |
| KEGG_DILATED_CARDIOMYOPATHY | 90 | -0.28772363 | -1.0453211 | 0.37451738 | 0.42901328 |
| KEGG_ETHER_LIPID_METABOLISM | 33 | -0.29354882 | -1.0254852 | 0.39685658 | 0.44787917 |
| KEGG_HYPERTROPHIC_CARDIOMYOPATHY_HCM | 83 | -0.29248488 | -1.0248294 | 0.41617358 | 0.4393228 |
| KEGG_ADIPOCYTOKINE_SIGNALING_PATHWAY | 66 | -0.2839066 | -1.0166318 | 0.40490797 | 0.44174 |
| KEGG_NUCLEOTIDE_EXCISION_REPAIR | 44 | -0.32387987 | -1.0028127 | 0.4493927 | 0.4515684 |
| KEGG_RENIN_ANGIOTENSIN_SYSTEM | 17 | -0.33555892 | -0.97458 | 0.4745098 | 0.48137906 |
| KEGG_DRUG_METABOLISM_CYTOCHROME_P450 | 71 | -0.26846692 | -0.9719706 | 0.49583334 | 0.47556567 |
| KEGG_COMPLEMENT_AND_COAGULATION_CASCADES | 69 | -0.27674225 | -0.9328657 | 0.5214724 | 0.5219876 |
| KEGG_LINOLEIC_ACID_METABOLISM | 29 | -0.27088326 | -0.890085 | 0.59655833 | 0.5736836 |
| KEGG_ASTHMA | 28 | -0.25389785 | -0.63587266 | 0.78867924 | 0.9146809 |
| KEGG_RIBOSOME | 88 | -0.23141053 | -0.5288774 | 0.8191057 | 0.96873826 |
